# Group 11 Borataalkene Complexes: Models for Alkene Activation

**DOI:** 10.1002/anie.202100919

**Published:** 2021-05-03

**Authors:** Nicholas A. Phillips, Richard Y. Kong, Andrew J. P. White, Mark R. Crimmin

**Affiliations:** ^1^ Department of Chemistry, Molecular Sciences Research Hub Imperial College London 82 Wood Lane, Shepherds Bush London W12 0BZ UK

**Keywords:** borataalkene, boron, coinage metal, gold catalysis, metal boryl complexes

## Abstract

A series of linear late transition metal (M=Cu, Ag, Au and Zn) complexes featuring a side‐on [B=C]^−^ containing ligand have been isolated and characterised. The [B=C]^−^ moiety is isoelectronic with the C=C system of an alkene. Comparison across the series shows that in the solid‐state, deviation between the η^2^ and η^1^ coordination mode occurs. A related zinc complex containing two [B=C]^−^ ligands was prepared as a further point of comparison for the η^1^ coordination mode. The bonding in these new complexes has been interrogated by computational techniques (QTAIM, NBO, ETS‐NOCV) and rationalised in terms of the Dewar–Chatt–Duncanson model. The combined structural and computational data provide unique insight into catalytically relevant linear d^10^ complexes of Cu, Ag and Au. Slippage is proposed to play a key role in catalytic reactions of alkenes through disruption and polarisation of the π‐system. Through the preparation and analysis of a consistent series of group 11 complexes, we show that variation of the metal can impact the coordination mode and hence substrate activation.

## Graphical abstract

A series of neutral coinage‐metal boryl complexes featuring a side‐on [B=C]^−^ containing ligand have been isolated and characterised. The compounds are isoelectronic with alkene complexes, which are crucial intermediates in catalysis. Both the strength of binding and the propensity for deviation toward η^1^ coordination are more favourable for Au over Ag and Cu.

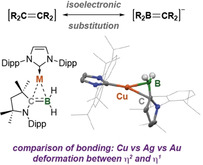

## Introduction

Alkene complexes of transition metals were among the first organometallic compounds reported.^[1]^ Coordination of alkene ligands typically occurs through a symmetric η^2^‐coordination mode involving a side‐on approach to metal. Alkene binding is synonymous with the activation of this substrate during catalytic processes.[^2^, ^3^, ^4^, ^5^, ^6^] For example, linear d^10^ of Cu^I^, Ag^I^ and Au^I^ complexes have been reported for a wealth of synthetic transformations; including ring‐expansions,^[7]^ cyclisations,[^8^, ^9^] hydroarylation,[^10^, ^11^, ^12^] hydroamination,[^13^, ^14^] hydroalkyoxylation,^[15]^ and carbonylation reactions.^[16]^ These reactions are believed to share a common mechanistic step in which binding of the alkene to the metal facilitates attack by a nucleophile.[^8^, ^17^, ^18^, ^19^, ^20^] An earlier theoretical analysis suggested that symmetrically bound alkene complexes are actually deactivated toward external nucleophiles and that slippage from an η^2^ to η^1^ coordination mode is crucial to activate the C=C bond and facilitate orbital overlap in the transition state for nucleophilic attack.^[21]^

**Figure 1:**
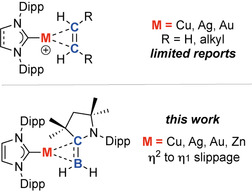
Structurally characterised examples of coordination of the isoelectronic fragments [C=C] and [B=C]^−^ to group 11 metals.

Due to their relevance as potential catalytic intermediates, a number of linear Au^I^ alkene complexes have been isolated and structurally characterised,[^22^, ^23^, ^24^, ^25^, ^26^, ^27^, ^28^, ^29^, ^30^, ^31^, ^32^, ^33^] as have related three‐coordinate trigonal planar species.^[34]^ In contrast, examples of crystallographically characterised linear Cu^I^ and Ag^I^ alkene complexes are extremely rare (Figure 1).[^35^, ^36^] The paucity of data has meant that the comparison of bonding in a complete series of alkene complexes of the group 11 triad has not been possible. There is limited experimental evidence to show how modulation of the metal influences alkene binding, including the ability to adopt η^2^ or η^1^ coordination modes believed to be so important in key bond making steps during catalysis.

In this paper, we consider the isoelectronic substitution of the C=C bond with a [B=C]^−^ bond and the binding of this fragment to a series of group 11 and 12 metals. The [B=C]^−^ moiety is found within borataalkene compounds of the form [R_2_B=CR_2_]^−^. The synthesis of borataalkenes has been achieved by a number of methods, the principal approach involving the deprotonation of the parent boranes [R_2_B−CR_2_H].[^37^, ^38^, ^39^, ^40^] Crystallographically characterised examples of borataalkenes were first reported by Power and co‐workers in the late 1980s, short B=C bond lengths of 1.4–1.5 Å were taken as an indication of double character.[^41^, ^42^] More recently a number of structurally characterised borataalkane compounds has been reported.[^43^, ^44^, ^45^, ^46^] Coordination of [B=C]^−^ moieties to transition metals is limited. Pioneering examples include those of Nöth and co‐workers who described the coordination of a fluorenylidene borane to transition metal carbonyl complexes,[^47^, ^48^, ^49^, ^50^] and those of Piers and co‐workers who reported methylidene borane complexes of tantalum and titanium along with their alkene‐like reactivity.[^51^, ^52^, ^53^, ^54^, ^55^, ^56^, ^57^] Related coordination complexes of alkylidene boranes have also been reported.^[58]^ To the best of our knowledge, group 11 complexes of borataalkene ligands are limited to a single example involving coordination of a 9‐borataphenanthrene anion to Au^I^.^[59]^

**Figure 2:**
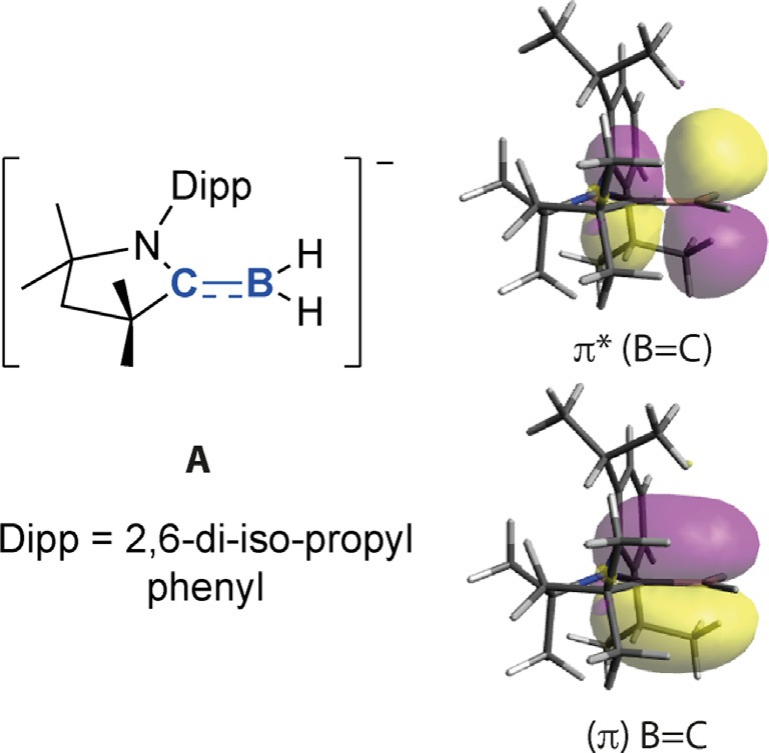
DFT optimised structure of Anion A. Calculated (π) B=C and (π *) B=C natural bond orbitals (this work).

Recently Braunschweig and co‐workers reported the lithium boryl compound [cAAC⋅BH_2_]Li (**1**) which can be isolated and is stable under ambient conditions.[^60^, ^61^, ^62^] An underappreciated characteristic of the anionic component [cAAC⋅BH_2_]^−^ (**A**) is its potential for B=C π‐bonding and borataalkene character (Figure 2). DFT calculations on this species (ωB97xD/6‐31G**/SDDAll) demonstrate that the B=C bond length (1.45 Å) is consistent with its assignment as a borataalkene. Natural bond orbital (NBO) calculations reveal that this is a polarised π‐system. The B=C Wiberg bond index (WBI) is 1.64 and natural charges show localisation of more charge on the carbon atom (−0.32) relative to boron (−0.15). Key natural bond orbitals of the π‐system are visualised in Figure 2. Despite the polarisation of the π‐system, it should be noted that the contributions to the NBOs are relatively even: 66 % pAO carbon; 34 % pAO boron (bonding NBO) and 34 % pAO carbon;66 % pAO boron (anti‐bonding NBO).

Herein we describe the side‐on coordination of the [B=C]^−^ bond of [(cAAC)BH_2_]^−^ to linear d^10^ metal fragments (M=Cu, Ag, Au and Zn). The bonding has been interrogated by a combination of experimental (^1^H, ^11^B, ^13^C NMR and IR spectroscopy) and computational techniques (QTAIM, NBO, ETS‐NOCV). Comparison across the series of group 11 complexes shows that a spectrum of coordination between η^2^ and η^1^ is observed; with η^1^ coordination becoming more favorable for Au > Ag > Cu. The data potentially provide new insight into catalytically relevant isoelectronic alkene complexes of Cu, Ag and Au.

## Results and Discussion

### Synthesis

**Scheme 1:**
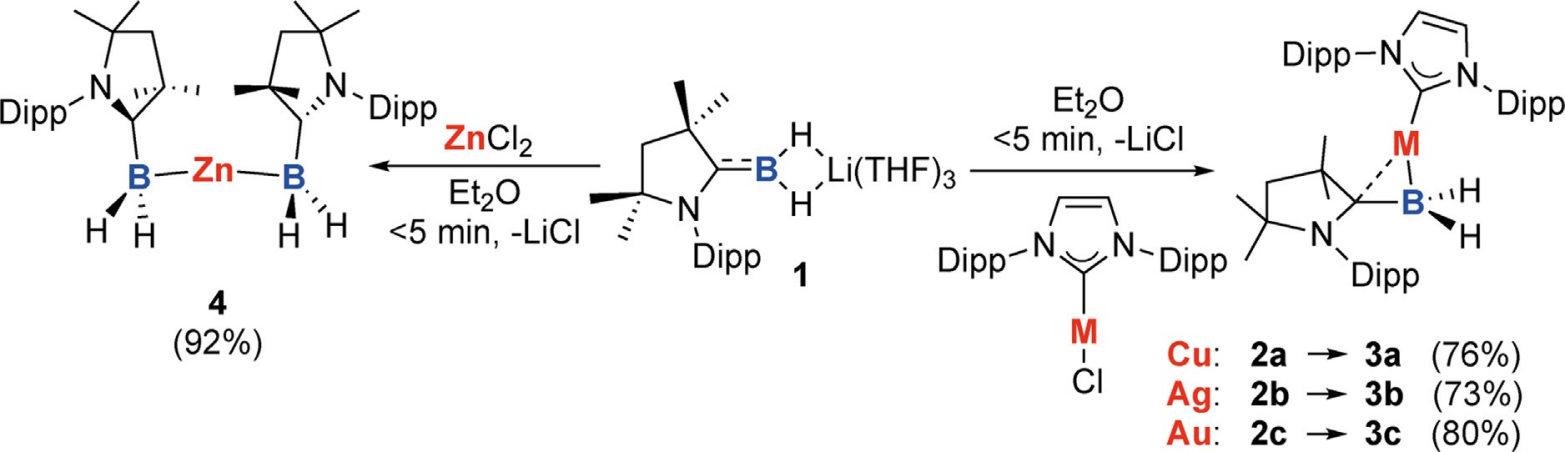
Synthesis of the metal boryl complexes **3 a**–**c** and **4**; yields in parentheses. Dipp=2,6‐di‐*iso*‐propylphenyl.

Reaction of **1** with the group 11 metal complexes [M(IPr)Cl] (IPr=1,3‐*bis*(2,6‐diisopropylphenyl)imidazol‐2‐ylidene; M=Cu, Ag, Au; **2 a**–**c**), in Et_2_O at 25 °C leads to a clean salt‐metathesis reaction and the formation of the corresponding group 11 complexes (**3 a**–**c**) in 73–80 % yield (Scheme 1). **3 a**–**c** are soluble in hydrocarbon and ether solvents. They are stable as solids at 25 °C but decompose slowly (**3 a** and **3 c**) or rapidly (**3 b**) in solution under ambient light precipitating the metal from solution. The formation of **3 a**–**c** was confirmed by ^11^B NMR spectroscopy as demonstrated by a shift in the resonance from **1** (δ_B_=−3.6 ppm) to higher field upon reaction to form **3 a**–**c** (δ_B_=−7.4 to −13.5 ppm). Infrared spectroscopy of **3 a**–**c** reveal B‐H stretches of ν_BH_=2345–2411 cm^−1^ which lie between reported stretching frequencies for sp^2^(B−H), ν_BH_=2467–2564 cm^−1^,[^63^, ^64^] and sp^3^(B−H), ν_BH_=2273–2383 cm^−1^.^[65]^ The analogous Zn complex, [Zn{BH_2_(cAAC)}_2_] (**4**, Scheme 1) can also be prepared by a salt‐metathesis route and shows B−H stretches (ν_BH_=2373, 2420 cm^−1^) that are marginally higher frequency than **3 a**–**c**.

### Solid‐State Structures

**Figure 3:**
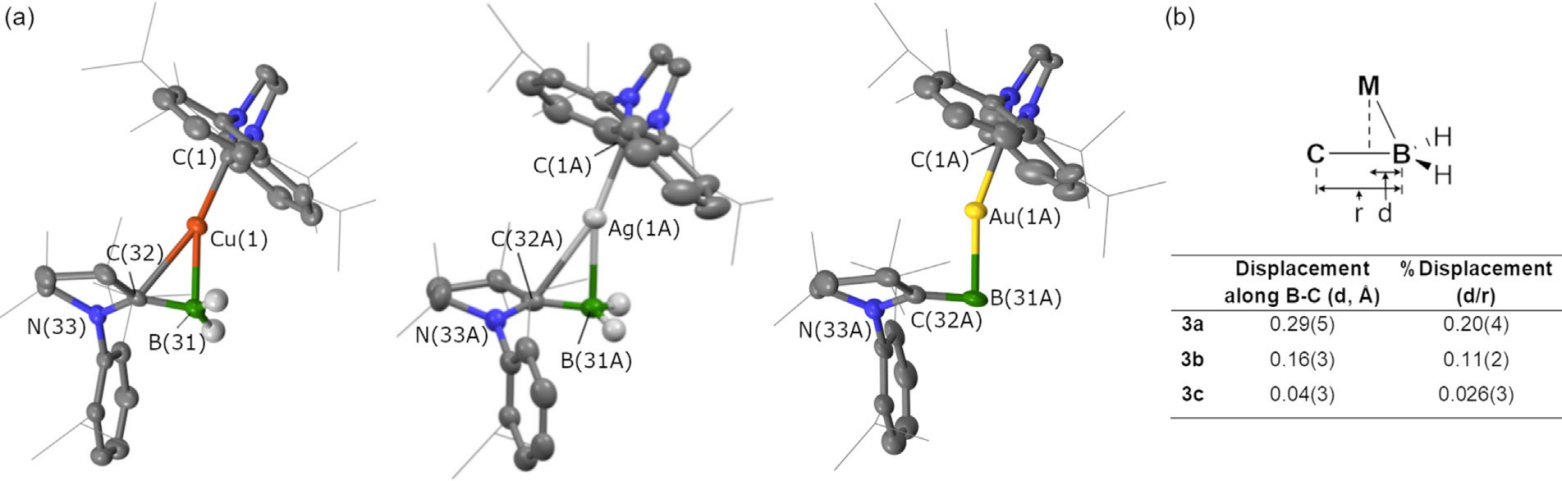
a) X‐ray diffraction determined structures for **3 a**–**c**. Key structural parameters (distances in Å, angles in °): **3 a**—Cu(1)–C(1) 1.944(2), Cu(1)–B(31) 2.121(2), Cu(1)–C(32) 2.411(2), B(31)–C(32) 1.469(3), N(33)–C(32) 1.406(6); C(1)‐Cu(1)‐B(31) 156.39(8), Cu(1)‐B(31)‐C(32) 82.2(1), Σ_angles_N(33) 360(2); **3 b**—Ag(1A)–C(1A) 2.151(4), Ag(1A)–B(31A) 2.287(3), Ag(1A)–C(32A) 2.633(3), B(31A)–C(32A) 1.473(5), N(33A)–C(32A) 1.413(5); C(1A)‐Ag(1A)‐B(31A) 158.5(1), Ag(1A)‐B(31A)‐C(32A) 86.0(2), Σ_angles_N(33A) 360(1); **3 c**—Au(1A)–C(1A) 2.088(9), Au(1A)–B(31A) 2.23(1), Au(1A)–C(32A) 2.68(1), B(31A)–C(32A) 1.52(2), N(33A)–C(32A) 1.39(1); C(1A)‐Au(1A)‐B(31A) 160.1(4), Au(1A)‐B(31A)‐C(32A) 89.0(7), Σ_angles_N(33A) 363(3). b) Measuring the displacement (d) of the coinage metal centre along the B−C bond (r).^[74]^

Complexes **3 a**–**c** can be described as linear d^10^‐metal centres with side‐on coordination of the [B=C]^−^ moiety. The molecular and structural formulae were confirmed by X‐ray diffraction analysis of single crystals grown form Et_2_O solutions (Figure 3 a). The structures of **3 b** and **3 c** contain two molecules in the asymmetric unit, however the metrics are statistically identical accounting for standard deviation errors. The metal‐boron distances in **3 a**–**c** (2.121(2)– 2.23(1) Å) are directly comparable to the side‐on bound diborane complexes of the coinage metals reported by Kinjo and co‐workers (2.096(3)–2.220(4) Å).^[66]^ These values are longer than those found in structurally related σ‐boryl complexes. For example, the metal‐boron distances in [M(IPr)(Bpin)] range from 2.002(3) to 2.063(5) Å (M=Cu, Au; Bpin=pinacolatoborane).[^67^, ^68^] The B=C bond distance for **3 c** (1.52(2) Å) is elongated slightly in comparison to **3 a** (1.469(3) Å) and **3 b** (1.473(5) Å), although the error of these measurements precludes definitive confirmation of this discrepancy. The M−B−C_cAAC_ angle becomes increasingly acute as the group is ascended taking values of 89.0(7)° (**3 c**), 86.0(2)° (**3 b**), and 82.2(1)° (**3 a**). This phenomenon is paired with increased deviation from the ideal linear geometry at the metal, C_NHC_−M−B [°]=160.1(4) (**3 c**), 158.5(1) (**3 b**), 156.4(1) (**3 a**).

These measurements along with the close approach of the metal to the carbenic carbon (2.411(2)–2.68(1) Å) strongly suggest the borataalkene approaches η^2^ coordination. The coordination mode can also be inspected by the displacement of the metal centre along the B−C axis (Figure 3 b). For classical η^2^‐adducts (e.g. symmetric alkene‐metal complexes), the metal is located at the mid‐point along the C=C bond axis (d/r=0.5), whereas an η^1^‐coordination mode (e.g. alkyl‐metal complex) would result in the metal being found outside the C−C bond (d/r<0). The structures of **3 a** and **3 b** show the metal centre is displaced 0.29(5) Å and 0.16(3) Å from boron, respectively. Conversely, in **3 c** the Au atom is only displaced 0.04(3) Å from B, implying the coordination is dominated by the B−Au interaction and an η^1^ coordination mode.

**Figure 4:**
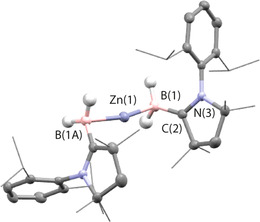
X‐ray diffraction determined structures for **4**. Key structural parameters (distances in Å, angles in °): Zn–B(1) 2.139(2), B(1)–C(2) 1.505(2); B(1)‐Zn(1)‐B(1A) 164.5(1), Zn(1)‐B(1)‐C(1) 97.3.^[74]^

The zinc analogue **4** provides a further benchmark for the η^1^ coordination geometry. As a post transition metal, the filled d‐orbitals of zinc are low in energy and expected to play a very limited role in bonding. The structure of **4** was found have *C*_2_ symmetry about an axis that passes through Zn1 and bisects the B1−Zn1−B1A angle (Figure 4). In the solid‐state, **4** contains a near linear two‐coordinate Zn centre with a B−Zn−B angle of 164.5(1)°. The Zn−B bond length of 2.139(2) Å is consistent with those observed for the group 11 analogues, as is the B=C length of 1.505(2) Å. For comparison, homoleptic two‐coordinate zinc boryl complexes reported by Yamashita and Nozaki contain Zn−B bond lengths ranging from 2.052(3) to 2.087(3) Å.^[69]^ In contrast to the Cu analogue **3 a**, **4** contains a long Zn—C distance (>2.8 Å) which is well beyond the sum of the covalent radii and an open Zn−B−C angle (97.3°).^[70]^ Both metrics are consistent with an η^1^ coordination mode in which the principal interaction between the ligand and metal is through a Zn−B bond. For comparison, related borataalkene complexes in which [CH_2_=B(C_6_F_5_)_2_] is coordinated to titanocene and tantocene have been shown to adopt both η^2^ and η^1^ coordination modes.^[57]^ But in this case the borataalkene coordinates as the η^1^‐C and not η^1^‐B isomer with the M−C interaction dominating the bonding interaction, this difference is likely an effect of the very different steric profiles around the B and C atoms in **A** and [CH_2_=B(C_6_F_5_)_2_].

### Calculations

Theoretical calculations were conducted to gain insight into the bonding in **3 a**–**c** and **4**. DFT calculations were conducted using Gaussian 09. The ωB97x‐D functional with 6‐31G**(C,H,N,B)/SDDAll(M) hybrid‐basis set was applied, with single point PCM(THF) solvent corrections. The computationally optimised structures for **3 a**–**c** and **4** are in good agreement with the experimentally determined crystal structures and were used as inputs for a battery of methods for bonding analysis (QTAIM, NBO, ETS‐NOCV).

**Figure 5:**
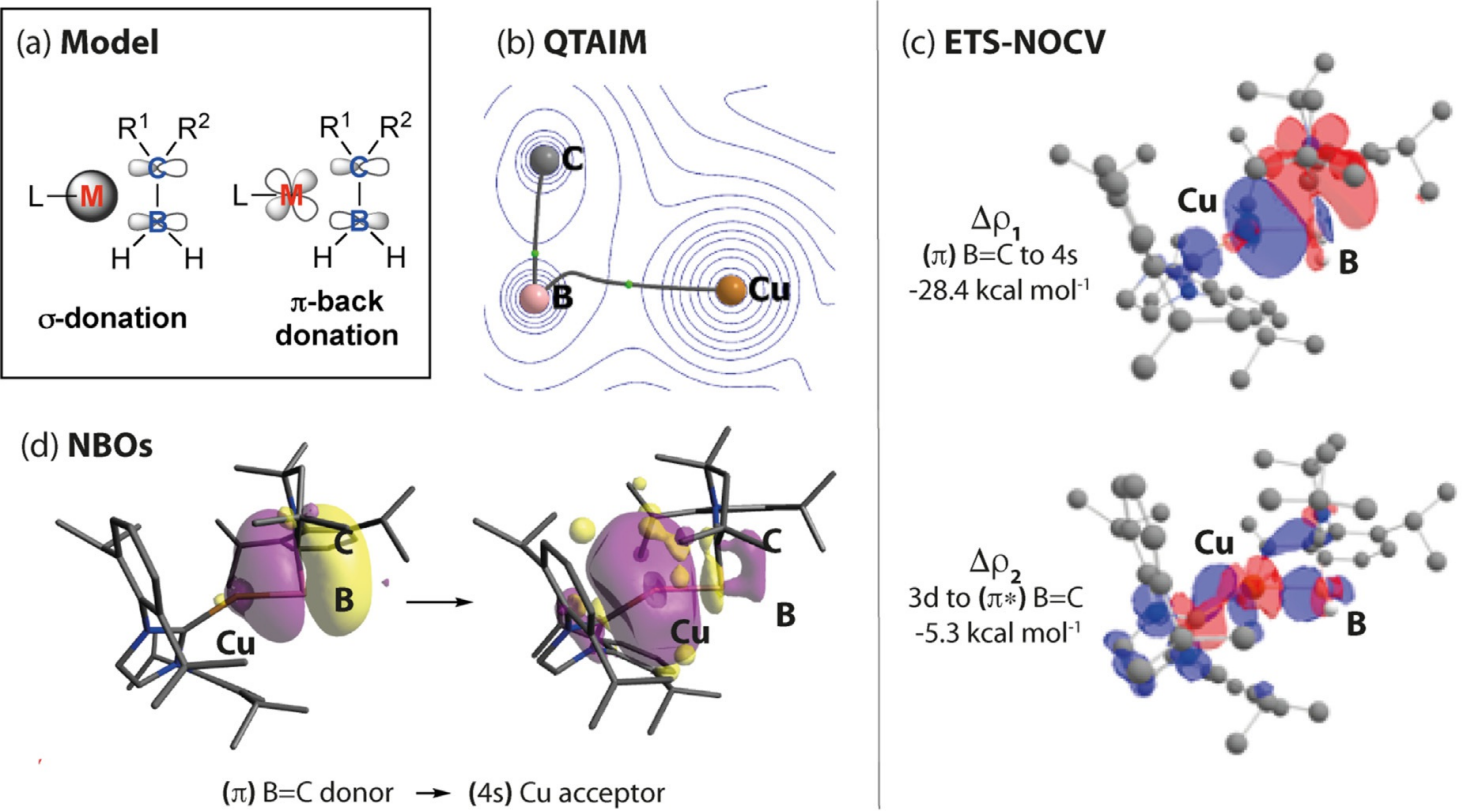
a) Simplified bonding model for **3 a**–**c** and **4**. Data on **3 a** including b) QTAIM, c) ETS‐NOCV and d) NBO calculations.

QTAIM analysis of **3 a**–**c** identifies bond critical points (bcp) between boron and carbon, and boron and the coinage metal, but not from the coinage metal to carbon (Table 1). Nevertheless, the side‐on binding mode and a non‐negligible M–C interaction is supported by the curved bond paths identified in the contour plots from QTAIM analysis (Figure 5 b). The curvature of the bond path is most marked for **3 a** (Cu) and least for **3 c** (Au). Virtually no curvature is seen in the bond paths for **4**. In the free [(cAAC)BH_2_]^−^ anion (**A**), the electron density (*ρ*(**r**)) at the bcp between B and C is found to be 0.20. Upon complexation with a coinage metal, *ρ*(**r**) decreases to 0.19 (Cu, Ag) and 0.18 (Au) implying a reduction in B=C bond order. For **3 a**–**c**, a small *ρ*(r) at the bcp between the M and B (0.06–0.08) is found, consistent with a closed shell ionic interaction. Others have shown that ellipticity is not an accurate metric for evaluating the π‐character of the B=C bond.^[71]^

**Table 1:** QTAIM parameters on **3 a**–**c** and **4**.

	M−B ρ(r)	M−B ∇^2^ρ(r)	B=C ρ(r)	B=C ∇^2^ρ(r)
**A**			0.20	0.23
**3 a**	0.07	0.08	0.19	0.33
**3 b**	0.06	0.04	0.19	0.32
**3 c**	0.08	0.01	0.18	0.34
**4**	0.05, 0.07	0.22, 0.37	0.18, 0.18	0.31, 0.36

A complementary picture emerges from NBO calculations. The WBIs are consistent with B=C multiple bonding character throughout the entire series of compounds, with coordination to the metals reducing the B=C WBI (Table 2). Further, comparison of M−B and M−C WBIs reveals that the covalent component of the metal ligand bonding is dictated by the M−B interaction as is expected for a side‐on but slipped bonding interaction. This M−B value is largest for Au (compared with Ag and Cu) which could be interpreted as an indicator of a stronger binding interaction and a consequence of the greater radial extension of the AOs of Au relative to its lighter congeners.[^72^, ^73^] The M–C interaction in the Zn analogue is the weakest of the series and consistent with very little interaction between the cAAC carbon atom and the metal. Based on the NPA charges, the polarisation of the B=C bond reverses on coordination to the metal, with charge localising on the boron atom and being depleted from the carbon atom in all cases (Table 2). Deviation toward η^1^ coordination (Cu → Ag → Au) is also accompanied by an increased polarisation of the B=C bond toward an ionic B^−^−C^+^ structure as would be expected as the M−B interaction begins to become more important and the M−C interaction (and electron transfer from M to C) is disrupted. At the same time the C−N bond of the cAAC ligand shortens to compensate for the electron deficiency at carbon. The C−N bond length of the cAAC ligand takes values of 1.406(6), 1.413(5), 1.39(1), and 1.350(2) Å for **3 a**, **3 b**, **3 c** and **4** respectively.

**Table 2:** NPA charges and WBIs for complexes **3 a**–**c** and **4**.

	NPA Charges			Wiberg Bond Index		
	M	B	C	M−B	M−C	B=C
**A**		−0.15	−0.32	–	–	1.64
**3 a**	+0.62	−0.47	−0.14	0.25	0.15	1.47
**3 b**	+0.51	−0.44	−0.10	0.29	0.18	1.41
**3 c**	+0.34	−0.53	+0.04	0.43	0.17	1.27
**4**	+1.12	−0.68	+0.07	0.40^[a]^	0.08^[a]^	1.27

[a] data are identical across both ligand systems. Calculated with v 6.0 of NBO.

Inspection of the key orbitals involved in bonding in **3 a**–**c** reveals this interaction conforms to the Dewar–Chatt–Duncanson model (Figure 5 c). Qualitatively the σ‐donation and π‐backdonation components bonding can be inspected by second‐order perturbation analysis. This analysis shows that donation from the π(B=C) bonding orbital to the vacant s(M) metal acceptor orbital is far larger than back‐donation from filled metal d‐orbitals to the π*(B=C). Consistent with this finding, the calculated occupancy of the B=C π‐orbital for **3 a**–**3 c** is lower than **A** due to electron transfer occurring from the borataalkene to the metal (supplementary information, Table S3). Although the data are coherent across two different versions of NBO (v 6.0 and 3.1), the quantitative analysis of the donor‐acceptor interactions by second order perturbation analysis is complicated by the very large and unrealistic energies (100–400 kcal mol^−1^).

We turned to ETS‐NOCV calculations to further support the bonding model and quantify the bonding interactions. Inspection of the Δ*E*_orb_ energies reveals that the orbital (covalent) interaction increases in magnitude across the series **3 c** (118.9 kcal mol^−1^) > **3 b** (71.1 kcal mol^−1^) >**3 a** (58.9 kcal mol^−1^) suggestive of a stronger binding of the [B=C]^−^ moiety to Au over Ag and Cu. The key contributors to Δ*E*_orb_, Δρ_1_ and Δρ_*2*_ involve σ‐donation from the (π) B=C orbital to the metal (n)s orbital (Cu, 4s; Ag, 5s; Au, 6s) and p‐backdonation from the metal (n−1)d orbital to the (π*) B=C orbital. For example, for **3 a**, these σ‐donation and π‐back‐donation components are quantified as contributing 28.4 and 5.3 kcal mol^−1^ to the total Δ*E*_orb_ interaction (Figure 5 d).

To gain a deeper understanding of the potential energy surface that connects η^2^ and η^1^ coordination in **3 a**–**c**, scans of the M−C bond between 1.5–3.4 Å were undertaken with DFT methods. These calculations revealed a flat potential energy surface about the equilibrium bond length. Only one energy minimum was located for each structure. Comparison of the series revealed that this minimum was displaced to longer M−C bond lengths for Cu (2.44 Å), Ag (2.62 Å) and Au (2.76 Å) respectively. The extent of displacement is beyond that expected for solely the difference in covalent radii of the group 11 metals. The calculations support the solid‐state data and suggest that while deformation toward the η^1^ coordination mode of the ligand is energetically accessible for the whole series, it occurs more readily for the heavier analogues.

**Scheme 2:**
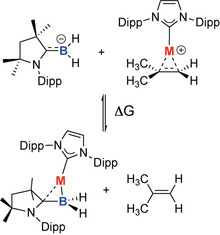
Isodesmic equilibrium between coordination of [(cAAC)BH_2_]^−^ and Me_2_CCH_2_ at [(IPr)M]^+^ (M=Cu, Ag, Au). Thermal parameters calculated at wB97xD//6‐31G**/SDDAll(M) with PCM solvent model (THF).

To assess the strength of binding of **A** to the coinage metal fragments and rationalize the ease of formation of **3 a**–**c** and **4** relative to their alkene analogues, an isodesmic equilibrium was considered (Scheme 2). There is a clear energetic driving force for the formation of the borataalkene complex over the alkene complex (Δ*G*°_rxn_=−42– −52 kcal mol^−1^).

## Conclusion

In summary, we report the preparation and analysis of a complete series borataalkene complexes (**3 a**–**c**) of the coinage metals involving side‐on coordination of a [B=C]^−^ ligand along with an analogous Zn complex (**4**). Calculations confirm that the bonding in these compounds is best described by the Dewar–Chatt–Duncanson model. The structural data and calculations also show the propensity of the ligand to adopt a variety of coordination modes defined by a spectrum in between η^2^ and η^1^ coordination. Deformation toward η^1^ coordination is a phenomenon that is expected to be accompanied by the polarisation of the π‐system of the coordinated ligand and is greatest for Au > Ag > Cu. Due to the isoelectronic relation between [B=C]^−^ and [C=C] moieties, **3 a**–**c** serve as isoelectronic models for catalytically relevant alkene complexes of group 11. Hence, our findings not only demonstrate new coordination chemistry of [B=C]^−^ units they provide unique insight into catalytically relevant isoelectronic alkene analogues.

## Conflict of interest

The authors declare no conflict of interest.

## Supporting information

As a service to our authors and readers, this journal provides supporting information supplied by the authors. Such materials are peer reviewed and may be re‐organized for online delivery, but are not copy‐edited or typeset. Technical support issues arising from supporting information (other than missing files) should be addressed to the authors.

Supplementary

## References

[1] M. A. Bennett , Chem. Rev. 1962, 62, 611–652.

[2] N. Weding , M. Hapke , Chem. Soc. Rev. 2011, 40, 4525–4514. 21594299 10.1039/c0cs00189a

[3] R. Franke , D. Selent , A. Börner , Chem. Rev. 2012, 112, 5675–5732. 22937803 10.1021/cr3001803

[4] Y. Nakajima , S. Shimada , RSC Adv. 2015, 5, 20603–20616.

[5] J. V. Obligacion , P. J. Chirik , Nat. Rev. Chem. 2018, 2, 15–34. 30740530 10.1038/s41570-018-0001-2 PMC6365001

[6] L. Nattmann , R. Saeb , N. Nöthling , J. Cornella , Nat. Catal. 2020, 3, 6–13.

[7] A. S. K. Hashmi , M. Wieteck , I. Braun , M. Rudolph , F. Rominger , Angew. Chem. Int. Ed. 2012, 51, 10633–10637; 10.1002/anie.201204015 22987629

[8] M. Rudolph , A. S. K. Hashmi , Chem. Commun. 2011, 47, 6536–6544. 10.1039/c1cc10780a 21451867

[9] D. Benitez , E. Tkatchouk , A. Z. Gonzalez , W. A. Goddard III , F. D. Toste , Org. Lett. 2009, 11, 4798–4801. 19780543 10.1021/ol9018002 PMC2783583

[10] C. C. Chintawar , A. K. Yadav , N. T. Patil , Angew. Chem. Int. Ed. 2020, 59, 11808–11813; 10.1002/anie.202002141 32203638

[11] X. Hu , D. Martin , M. Melaimi , G. Bertrand , J. Am. Chem. Soc. 2014, 136, 13594–13597. 25222130 10.1021/ja507788r

[12] E. Tkatchouk , N. P. Mankad , D. Benitez , W. A. Goddard III , F. D. Toste , J. Am. Chem. Soc. 2011, 133, 14293–14300. 21861448 10.1021/ja2012627 PMC3168709

[13] C. Brouwer , C. He , Angew. Chem. Int. Ed. 2006, 45, 1744–1747; 10.1002/anie.200504495 16453358

[14] J. Zhang , C.-G. Yang , C. He , J. Am. Chem. Soc. 2006, 128, 1798–1799. 16464072 10.1021/ja053864z

[15] C.-G. Yang , C. He , J. Am. Chem. Soc. 2005, 127, 6966–6967. 15884936 10.1021/ja050392f

[16] Q. Xu , Y. Imamura , M. Fujiwara , Y. Souma , J. Org. Chem. 1997, 62, 1594–1598.

[17] A. S. K. Hashmi , G. J. Hutchings , Angew. Chem. Int. Ed. 2006, 45, 7896–7936; 10.1002/anie.200602454 17131371

[18] H. Pellissier , Chem. Rev. 2016, 116, 14868–14917. 27960274 10.1021/acs.chemrev.6b00639

[19] A. S. K. Hashmi , Chem. Rev. 2007, 107, 3180–3211. 17580975 10.1021/cr000436x

[20] H. Schmidbaur , A. Schier , Organometallics 2010, 29, 2–23.

[21] O. Eisenstein , R. Hoffmann , J. Am. Chem. Soc. 1981, 103, 4308–4320.

[22] D. B. DellAmico , F. Calderazzo , R. Dantona , J. Strähle , H. Weiss , Organometallics 1987, 6, 1207–1210.

[23] A. Fürstner , M. Alcarazo , R. Goddard , C. W. Lehmann , Angew. Chem. Int. Ed. 2008, 47, 3210–3214; 10.1002/anie.200705798 18348113

[24] T. N. Hooper , M. Green , J. E. McGrady , J. R. Patel , C. A. Russell , Chem. Commun. 2009, 45, 3877–3879. 10.1039/b908109g 19662238

[25] T. J. Brown , M. G. Dickens , R. A. Widenhoefer , J. Am. Chem. Soc. 2009, 131, 6350–6351. 19368391 10.1021/ja9015827 PMC2745538

[26] R. A. Sanguramath , T. N. Hooper , C. P. Butts , M. Green , J. E. McGrady , C. A. Russell , Angew. Chem. Int. Ed. 2011, 50, 7592–7595; 10.1002/anie.201102750 21732510

[27] R. E. M. Brooner , R. A. Widenhoefer , Angew. Chem. Int. Ed. 2013, 52, 11714–11724; 10.1002/anie.201303468 24105846

[28] M. A. Celik , C. Dash , V. A. K. Adiraju , A. Das , M. Yousufuddin , G. Frenking , H. V. R. Dias , Inorg. Chem. 2013, 52, 729–742. 23273108 10.1021/ic301869v

[29] C. M. Wyss , B. K. Tate , J. Bacsa , M. Wieliczko , J. P. Sadighi , Polyhedron 2014, 84, 87–95.

[30] A. Zhdanko , M. E. Maier , Angew. Chem. Int. Ed. 2014, 53, 7760–7764; 10.1002/anie.201402557 24923243

[31] M. Sriram , Y. Zhu , A. M. Camp , C. S. Day , A. C. Jones , Organometallics 2014, 33, 4157–4164.

[32] P. Motloch , J. Blahut , I. Cisarova , J. Roithová , J. Organomet. Chem. 2017, 848, 114–117.

[33] Y. Zhu , C. S. Day , A. C. Jones , Organometallics 2012, 31, 7332–7335.

[34] M. Navarro , A. Toledo , S. Mallet Ladeira , E. D. S. Carrizo , K. Miqueu , D. Bourissou , Chem. Sci. 2020, 11, 2750–2758. 34084334 10.1039/c9sc06398f PMC8157524

[35] M. Lee , M. Nguyen , C. Brandt , W. Kaminsky , G. Lalic , Angew. Chem. Int. Ed. 2017, 56, 15703–15707; 10.1002/anie.201709144 29052303

[36] G. Wang , L. Pecher , G. Frenking , H. V. R. Dias , Eur. J. Inorg. Chem. 2018, 4142–4152.

[37] M. W. Rathke , R. Kow , J. Am. Chem. Soc. 1972, 94, 6854–6856.

[38] R. Kow , M. W. Rathke , J. Am. Chem. Soc. 1973, 95, 2715–2716.

[39] J. W. Wilson , J. Organomet. Chem. 1980, 186, 297–300.

[40] A. Berndt , Angew. Chem. Int. Ed. Engl. 1993, 32, 985–1009;

[41] R. A. Bartlett , P. P. Power , Organometallics 1986, 5, 1916–1917.

[42] M. M. Olmstead , P. P. Power , K. J. Weese , J. Am. Chem. Soc. 1987, 109, 2541–2542.

[43] J. D. Hoefelmeyer , S. Solé , F. P. Gabbaï , Dalton Trans. 2004, 54, 1254–1258. 10.1039/b316505a 15252669

[44] J. Möbus , G. Kehr , C. G. Daniliuc , R. Fröhlich , G. Erker , Dalton Trans. 2014, 43, 632–638. 24131986 10.1039/c3dt52373j

[45] S. Kohrt , S. Dachwitz , C. G. Daniliuc , G. Kehra , G. Erker , Dalton Trans. 2015, 44, 21032–21040. 26584629 10.1039/c5dt04058b

[46] P. Moquist , G.-Q. Chen , C. Mück-Lichtenfeld , K. Bussmann , C. G. Daniliuc , G. Kehr , G. Erker , Chem. Sci. 2015, 6, 816–825. 28936322 10.1039/c4sc01711k PMC5592807

[47] S. Helm , H. Noth , Angew. Chem. Int. Ed. Engl. 1988, 27, 1331–1337;

[48] S. Channareddy , G. Linti , H. Noth , Angew. Chem. Int. Ed. Engl. 1990, 29, 199–201;

[49] S. W. Helm , G. Linti , H. Noth , S. Channareddy , P. Hofmann , Chem. Ber. 1992, 125, 73–86.

[50] G. J. Irvine , M. J. G. Lesley , T. B. Marder , N. C. Norman , C. R. Rice , E. G. Robins , W. R. Roper , G. R. Whittell , L. J. Wright , Chem. Rev. 1998, 98, 2685–2722. 11848976 10.1021/cr9500085

[51] J. D. Scollard , D. H. McConville , S. J. Rettig , Organometallics 1997, 16, 1810–1812.

[52] M. G. Thorn , J. S. Vilardo , P. E. Fanwick , I. P. Rothwell , Chem. Commun. 1998, 2427–2428.

[53] K. S. Cook , W. E. Piers , S. J. Rettig , Organometallics 1999, 18, 1575–1577.

[54] S. Zhang , W. E. Piers , X. Gao , M. Parvez , J. Am. Chem. Soc. 2000, 122, 5499–5509.

[55] K. S. Cook , W. E. Piers , T. K. Woo , R. McDonald , Organometallics 2001, 20, 3927–3937.

[56] K. S. Cook , W. E. Piers , P. G. Hayes , M. Parvez , Organometallics 2002, 21, 2422–2425.

[57] K. S. Cook , W. E. Piers , R. McDonald , J. Am. Chem. Soc. 2002, 124, 5411–5418. 11996581 10.1021/ja025547n

[58] K. Watanabe , A. Ueno , X. Tao , K. Škoch , X. Jie , S. Vagin , B. Rieger , C. G. Daniliuc , M. C. Letzel , G. Kehr , et al., Chem. Sci. 2020, 11, 7349–7355. 33209245 10.1039/d0sc02223c PMC7654189

[59] T. A. Bartholome , A. Kaur , D. J. D. Wilson , J. L. Dutton , C. D. Martin , Angew. Chem. Int. Ed. 2020, 59, 11470–11476; 10.1002/anie.202002125 32237193

[60] D. A. Ruiz , G. Ung , M. Melaimi , G. Bertrand , Angew. Chem. Int. Ed. 2013, 52, 7590–7592; 10.1002/anie.201303457 PMC3712875 23765789

[61] M. Arrowsmith , D. Auerhammer , R. Bertermann , H. Braunschweig , M. A. Celik , J. Erdmannsdörfer , I. Krummenacher , T. Kupfer , Angew. Chem. Int. Ed. 2017, 56, 11263–11267; 10.1002/anie.201705561 28640395

[62] M. Arrowsmith , J. Mattock , S. Hagspiel , I. Krummenacher , A. Vargas , H. Braunschweig , Angew. Chem. Int. Ed. 2018, 57, 15272–15275; 10.1002/anie.201809983 30238575

[63] M. C. L. Gerry , W. Lewis-Bevan , A. J. Merer , N. P. C. Westwood , J. Mol. Spectrosc. 1985, 110, 153–163.

[64] S. F. Parker , RSC Adv. 2018, 8, 23875–23880. 10.1039/c8ra04845b PMC9081783 35540255

[65] J. R. Durig , N. E. Lindsay , T. J. Hizer , J. D. Odom , J. Mol. Struct. 1988, 189, 257–277.

[66] W. Lu , R. Kinjo , Chem. Eur. J. 2018, 24, 15656–15662. 30047175 10.1002/chem.201803139

[67] D. S. Laitar , P. Müller , J. P. Sadighi , J. Am. Chem. Soc. 2005, 127, 17196–17197. 16332062 10.1021/ja0566679

[68] C. M. Zinser , F. Nahra , L. Falivene , M. Brill , D. B. Cordes , A. M. Z. Slawin , L. Cavallo , C. S. J. Cazin , S. P. Nolan , Chem. Commun. 2019, 55, 6799–6802. 10.1039/c9cc03171e 31123732

[69] T. Kajiwara , T. Terabayashi , M. Yamashita , K. Nozaki , Angew. Chem. Int. Ed. 2008, 47, 6606–6610; 10.1002/anie.200801728 18637644

[70] P. Pyykkö , M. Atsumi , Chem. Eur. J. 2009, 15, 186–197. 19058281 10.1002/chem.200800987

[71] R. Gupta , E. Rezabal , G. Hasrack , G. Frison , Chem. Eur. J. 2020, 26, 17230–17241. 32780465 10.1002/chem.202001945

[72] P. Pyykkö , Chem. Rev. 1988, 88, 563–594.

[73] D. J. Gorin , F. D. Toste , Nature 2007, 446, 395–403. 17377576 10.1038/nature05592

[74] Deposition Numbers 2020173, 2020174, 2020175, and 2020176 contain the supplementary crystallographic data for this paper. These data are provided free of charge by the joint Cambridge Crystallographic Data Centre and Fachinformationszentrum Karlsruhe Access Structures service www.ccdc.cam.ac.uk/structures.

