# Supplementary material for: Group 11 Borataalkene Complexes: Models for Alkene Activation
Source: Angew Chem Int Ed Engl. 2021 May 3;60(21):12013–9. doi: 10.1002/anie.202100919 (PMC8252388; doi:10.1002/anie.202100919)
Supplement: Supplementary file 1 — Supplementary [file ANIE-60-12013-s001.pdf]

Angewandte Chemie
Eine Zeitschrift der Gesellschaft Deutscher Chemiker

Supporting Information

Group 11 Borataalkene Complexes: Models for Alkene Activation

*Nicholas A. Phillips, Richard Y. Kong, Andrew J. P. White, and Mark R. Crimmin**

anie_202100919_sm_miscellaneous_information.pdf
anie_202100919_sm_miscellaneous_information.cif
anie_202100919_sm_miscellaneous_information.xyz

Contents

1. Experimental Methods ... 3
  1.1 Synthesis of transition-metal boryl complexes ... 4
2. X-ray crystal structures ... 8
  2.1 Displacement of M along the B–C axis ... 11
3. IR Spectra ... 12
4. DFT Methods ... 16
4.2 MOs of $cAACBH_2^-$ (A) and a Polar Alkene ... 17
  4.3 NBO analysis on 3a-c and 4 ... 19
  4.4 QTAIM Analysis ... 21
  Figure S4. QTAIM generated contour plots for the M–B–C_{cAAC} core of 4. ... 23
  Figure S5. QTAIM generated contour plots for 3a showing a network of stabilising non-covalent interactions. ... 23
  4.5 Thermochemistry of B=C⁻ and C=C coordination to Cu, Ag and Au ... 24
  4.6 Potential energy surface for η^2 vs. η^1 coordination of B=C⁻ and C=C ... 24
  4.7 Defining a Bonding Continuum within a Series of Polar and Apolar C=C and B=C⁻ complexes 26
    4.7.1 ETS-NOCV Analysis ... 27
    4.7.2 NBO Data ... 33
  5. Coordinates ... 35
6. References ... 53

NMR spectra located at DOI: *10.14469/hpc/7344*

1. Experimental Methods

All manipulations were carried out using standard Schlenk-line and glovebox techniques under an inert atmosphere of argon or dinitrogen, respectively. A MBraun Labmaster glovebox was employed, operating at <0.1 ppm O_2 and <0.1 ppm H_2O. Solvents were dried over activated alumina from an SPS (solvent purification system) based upon the Grubbs design and degassed before use. Glassware was dried for 12 h at 120 °C prior to use. Benzene-d_6 and toluene-d_8 were stored over 3 Å molecular sieves and distilled prior to use. NMR-scale reactions were conducted in J. Young's tap tubes and prepared in a glovebox. All heating mentioned was done using silicone oil baths. ^{1}H, ^{11}B, and ^{13}C NMR spectra were obtained on BRUKER 400 MHz or 500 MHz machines, unless otherwise stated, and referenced against $SiMe_4$ (^{1}H, ^{13}C) and $Et_2O{\cdot}BF_3$ (^{11}B), all peak intensities are derived against an internal standard peak (^{1}H 1-fluorohexane; δ_H = 4.17 ppm) with values quoted in ppm. NMR data was processed using MestReNova or Topsin software. Multiplicity assignments NMR spectra are labelled as follows: "s" = singlet, "d" = doublet, "t" = triplet, "q" = quartet, "pent" = pentet, "sept" = septet, "br" = broad.

Hydrocarbons were dried over activated 3 Å molecular sieves and freeze-pump-thaw degassed before use. All reagents were acquired from Sigma Aldrich (Merck), Honeywell or Fluorochem and used without further purification unless specified. Where liquids at 25 °C, reagents were dried over activated 3 Å molecular sieves and freeze-pump-thaw degassed prior to use. cAAC^{Me} was synthesised according to the literature procedure and stored in the glovebox.[S1] [{(cAAC^{Me})BH_2}Li(THF)]$_2$ (**1**) was synthesised according to the literature, and stored at -30 °C in the glovebox as a yellow solid.[S2]

Dipp = 2,6-di(*iso*-propyl)phenyl

IPr =

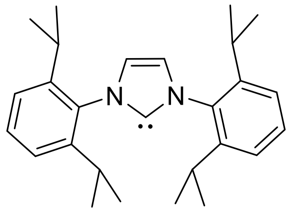

cAAC^{Me} =

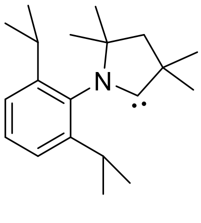

1.1 Synthesis of transition-metal boryl complexes

General procedure for (IPr)MBH$_2$(cAAC^{Me})

In a glovebox, [{(cAAC^{Me})BH$_2$}Li(THF)]$_2$ (100 mg, 0.13 mmol) in Et$_2$O (10 ml) was added dropwise to a stirring suspension of [(IPr)MCl] (0.26 mmol, M = Cu, Ag, Au) in Et$_2$O (5 ml) in an aluminium foil-wrapped vial at 298 K. A colour change from orange to yellow was observed as the reagents mixed. After stirring for 15 min, the solution was filtered and the volatiles removed under vacuum to give a yellow powder (73 - 80%). Recrystallisation from Et$_2$O at -30 °C yielded crystals suitable for X-ray diffraction analysis.

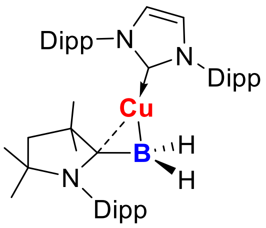

(IPr)CuBH$_2$(cAAC^{Me}) (**3a**)

Yield: 76%, 149 mg, 0.20 mmol.

^{1}H NMR (C$_6$D$_6$, 400 MHz, 298 K): δ$_H$ 7.24 (2H, t, $^3J_{HH}$ = 7.6 Hz, *p*-CH-Dipp IPr), 7.16 (3H, m, CH-Dipp cAAC), 7.10 (4H, d, $^3J_{HH}$ = 7.6 Hz, *m*-CH-Dipp IPr), 6.28 (2H, s, CH-Im), 3.34 (2H, sept, $^3J_{HH}$ = 6.7 Hz, CH-^{i}Pr cAAC), 2.78 (4H, sept, $^3J_{HH}$ = 6.7 Hz, CH-^{i}Pr IPr), 1.80 (2H, s, CH$_2$), 1.39 (12H, d, $^3J_{HH}$ = 6.9 Hz, CH$_3$- ^{i}Pr IPr), 1.39 (6H, s, (C$\underline{H}_3$)$_2$CC$_{cAAC}$), 1.31 (6H, d, $^3J_{HH}$ = 6.9 Hz, CH$_3$- ^{i}Pr cAAC), 1.17 (6H, s, (C$\underline{H}_3$)$_2$CN), 1.14 (6H, d, $^3J_{HH}$ = 6.6 Hz, CH$_3$- ^{i}Pr cAAC), 1.02 (12H, d, $^3J_{HH}$ = 6.9 Hz, CH$_3$- ^{i}Pr IPr).

^{13}C NMR (C$_6$D$_6$, 100 MHz, 298 K): δ$_C$ 183.2 (C$_{IPr}$), C$_{cAAC,}$ *not observed*, 150.5 (NC-Dipp cAAC), 145.2 (NC-Dipp IPr), 140.4 (*o*-C-Dipp cAAC), 136.2 (*o*-C-Dipp IPr), 130.4 (*p*-CH-Dipp IPr), 126.5 (*p*-CH-Dipp cAAC), 124.4 (*m*-CH-Dipp IPr), 123.9 (*m*-CH-Dipp cAAC), 122.6 (CH-Im), 63.7 (CMe$_2$), 57.1 (CH$_2$), 45.1 (CMe$_2$), 36.0 (($\underline{C}$H$_3$)$_2$CC$_{cAAC}$), 29.0 (CH-iPr IPr), 29.0 (($\underline{C}$H$_3$)$_2$CN), 28.7 (CH-iPr cAAC), 27.3 (CH$_3$-^{i}Pr), 24.7 (CH$_3$-^{i}Pr), 24.3 (CH$_3$-^{i}Pr), 23.9 (CH$_3$-^{i}Pr).

^{11}B NMR (C$_6$D$_6$, 128 MHz, 298 K): δ$_B$ -7.4 (br s, BH$_2$).

IR (cm^{-1}): 1358 (vCB), 2345 (v$_{symm}$BH), 2411 (v$_{asymm}$BH).

MS (APCI+) for C$_{47}$H$_{69}$CuBN$_3$: meas. 750.4957 ([M+H]$^+$); calc. 750.4953 ([M+H]$^+$).

Elemental analysis (%) for C$_{47}$H$_{69}$CuBN$_3$: C 74.73, H 9.28, N 5.72 (meas.); C 75.22, H 9.27, N 5.60 (calc.).

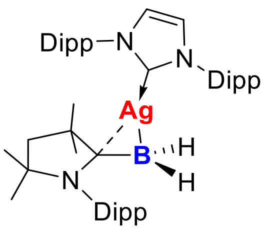

(IPr)AgBH₂(cAAC^{Me}) (**3b**)

Yield: 73%, 151 mg, 0.19 mmol.

^{1}H NMR (C_6D_6, 400 MHz, 298 K): δ_H 7.24 (2H, t, $^3J_{HH}$ = 7.7 Hz, *p*-CH-Dipp IPr), 7.16 (3H, m, CH-Dipp), 7.09 (4H, br s, *m*-CH-Dipp IPr), 6.33 (2H, s, CH-Im), 3.49 (1H, m, CH-^{i}Pr), 3.16 (1H, m, CH-^{i}Pr), 2.67 (4H, m, CH-^{i}Pr), 1.69 (2H, br s, CH_2), 1.40 (9H, br s, CH_3-^{i}Pr), 1.30 (12H, d, $^3J_{HH}$ = 6.9 Hz, CH_3- ^{i}Pr), 1.04 (12H, d, $^3J_{HH}$ = 6.9 Hz, CH_3- ^{i}Pr), 1.04 (6H, d, CH_3- ^{i}Pr), 0.68 (3H, br s, CH_3-^{i}Pr).

^{1}H NMR ($C_6D_5CD_3$, 500 MHz, 223 K): δ_H 7.22 (2H, t, $^3J_{HH}$ = 7.6 Hz, *p*-CH-Dipp IPr), 7.15 (3H, m, CH-Dipp cAAC), 7.04 (2H, d, $^3J_{HH}$ = 7.6 Hz, *m*-CH-Dipp IPr), 6.97 (2H, d, $^3J_{HH}$ = 7.6 Hz, *m*-CH-Dipp IPr), 6.19 (2H, s, CH-Im), 3.56 (1H, sept, $^3J_{HH}$ = 6.7 Hz, CH-^{i}Pr cAAC), 3.15 (1H, sept, $^3J_{HH}$ = 6.7 Hz, CH-^{i}Pr cAAC), 2.68 (2H, sept, $^3J_{HH}$ = 6.7 Hz, CH-^{i}Pr IPr), 2.59 (2H, sept, $^3J_{HH}$ = 6.7 Hz, CH-^{i}Pr IPr), 1.74 (2H, s, CH_2), 1.54 (3H, d, $^3J_{HH}$ = 6.9 Hz, CH_3- ^{i}Pr IPr), 1.47 (6H, d, $^3J_{HH}$ = 6.9 Hz, CH_3- ^{i}Pr IPr), 1.39 (3H, s, $(C\underline{H}_3)_2CC_{cAAC}$), 1.37 (3H, s, $(C\underline{H}_3)_2CC_{cAAC}$), 1.33 (6H, m, CH_3- ^{i}Pr cAAC), 1.08 (6H, s, $(C\underline{H}_3)_2CN$), 1.14 (6H, d, $^3J_{HH}$ = 6.6 Hz, CH_3- ^{i}Pr cAAC), 1.07 (6H, d, $^3J_{HH}$ = 6.9 Hz, CH_3- ^{i}Pr IPr), 1.05 (6H, d, $^3J_{HH}$ = 6.9 Hz, CH_3- ^{i}Pr IPr), 0.61 (3H, d, $^3J_{HH}$ = 6.9 Hz, CH_3- ^{i}Pr IPr).

^{13}C NMR (C_6D_6, 100 MHz, 298 K): δ_C C_{cAAC}, C_{IPr} & NC-Dipp cAAC *not observed*, 145.8 (NC-Dipp IPr), 139.9 (*o*-C-Dipp cAAC), 136.9 (*o*-C-Dipp IPr), 130.5 (*p*-CH-Dipp IPr), 126.6 (*p*-CH-Dipp cAAC), 124.5 (*m*-CH-Dipp IPr), 122.9 (*m*-CH-Dipp cAAC), 122.8 (CH-Im), 64.0 (CMe_2), 57.0 (CH_2), 45.0 (CMe_2), 36.5 ($(\underline{C}H_3)_2CC_{cAAC}$), 36.4 ($(\underline{C}H_3)_2CC_{cAAC}$), 29.0 (CH-iPr IPr), $(\underline{C}H_3)_2CN$ *not observed*, CH-iPr cAAC *not observed*, 24.4 (CH_3-^{i}Pr), 24.3 (CH_3-^{i}Pr).

^{13}C NMR ($C_6D_5CD_3$, 125 MHz, 223 K): δ_C 204.5 (C_{IPr}), $C_{cAAC,}$ *not observed*, 151.0 (NC-Dipp cAAC), 145.3, 145.2 (NC-Dipp IPr), 139.3 (*o*-C-Dipp cAAC), 135.8 (*o*-C-Dipp IPr), 130.3 (*p*-CH-Dipp IPr), 126.4 (*p*-CH-Dipp cAAC), 124.2, 124.2 (*m*-CH-Dipp IPr), 124.0, 123.5 (*m*-CH-Dipp cAAC), 122.5 (CH-Im), 63.7 (CMe_2), 56.1 (CH_2), 44.8 (CMe_2), 36.3, 36.2 ($(\underline{C}H_3)_2CC_{cAAC}$), 29.4 (CH-iPr IPr), 28.7 ($(\underline{C}H_3)_2CN$), 28.2 (CH-iPr cAAC), 27.4, 26.4, 24.6, 24.4, 24.2, 23.8, 23.4 (CH_3-^{i}Pr).

^{11}B NMR (C_6D_6, 128 MHz, 298 K): δ_B -10.3 (br s, BH_2).

IR (cm^{-1}): 1354 (vCB), 2356 (v_{symm}BH), 2411 (v_{asymm}BH).

MS (APCI+) for $C_{47}H_{69}AgBN_3$: meas. 794.4700 ($[M+H]^+$); calc. 794.4708 ($[M+H]^+$).

Elemental analysis (%) for $C_{47}H_{69}AgBN_3$: C 70.08, H 8.57, N 5.33 (meas.); C 71.03, H 8.75, N 5.29 (calc.).

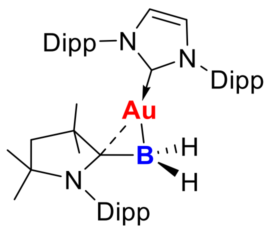

(IPr)AuBH$_2$(cAAC^{Me}) (**3c**)

Yield: 80%, 184 mg, 0.21 mmol.

^{1}H NMR (C_6D_6, 400 MHz, 298 K): δ_H 7.27 (2H, t, $^3J_{HH}$ = 7.7 Hz, CH-Dipp IPr), CH-Dipp cAAC *not observed*, 7.13 (4H, m, CH-Dipp IPr), 6.32 (2H, s, CH-Im), 3.26 (2H, m, CH-^{i}Pr), 2.79 (4H, m, CH-^{i}Pr), 1.79 (2H, br s, CH_2), 1.50 (12H, br s, CH_3-^{i}Pr), ($C\underline{H}_3)_2$C cAAC *not observed*, 1.28 (6H, br m, CH_3-^{i}Pr), 1.07 (12H, d, $^3J_{HH}$ = 6.9 Hz, CH_3-^{i}Pr), 0.92 (6H, m, ($C\underline{H}_3)_2$CN).

^{13}C NMR (C_6D_6, 100 MHz, 298 K): δ_C 204.6 (C_{IPr}), $C_{cAAC,}$ *not observed*, 150.5 (NC-Dipp cAAC), 145.5 (NC-Dipp IPr), 137.8 (C-Dipp cAAC), 135.8 (C-Dipp IPr), 130.0 (*p*-CH-Dipp IPr), 126.8 (*p*-CH-Dipp cAAC), 124.0 (*m*-CH-Dipp IPr), 123.6 (*m*-CH-Dipp cAAC), 122.0 (CH-Im), 66.0 (CMe_2), 54.9 (CH_2), 46.2 (CMe_2), ($C\underline{H}_3)_2$C cAAC *not observed*, 28.6 (CH-iPr), 24.1 (CH_3-^{i}Pr), 23.7 (CH_3-^{i}Pr), 14.0 (CH_3-^{i}Pr).

^{13}C NMR ($C_6D_5CD_3$, 125 MHz, 223 K): δ_C 204.5 (C_{IPr}), $C_{cAAC,}$ *not observed*, 150.8 (NC-Dipp cAAC), 148.6 (NC-Dipp IPr), 146.0, 145.7 (*o*-C-Dipp IPr), 136.2 (*o*-C-Dipp cAAC), 130.6 (*p*-CH-Dipp IPr), 127.5 (*p*-CH-Dipp cAAC), 124.6, 124.5 (*m*-CH-Dipp IPr), 124.3, 124.2 (*m*-CH-Dipp cAAC), 122.5 (CH-Im), 66.5 (CMe_2), 54.8 (CH_2), 46.8 (CMe_2), 37.0 (($\underline{C}H_3)_2CC_{cAAC}$), 34.5 (($\underline{C}H_3)_2CC_{cAAC}$), 31.2, 29.9 (CH-iPr IPr), 29.2 (($\underline{C}H_3)_2$CN), 28.7 (CH-iPr cAAC), 27.6, 27.3, 24.9, 24.8, 24.7, 24.4, 23.4 (CH_3-^{i}Pr).

^{11}B NMR (C_6D_6, 128 MHz, 298 K): δ_B -13.5 (br s, BH_2).

IR (cm^{-1}): 1353 (νCB), 2348 (ν_{symm}BH), 2394 (ν_{asymm}BH).

MS (APCI+) for $C_{47}H_{69}AuBN_3$: meas. 884.5325 ([M+H]$^+$); calc. 884.5323 ([M+H]$^+$).

Elemental analysis (%) for $C_{47}H_{69}AuBN_3$: C 64.82, H 7.68, N 4.86 (meas.); C 63.87, H 7.87, N 4.75 (calc.).

Zn[BH$_2$(cAAC^{Me})]$_2$ (**4**)

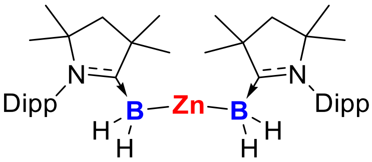

In a glovebox, [{(cAAC^{Me})BH$_2$}Li(THF)]$_2$ (100 mg, 0.13 mmol) in Et$_2$O (10 ml) was added dropwise to a stirring suspension of ZnCl$_2$ (18 mg, 0.13 mmol) in Et$_2$O (5 ml) at 298 K. A colour change from orange to bright yellow was observed as the reagents mixed. After stirring for 15 min, the solution was filtered and volatiles removed under vacuum to give a yellow powder. Recrystallisation from Et$_2$O at -30 °C yielded crystals suitable for X-ray diffraction analysis.

Note: *Product is highly sensitive to light in solution, precipitating metal from solution within ca. 30 mins in direct light.*

Yield: 92%, 80 mg, 0.12 mmol.

^{1}H NMR (C$_6$D$_6$, 500 MHz, 298 K): δ_H 7.17 (2H, m, *p*-CH-Dipp cAAC), 7.09 (4H, d, $^3J_{HH}$ = 7.6 Hz, *m*-CH-Dipp cAAC), 3.04 (4H, sept, $^3J_{HH}$ = 6.7 Hz, CH-^{i}Pr cAAC), 1.71 (4H, s, CH$_2$), 1.62 (12H, s, (C$\underline{H}_3$)$_2$CC$_{cAAC}$), 1.48 (12H, br d, $^3J_{HH}$ = 6.8 Hz, CH$_3$-^{i}Pr cAAC), 1.23 (12H, d, $^3J_{HH}$ = 6.7 Hz, CH$_3$-^{i}Pr cAAC), 1.01 (12H, s, (C$\underline{H}_3$)$_2$CN).

^{13}C NMR (C$_6$D$_6$, 125 MHz, 298 K): δ_C C$_{cAAC,}$ *not observed*, 148.1 (NC-Dipp cAAC), 136.2 (*o*-C-Dipp cAAC), 128.4 (*p*-CH-Dipp cAAC), 124.7 (*m*-CH-Dipp cAAC), 69.0 (CMe$_2$), 53.1 (CH$_2$), 48.1 (CMe$_2$), 33.9 (($\underline{C}$H$_3$)$_2$CC$_{cAAC}$), 29.2 (($\underline{C}$H$_3$)$_2$CN), 29.2 (CH-iPr cAAC), 27.6 (br s, CH$_3$-^{i}Pr), 23.8 (CH$_3$-^{i}Pr).

^{11}B NMR (C$_6$D$_6$, 128 MHz, 298 K): δ_B -22.6 (br s, BH$_2$).

IR (cm^{-1}): 1293 (vCB), 2373 (v$_{symm}$BH), 2420 (v$_{asymm}$BH).

MS (APCI+) for C$_{40}$H$_{66}$ZnBN$_2$: meas. 597.5123 ([M-Zn+H]$^+$); calc. 597.5490 ([M-Zn+H]$^+$). Apparent decomposition during ionisation to known decomposition product resulting from reductive elimination:

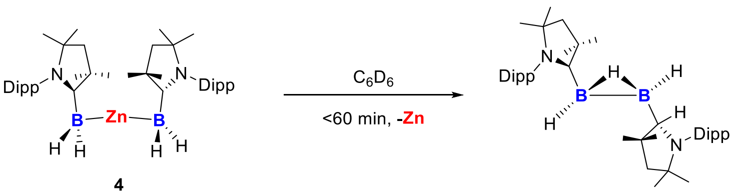

Elemental analysis (%) for C$_{40}$H$_{66}$ZnBN$_2$: multiple attempts but data unreliable due to decomposition of the sample.

2. X-ray crystal structures

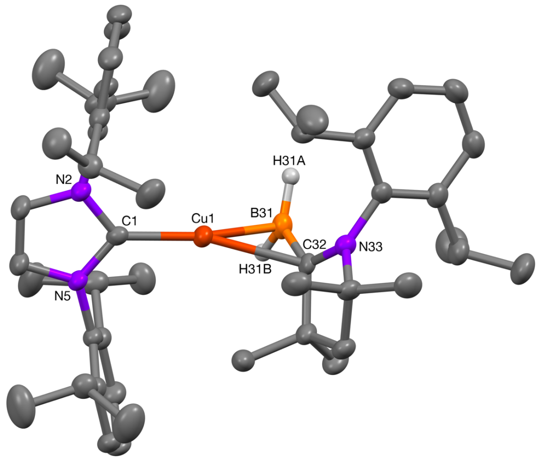

The crystal structure of **3a** (50% probability ellipsoids).

Crystal data for **3a**: $C_{47}H_{69}BCuN_3 \cdot C_4H_{10}O$, *M* = 824.52, triclinic, *P*-1 (no. 2), *a* = 10.8538(5), *b* = 12.2921(5), *c* = 20.0596(11) Å, α = 75.591(4), β = 75.709(5), γ = 75.986(4)°, *V* = 2464.9(2) Å^3, *Z* = 2, D_c = 1.111 g cm^{-3}, μ(Cu-Kα) = 0.890 mm^{-1}, *T* = 173 K, yellow blocks, Agilent Xcalibur PX Ultra A diffractometer; 9373 independent measured reflections (R_{int} = 0.0411), F^2 refinement,[S3] R_1(obs) = 0.0433, wR_2(all) = 0.1184, 7090 independent observed absorption-corrected reflections [$|F_o| > 4\sigma(|F_o|)$, completeness to θ_{full}(67.7°) = 98.3%], 540 parameters. CCDC 2020173.

The two B31–H hydrogen atoms in the structure of **3a** were both located from Δ*F* maps and refined freely subject to a B–H distance constraint of 1.12 Å.

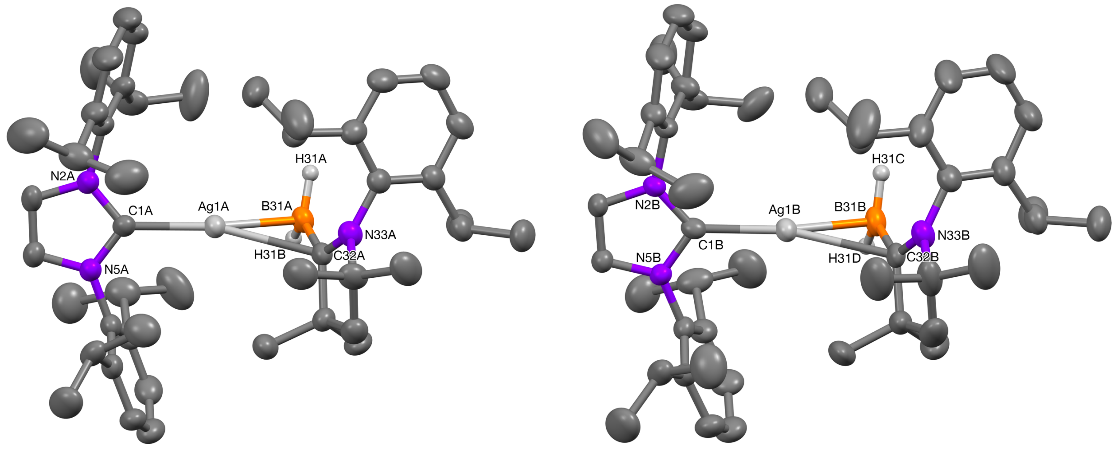

The structures of the two independent complexes present in the crystal of **3b** (50% probability ellipsoids).

Crystal data for **3b**: $C_{47}H_{69}AgBN_3 \cdot 0.75(C_4H_{10}O)$, M = 850.32, triclinic, P-1 (no. 2), a = 12.5083(3), b = 17.5755(7), c = 22.7768(8) Å, α = 81.420(3), β = 88.640(2), γ = 85.769(3)°, V = 4937.3(3) Å^3, Z = 4 [two independent complexes], D_c = 1.144 g cm^{-3}, μ(Cu-Kα) = 3.527 mm^{-1}, T = 173 K, pale yellow tablets, Agilent Xcalibur PX Ultra A diffractometer; 18767 independent measured reflections (R_{int} = 0.0506), F^2 refinement,[S3] R_1(obs) = 0.0443, wR_2(all) = 0.1173, 13137 independent observed absorption-corrected reflections [$|F_o| > 4\sigma(|F_o|)$, completeness to θ_{full}(67.7°) = 98.4%], 1103 parameters. CCDC 2020174.

The structure of **3b** was found to contain two independent complexes (**3b-A** and **3b-B**) in the asymmetric unit. The C15-based isopropyl group in complex **3b-B** was found to be disordered. Two orientations were identified of *ca*. 67 and 33% occupancy, their geometries were optimised, the thermal parameters of adjacent atoms were restrained to be similar, and only the non-hydrogen atoms of the major occupancy orientation were refined anisotropically (those of the minor occupancy orientation were refined isotropically). The included diethylether solvent was found to be disordered. For the O60-based molecule two orientations were identified of *ca*. 74 and 26% occupancy, whilst for the O70-based molecule, which is adjacent to a centre of symmetry, two unique orientations were identified of *ca*. 41 and 9% occupancy. For each site the geometries of the appropriate orientations were optimised, the thermal parameters of adjacent atoms were restrained to be similar, and only the non-hydrogen atoms of the major occupancy orientations were refined anisotropically (those of the minor occupancy orientations were refined isotropically). The two B31–H hydrogen atoms in each complex were located from ΔF maps and refined freely subject to a B–H distance constraint of 1.12 Å.

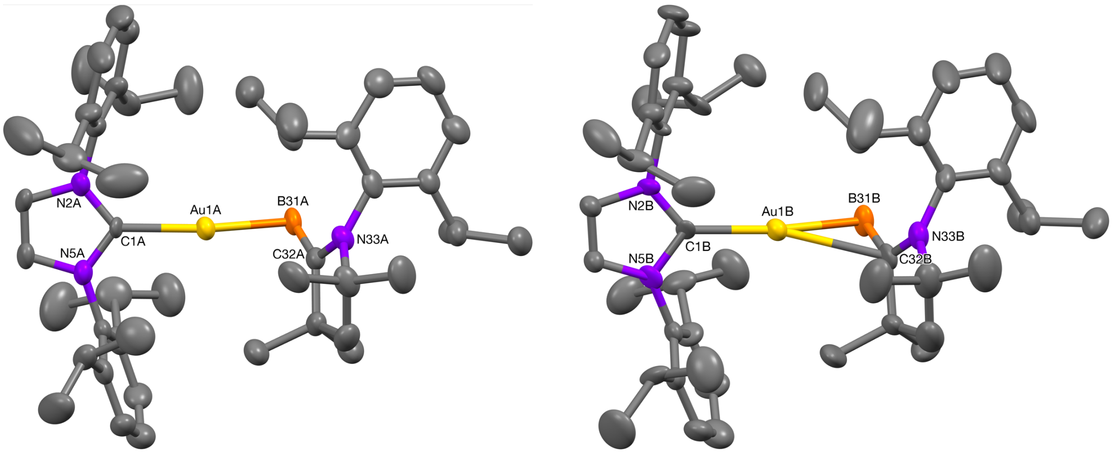

The structures of the two independent complexes present in the crystal of **3c** (50% probability ellipsoids).

Crystal data for **3c**: $C_{47}H_{69}AuBN_3{\cdot}1.25(C_4H_{10}O)$, *M* = 976.47, triclinic, *P*-1 (no. 2), *a* = 12.4822(9), *b* = 17.5391(7), *c* = 22.7593(17) Å, α = 81.618(5), β = 88.437(6), γ = 85.794(5)°, *V* = 4915.4(6) Å^3, *Z* = 4 [two independent complexes], D_c = 1.319 g cm^{-3}, μ(Mo-Kα) = 3.032 mm^{-1}, *T* = 173 K, yellow blocks, Agilent Xcalibur 3 E diffractometer; 19345 independent measured reflections (R_{int} = 0.0434), F^2 refinement,[S3] R_1(obs) = 0.0756, wR_2(all) = 0.1813, 12359 independent observed absorption-corrected reflections [$|F_o| > 4\sigma(|F_o|)$, completeness to θ_{full}(25.2°) = 98.3%], 980 parameters. CCDC 2020175.

Reciprocal space analysis of the data set for the structure of **3c** clearly showed the crystal to be badly twinned, with the initial indexing using only *ca*. 58% of the observed spots. Unfortunately, despite numerous efforts, attempts to model this twinning at the data processing stage proved unsatisfactory, with the best results coming from the standard, non-twin, data processing.

The structure of **3c** was found to contain two independent complexes (**3c-A** and **3c-B**) in the asymmetric unit. The C15-based isopropyl group in complex **3c-B** was found to be disordered. Two orientations were identified of *ca*. 51 and 49% occupancy, their geometries were optimised, the thermal parameters of adjacent atoms were restrained to be similar, and only the non-hydrogen atoms of the major occupancy orientation were refined anisotropically (those of the minor occupancy orientation were refined isotropically). The presumed two B31–H hydrogen atoms in each complex could not be located from Δ*F* maps and so were omitted, leaving the atom list for the asymmetric unit low by 4H.

The included solvent was found to be highly disordered, and the best approach to handling this diffuse electron density was found to be the SQUEEZE routine of PLATON.[S4] This suggested a total of 211 electrons per unit cell, equivalent to 52.8 electrons per asymmetric unit. Before the use of SQUEEZE the solvent clearly resembled diethylether ($C_4H_{10}O$, 42 electrons), and 1.25 dichloromethane molecules corresponds to 52.5 electrons, so this was used as the solvent present. As a result, and combined with the four “missing” B–H hydrogen atoms, the atom list for the asymmetric unit is low by 2 x 1.25($C_4H_{10}O$) + H_4 = $C_{10}H_{29}O_{2.5}$ (and that for the unit cell low by $C_{20}H_{58}O_5$) compared to what is actually presumed to be present.

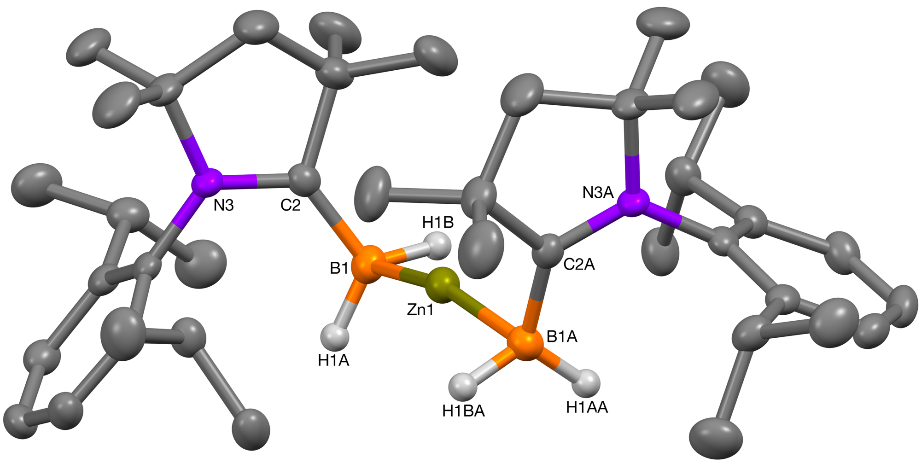

The crystal structure of the C_2-symmetric complex **4** (50% probability ellipsoids).

Crystal data for **4**: $C_{40}H_{66}B_2N_2Zn$, M = 661.93, monoclinic, $C2/c$ (no. 15), a = 29.1705(10), b = 7.8039(3), c = 17.8909(7) Å, β = 107.304(4)°, V = 3888.4(3) Å^3, Z = 2 [C2 symmetry], D_c = 1.131 g cm^{-3}, μ(Mo-Kα) = 0.660 mm^{-1}, T = 173 K, yellow blocks, Agilent Xcalibur 3 E diffractometer; 3925 independent measured reflections (R_{int} = 0.0193), F^2 refinement,[S3] R_1(obs) = 0.0335, wR_2(all) = 0.0823, 3433 independent observed absorption-corrected reflections [$|F_o| > 4\sigma(|F_o|)$, completeness to θ_{full}(25.2°) = 98.8%], 220 parameters. CCDC 2020176.

The structure of **4** was found have C_2 symmetry about an axis that passes through Zn1 and bisects the B1–Zn1–B1A angle. The two B1–H hydrogen atoms were both located from ΔF maps and refined freely subject to a B–H distance constraint of 1.12 Å.

2.1 Displacement of M along the B–C axis

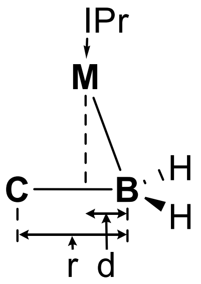

	Displacement along B-C (d, Å)	%Displacement (d/r)
**3a**	0.29(5)	0.20(4)
**3b (A)**	0.16(3)	0.11(2)
**3b (B)**	0.18(3)	0.12(3)
**3c (A)**	0.04(3)	0.026(3)
**3c (B)**	0.12(4)	0.08(1)

Table S1. Measuring the displacement (d) of the coinage metal centre along the B–C bond (r) for all independent molecules in the unit cell of the X-ray crystal structure.

3. IR Spectra

3a

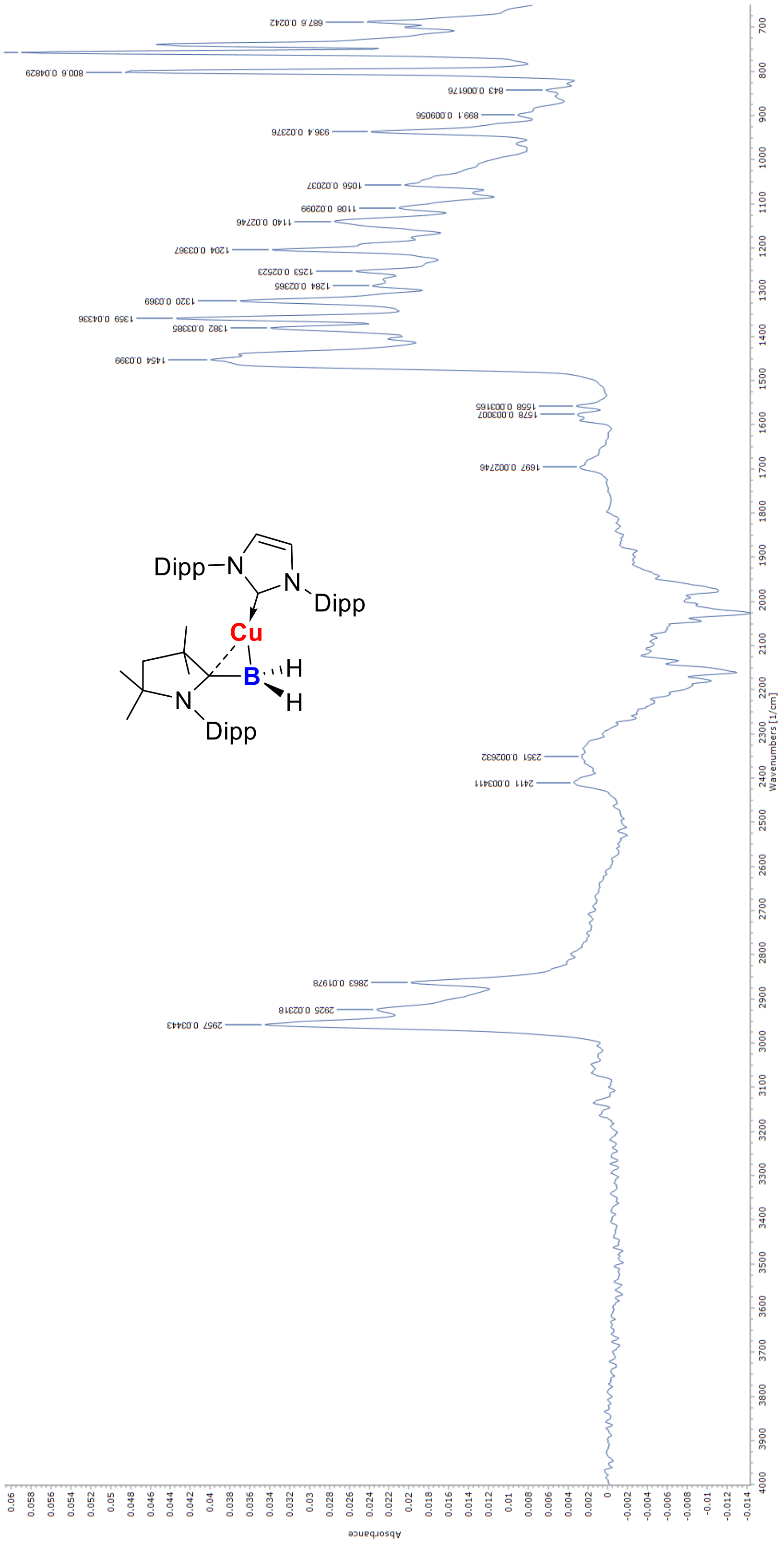

3b

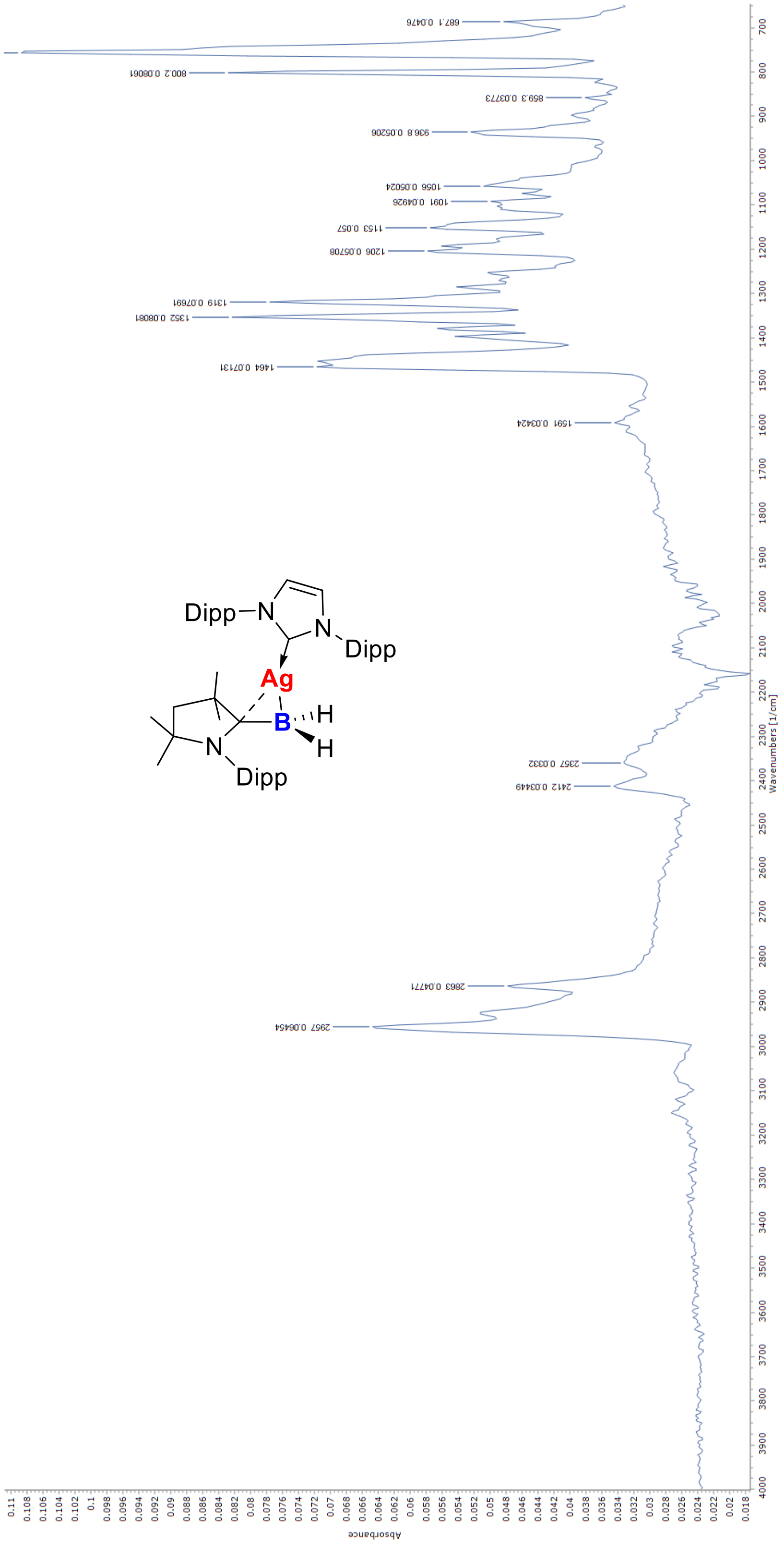

687.1 0.0476
800.2 0.08061
859.3 0.03773
936.8 0.05206
1056 0.05024
1091 0.04926
1153 0.057
1206 0.05708
1319 0.07691
1352 0.08081
1464 0.07131
1591 0.03424
2357 0.0332
2412 0.03449
2863 0.04771
2957 0.06454
Wavenumbers [1/cm]
Absorbance
Dipp
N
Ag
B
H

3c

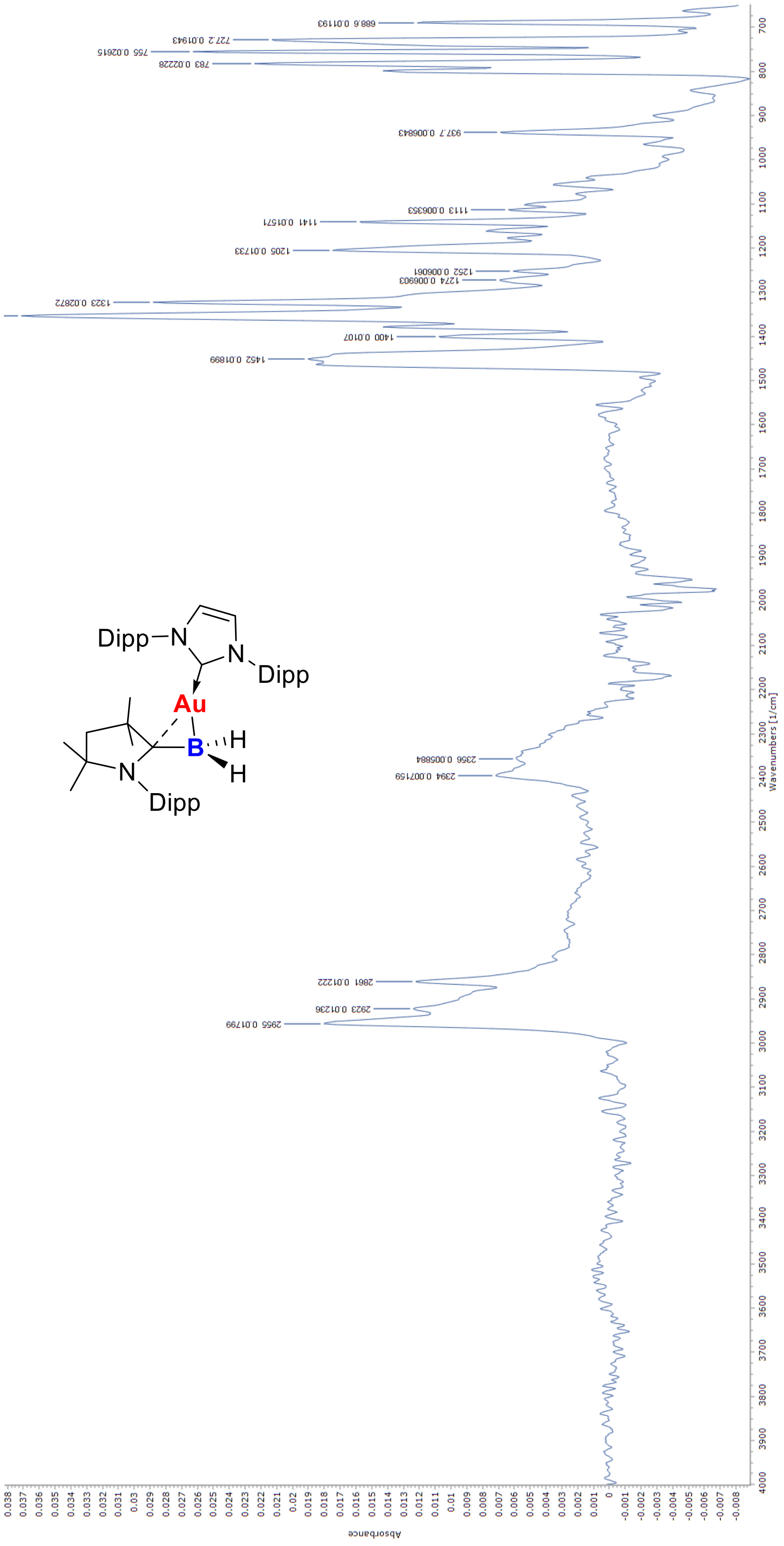

4

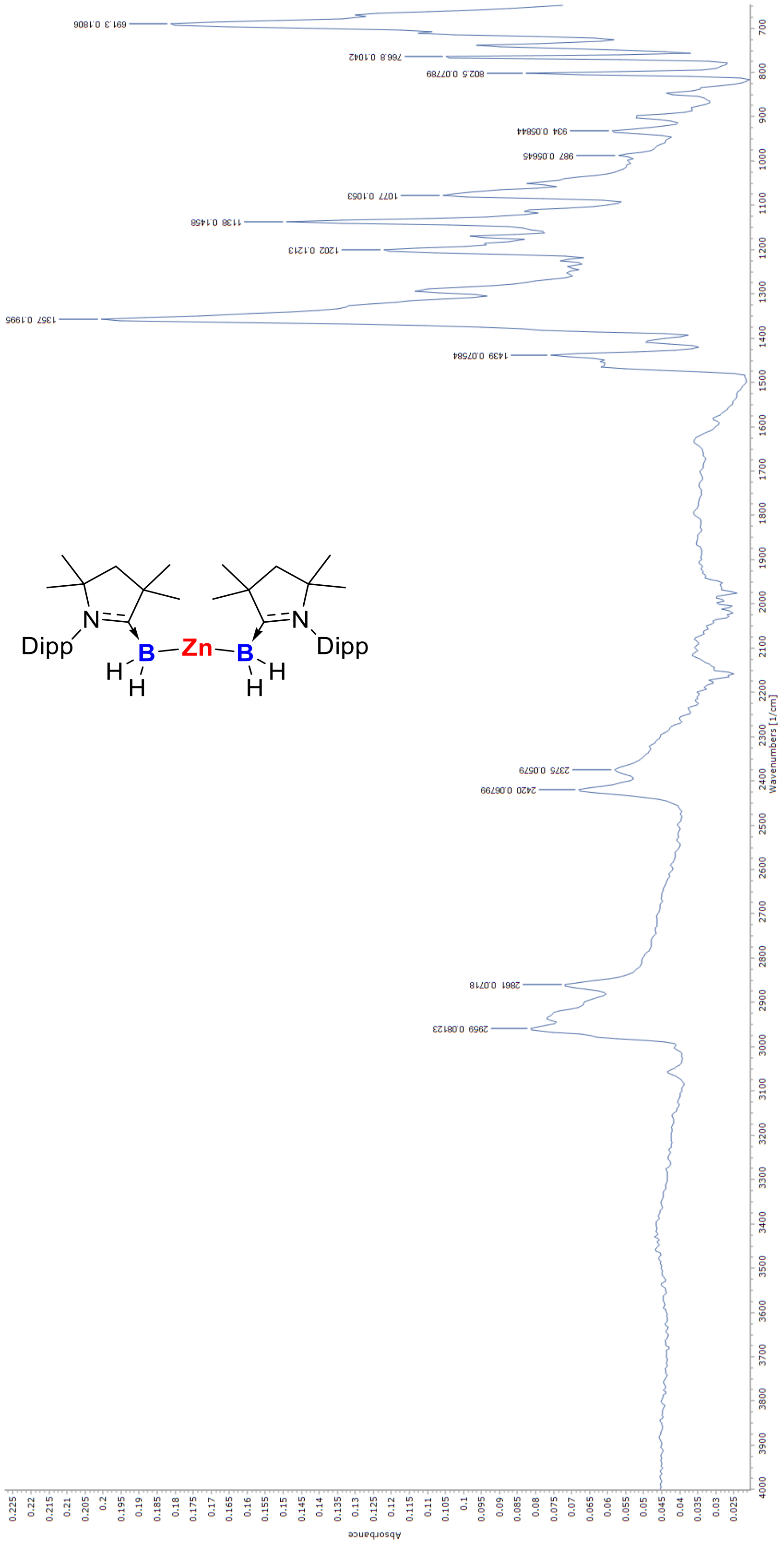
691.3 0.1806
766.8 0.1042
802.5 0.07789
934 0.05844
987 0.05645
1077 0.1053
1138 0.1458
1202 0.1213
1357 0.1995
1439 0.07584
2375 0.0579
2420 0.06799
2861 0.0718
2959 0.08123
Wavenumbers [1/cm]
Absorbance
Dipp
N
B
Zn
H

4. DFT Methods

The geometries of complexes were optimised using density functional theory (DFT) as implemented in the Gaussian 09 program package. [S5] The 6-31G(d,p) basis-set was used for non-metal atoms (C, H, N, O, B, P), and SDDAll was applied to metal atoms (Cu, Ag, Au, Zn).[S6, S7] Optimisations were performed using the range-separated hybrid functional ωB97x with Grimme's D2 correction (wB97XD)[S8] and an ultrafine integration (99,950) grid (int=ultrafine). Stationary points were characterised depending on their imaginary frequencies (0 for minima and 1 for TSs). NBO analysis was performed using the NBO 6.0 and NBO 3.1 programs as implemented in Gaussian 09 at the same level of theory.[S9]

Corrections were made for solvation in THF (ε = 7.58) using the polarizable continuum model.[S10] These corrections were applied after the optimisation procedures as single point corrections.

ETS-NOCV[S11] calculations were performed using DFT as implemented in Orca 4.2.1.[S12,13] Optimised geometries from the Gaussian 09 calculations detailed above were used. Single-point calculations were performed using the ωB97x range-separated hybrid functional[S14] with Grimme's D3 dispersion correction[S15] applied (ωB97x-D3), and an ultrafine (99,950) grid (grid6). The def2-tzvpp basis set was used for non-metal atoms (C, H, N, O, B, P) and the associated effective core potential (def2-ECP) was applied to metal atoms (Cu, Ag, Au, Zn).[S16]

QTAIM calculations were performed using the AIMAll software.[S17, S18]

4.1 Comparison of Calculated Structures with X-ray Data

	**3a**		**3b**			**3c**			**4**	
	Xray	DFT	Xray(A)	Xray(B)	DFT	Xray(A)	Xray(B)	DFT	Xray	DFT
B=C_{caac}	1.469(3)	1.48	1.473(5)	1.469(5)	1.49	1.524(16)	1.490(16)	1.51	1.505(2)	1.51
M–B	2.121(2)	2.10	2.288(4)	2.283(4)	2.29	2.234(12)	2.241(12)	2.22	2.139(2)	2.13
M–C	2.411(2)	2.43	2.633(4)	2.618(3)	2.62	2.682(10)	2.625(10)	2.76		2.76
M–C_{NHC}	1.944(2)	1.96	2.152(4)	2.149(4)	2.16	2.088(9)	2.088(9)	2.09	–	–
M–B–C	82.2(1)	83.4	86.0(2)	85.6(2)	85.1	89.0(7)	87.0(7)	93.2	100.3(1)	97.3
B–M–C_{NHC}	156.39(8)	149.3	158.5(1)	157.5(1)	157.4	160.1(4)	159.3(4)	163.4	–	–

Table S1. Comparison of key metrics from DFT calculations (ωB97xD) with solid state structures for **3a-c** and **4**. For **3b** and **3c** two molecules are present in the unit cell and data is included for both.

4.2 MOs of cAACBH$_2^-$ (A) and a Polar Alkene

Analysis of the Kohn-Sham MOs of **A** are consistent with the existing of B=C bonding motif and a π-interaction between B and C. This finding is further supported by NBO calculations (*vide infra*). Key orbitals are represented in Figure S1.

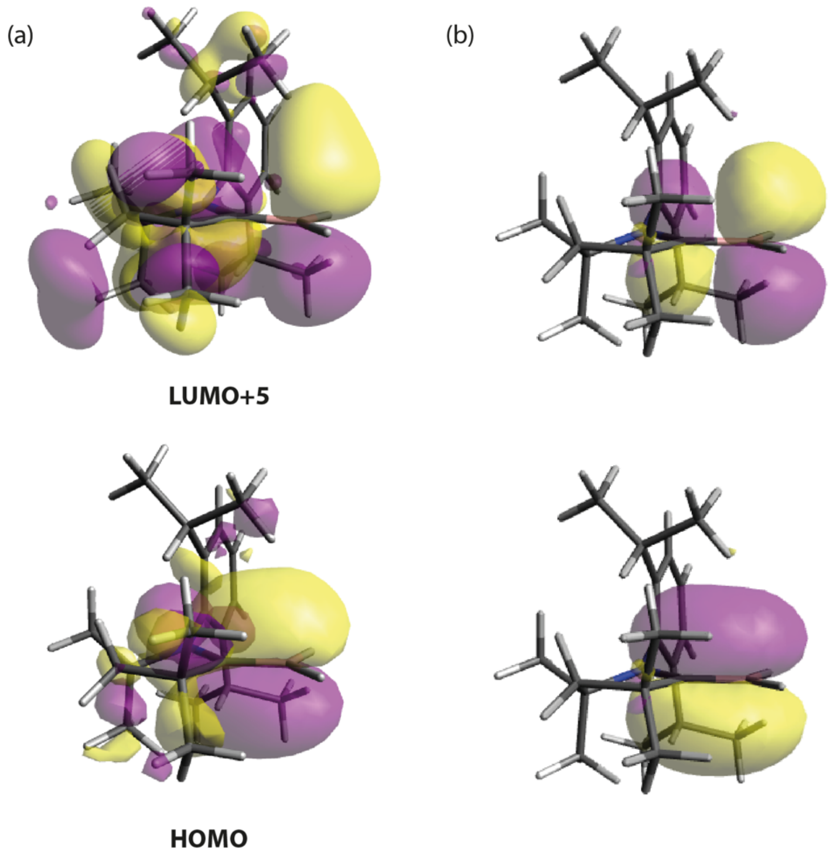

Figure S1. (a) HOMO and LUMO+5 Kohn-Sham MOs of **A**. (b) corresponding NBOs.

The energy gap between the HOMO and LUMO+5 is +9.15 eV. Based on the electronegativity differences it can be expected there should be a larger coefficient on C in the bonding MO and larger coefficient on B on the anti-bonding MO. This is borne out qualitatively in the representations of the orbitals (and in the NPA charge analysis – Table S2). It should be noted however that the contributions to the NBOs are relatively even: 66% pAO carbon ; 34% pAO boron (bonding NBO) and 34% pAO carbon ; 66% pAO boron (anti-bonding NBO).

An analogy can be made to a polar alkene and the related MOs for $CH_3O(CH_3)C{=}CH_2$ are given below for comparison (Figure S2).

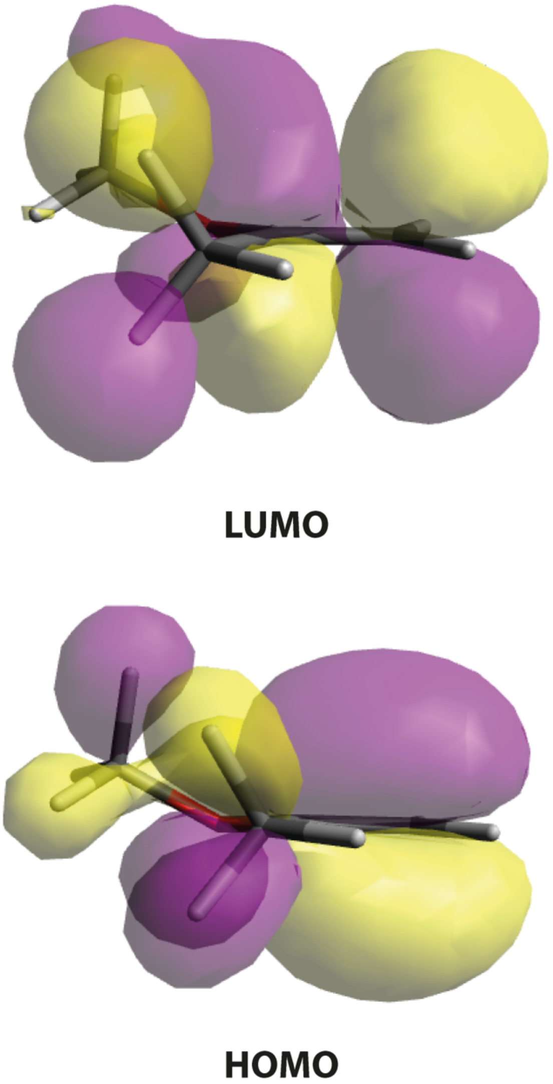

Figure S2. HOMO and LUMO Kohn-Sham MOs of $CH_3O(CH_3)C{=}CH_2$.

4.3 NBO analysis on 3a-c and 4

	2nd Order Perturbation (kcal mol^{-1})		Wiberg Bond Index			NPA charges		
	π(B=C) → M(s)	M(d) → π*(B=C)	M–B	M–C	B=C	M	B	C
**A**	-	-	-	-	1.64		–0.15	–0.32
**3a**	111	9	0.25	0.15	1.47	+0.62	–0.47	–0.14
**3b**	134	8	0.29	0.18	1.41	+0.51	–0.44	–0.10
**3c**	374	14	0.43	0.17	1.27	+0.34	–0.53	+0.04
**4**	274[b]	10[b]	0.40[a]	0.08[a]	1.27	+1.12	–0.68	+0.07

Table S2. Selected data from NBO (v 6.0) calculations on **3a-c** and **4**. [a]data are identical across both ligand systems. [b]values for one Zn–BH_2 system based on partitioning of fragments in NBO calculation.

Wiberg Bond Indices: The WBIs are consistent with B=C multiple bonding character throughout the entire series of compounds. The value above 1 is consistent with a high degree of covalency in the B=C bond and a bond order somewhere between 1 and 2. These values can be compared against those calculated for a series of cationic borenium species of the form $[L{=}BH_2]^+$ (L = carbene) which have been formulated to contain a discrete C=B π-bond.[S19] For example, borenium species in which L is both a σ-donor and π-donor have been calculated to have B=C WBIs of 0.84 – 1.42 and 0.84 – 1.39 with B3LYP/TZVP and M06/6-311G(d,p) methods respectively (NBO v 6.0). Consistent with the bonding model, coordination to group 11 or 12 metals reduces the B=C WBI. Further, comparison of M–B and M–C WBIs reveals that the covalent component of the metal ligand bonding dominating by the metal–boron interaction as is expected for a side-on but slipped bonding interaction. This M–B value is largest for Au (compared with Ag and Cu) as would be expected based on the radial extension of the orbitals increasing down the triad. The M---C interaction in the Zn analogue is the weakest of the series consistent with very little interaction between the cAAC carbon atom and the metal.

NPA Charges: Based on the NPA charges, the polarisation of the B=C bond reverses on coordination with negative charge localising on the boron atom and being depleted from the carbon atom in all cases. This finding is again consistent with a dominate M–B interaction in the binding mode. Slippage from the η^2 toward the η^1 bonding mode (Cu → Ag → Au → Zn) is accompanied by an increased polarisation of the B=C bond as B^-–C^+ as would be expected as the σ-boryl interaction begins to dominate and the M---C interaction (and electron transfer from M to C) is disrupted.

Second Order Perturbation: The data from second order perturbation theory should be treated with a caution. This method is known to be most useful when the interaction energies are small. In the current case values above >100 kcal mol^{-1} and beyond a reasonable estimate for the true interaction energies. Nevertheless, the qualitative observation of key donor-acceptor acceptor interactions along with the relative values for the σ-donation and π-backdonation components are consistent with the bonding model presented and supported by QTAIM and ETS-NOCV calculations (*vide infra*). For the NBO (v

6.0) calculation of **4** the calculation partitions the molecule to recognise the donor-acceptor interaction between Zn and only one B=C unit of the two ligands, the other is dominated by an interaction between Zn and the B atom.

	NBO occupancy & atom orbital contributions					
	B–C (σ)		B=C (π)		M (acceptor)	
	occ.	*atomic orbitals*	*occ.*	*atomic orbitals*	*occ.*	*atomic orbitals*
**3a**	1.97	32% B($sp^{2.3}$)	1.76	46% B(p)	0.45	Cu{s(95%)d(5%)}
		68% C($sp^{1.3}$)		54% C(p)		
**3b**	1.97	31% B($sp^{2.3}$)	1.69	47% B(p)	0.54	Ag{s(94%)d(6%)}
		69% C($sp^{1.2}$)		53% C(p)		
**3c**	1.97	31%B ($sp^{2.6}$)	1.56	54% B(p)	0.77	Au{s(86%)d(13%)}
		69% C($sp^{1.2}$)		46% C(p)		
**4**	1.97	32%B ($sp^{2.5}$)	1.60	59% B(p)	0.85	Zn{s(98%)d(2%)}
		68% C($sp^{1.2}$)		41% C(p)		

Table S3. Calculated NBOs (v 6.0) for the metal-ligand interaction, detailing orbital occupancy and contribution from the constituent atom orbitals.

Recently it has been argued by Frenking and coworkers that a further limitation of modern methods for NBO calculations (including v 6.0) is that they build in an assumption in which the vacant (n)p valence orbitals of transition metals are not considered as genuine valence orbitals. Calculations on $ENi(CO)_3^-$ (E = Li–Cs) have shown the limitations of this assumption as different versions of NBO (v 6.0 *vs* 3.1) can capture quite different bonding descriptions with the neglect of the (n)p-AOs of Ni leading to unrealistic WBIs and NPA charges.[S20] Based on this finding we have also calculated **3a-c** and **4** with NBO v 3.1. Key data are given in Table S4 and are consistent with those calculated with NBO v6.0.

	**2nd Order Perturbation (kcal mol^{-1})**		**Wiberg Bond Index**			**NPA charges**		
	π(B=C) → M(s)	M(d) → π*(B=C)	M–B	M–C	B=C	M	B	C
**A**	-	-	-	-	1.64	-	–0.15	–0.31
**3a**	98.0	7.9	0.29	0.17	1.46	+0.42	–0.45	–0.13
**3b**	117.5	7.7	0.32	0.19	1.41	+0.34	–0.42	–0.09
**3c**	-[a]	-[a]	0.45	0.18	1.27	+0.17	–0.51	+0.05
**4**	94.1[b]	8.8[b]	0.45	0.10	1.26	+0.90	–0.65	+0.08

Table S4. Selected data from NBO (v 3.1) calculations on **3a-c** and **4**. [a] Based on the partitioning of the molecule in the NBO calculations these data are not available. [b] values for one Zn–BH_2 system based on partitioning of fragments in NBO calculation.

4.4 QTAIM Analysis

QTAIM analysis of the bonding identifies bond critical points (bcp) between boron and carbon, and boron and the coinage metal, but not from the coinage metal to carbon. Nevertheless, the side-on binding mode and a non-negligible C---M interaction is supported by the curved bond paths identified in the contour plots from QTAIM analysis (Figure S1). The curvature of the bond path is most marked for **3a** (Cu) and least for **3c** (Au).

In the free $[(cAAC)BH_2]^-$ anion (**A**), the electron density at the B–C bcp ($\rho(\mathbf{r})$) is found to be 0.20, the lower limit for a covalent interaction. Upon complexation with a coinage metal, $\rho(\mathbf{r})$ decreases to 0.19 (Cu, Ag) and 0.18 (Au) implying a reduction in B=C bond order. Interestingly, the Laplacian of $\rho(\mathbf{r})$ ($\nabla^2\rho(\mathbf{r})$) at the bcp between B and C reveals depletion of electrons for all compounds. This is least pronounced in **A** ($\nabla^2\rho(\mathbf{r})$ = 0.23) and increases for the coinage metal complexes (0.32 – 0.34).

For the metal complexes **3a-c**, a small ρ(r) at the bcp between the M and B (0.06 – 0.08) is found, consistent with a closed shell interaction ionic interaction between. Supporting this, the Laplacian of $\rho(\mathbf{r})$ reveals a concentration of electrons similar to a hydrogen bond at the bcp between B and M for all compounds in the series. This appears to become more covalent across the series as $\nabla^2\rho(\mathbf{r})$ decreases (Cu: 0.08, Ag: 0.04, Au: 0.01).

		**A**	**3a**	**3b**	**3c**
**$\rho(\mathbf{r})$**	TM–B	-	0.07	0.06	0.08
	B–C	0.20	0.19	0.19	0.18
**$\nabla^2\rho(\mathbf{r})$**	TM–B	-	0.08	0.04	0.01
	B–C	0.23	0.33	0.32	0.34
**Ellipticity (ε)**	TM–B	-	0.61	0.53	0.16
	B–C	0.38	0.24	0.18	0.18
**H(r)**	TM–B	-	-0.02	-0.02	-0.04
	B–C	-0.19	-0.17	-0.17	-0.16

Table S5. QTAIM parameters on **A** and **3a-c**.

The ellipticity (ε) is found to be 0.38 suggesting deviation from the cylindrical symmetry of a σ-bond. For comparison, ε = 0.45 for the C=C bond in ethylene. Upon complexation to a coinage metal, ε for B=C is found to decrease to 0.18 – 0.24. While these data could be interpreted as supporting the observation of π-character within the B=C bond, it is worth noting that a recent study comparing computational methods for modelling the electronic structure of B=C bonds concluded that ε_{bcp} is potentially a misleading descriptor for the p-character of the bond – instead capturing the influence of the B–H bonds that are found in close proximity to the bcp between B and C.[S17]

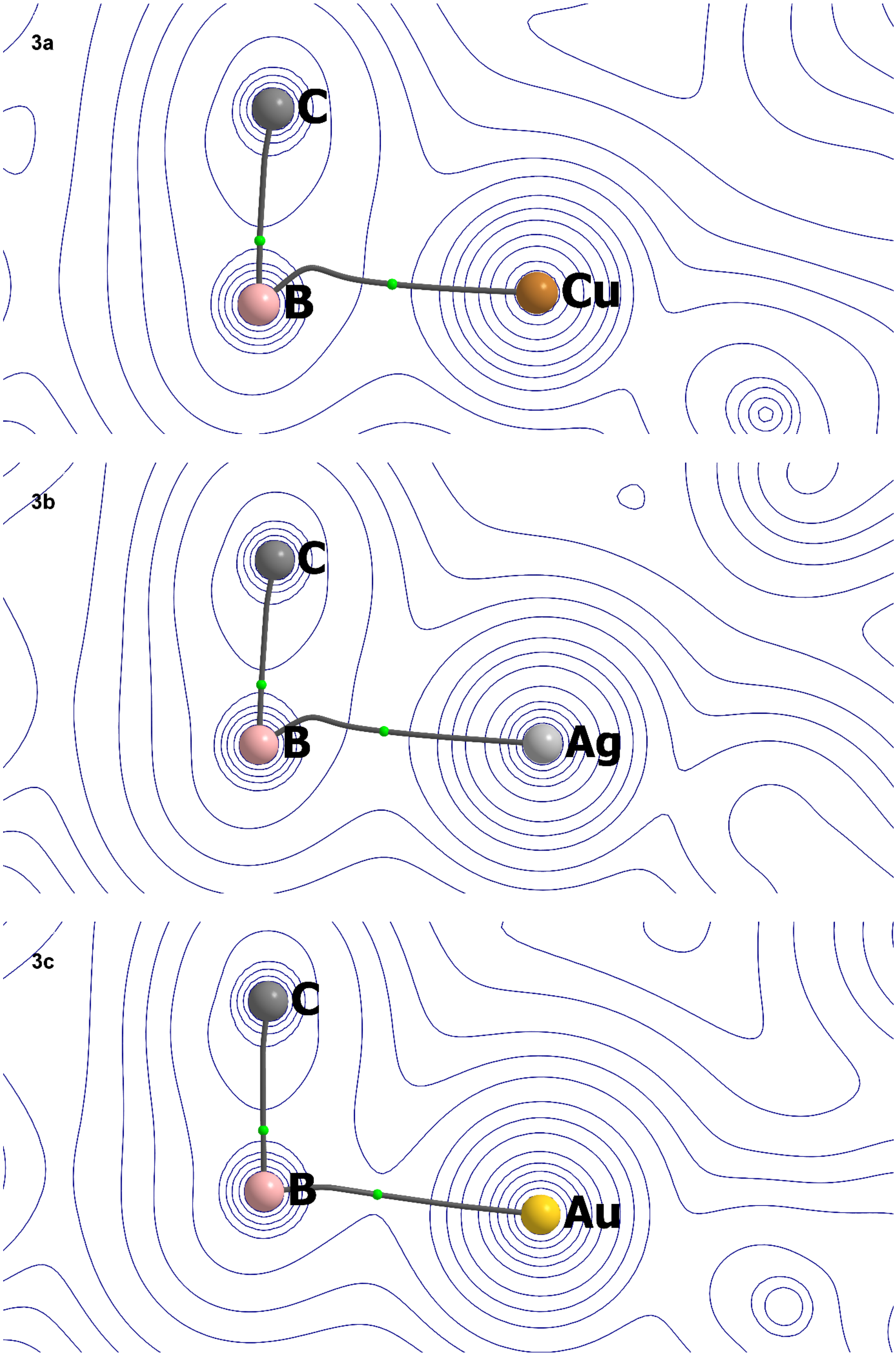

Figure S3. QTAIM generated contour plots for the M–B–C_{cAAC} core of **3a**, **3b** and **3c**.

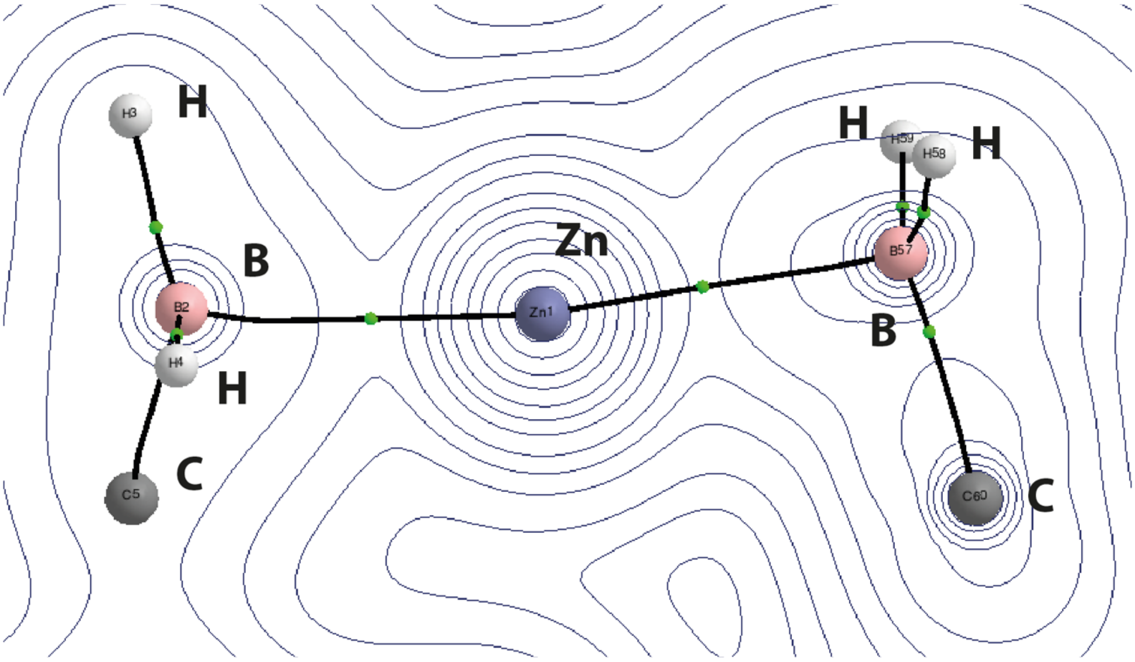

Figure S4. QTAIM generated contour plots for the M–B–C_{cAAC} core of **4**.

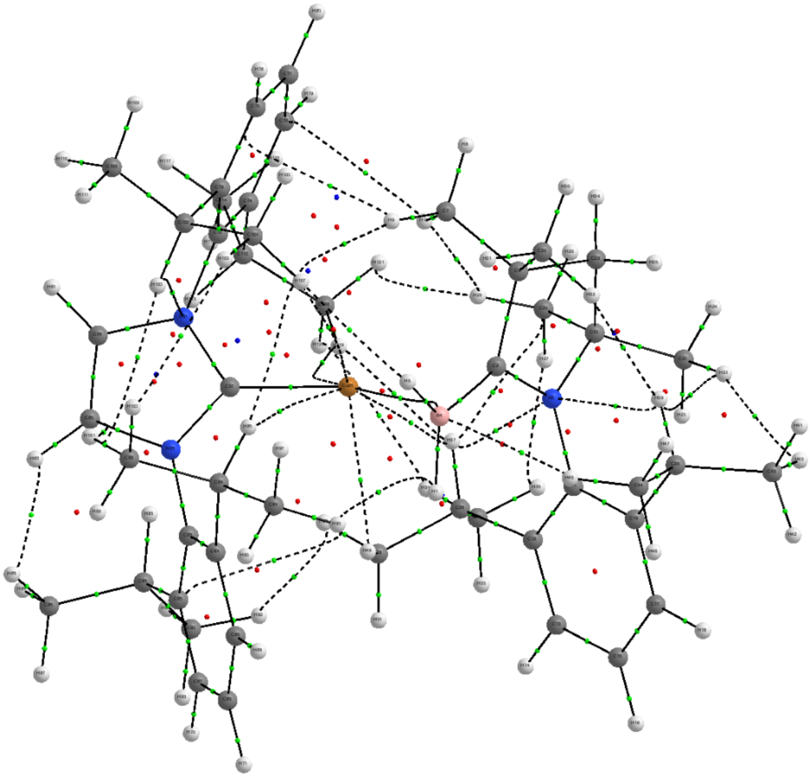

Figure S5. QTAIM generated contour plots for **3a** showing a network of stabilising non-covalent interactions.

4.5 Thermochemistry of B=C⁻ and C=C coordination to Cu, Ag and Au

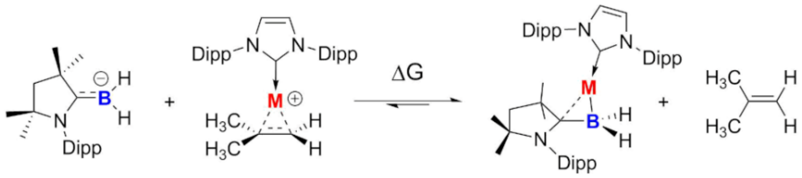

M	ΔG (kcal mol^{-1})
**Cu**	-42
**Ag**	-48
**Au**	-52

Scheme S1. Isodesmic equilibrium between coordination of [(cAAC)BH$_2$]$^-$ and Me$_2$CCH$_2$ at [(IPr)M]$^+$ (M = Cu, Ag, Au). Thermal parameters calculated at wB97xD//6-31G**/SDDAll(M) with PCM solvent model (THF).

4.6 Potential energy surface for η^2 vs. η^1 coordination of B=C⁻ and C=C

For the complexes [(IPr)M][L] (M = Cu, Ag, Au; L = [(cAAC)BH$_2$]$^-$, Me$_2$C=CH$_2$), the M···C distance was scanned between 2.2 – 2.8 Å to assess the energetic cost of deviation from the observed side-on coordination modes. [NB. Sum of covalent radii (Å) for r(M+C) = 1.87 (Cu), 2.03 (Ag), 1.99 (Au).s[S21]

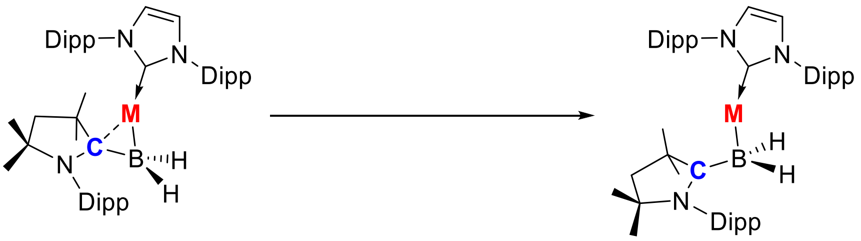

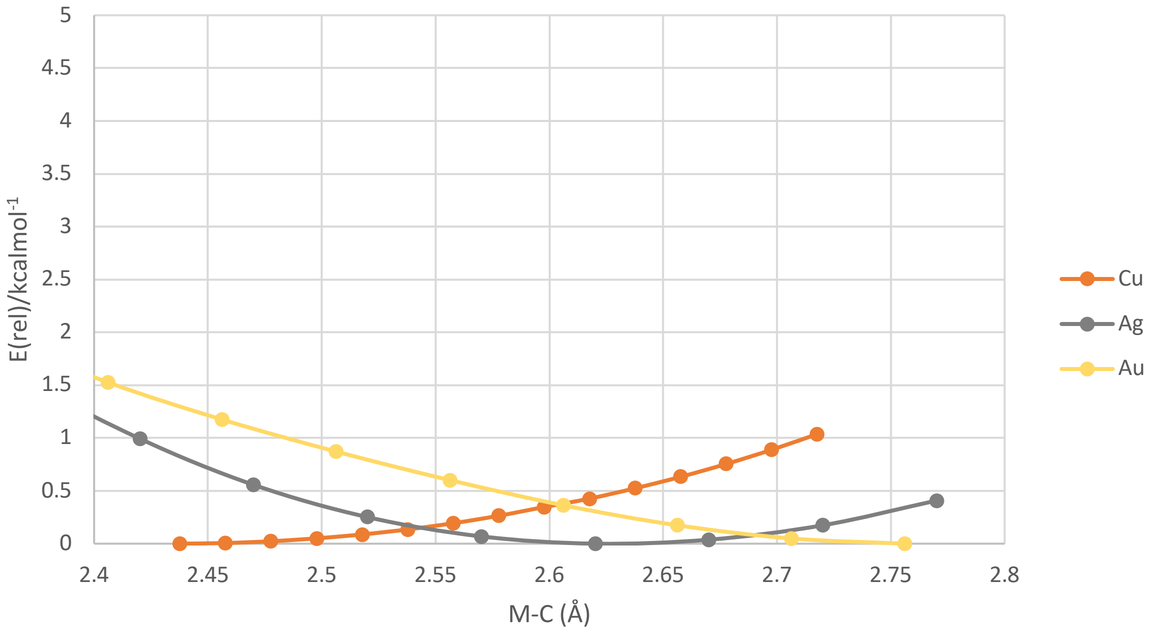

Figure S6. Calculated energy scan for the variation in M···C interaction across the series [(IPr)M][(cAAC)BH$_2$], M = Cu (**3a**), Ag (**3b**), Au (**3c**).

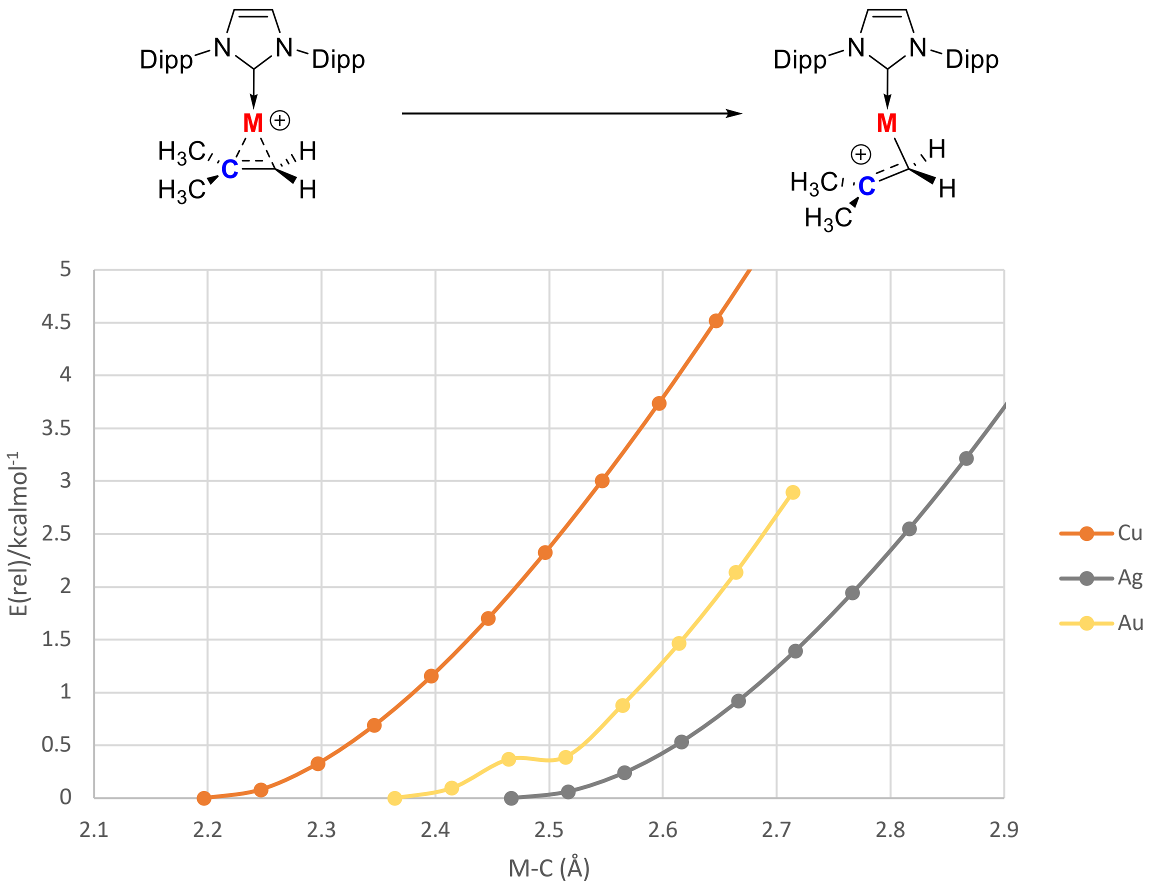

Figure S7. Calculated energy scan for the variation in M···C interaction across the series $[(IPr)M][Me_2C{=}CH_2]^+$, M = Cu, Ag, Au.

The calculated scans show that for L = $[(cAAC)BH_2]^-$ (Figure S6) the energy cost of distortion and "slippage" of the metal centre along the B–C axis is small (*ca*. 0.4 kcal mol^{-1} per 0.1 Å). This is likely due to the ability of the adjacent N(p) orbital which can stabilise the relative build-up of positive charge, akin to the free carbene. Whereas, for L = $Me_2C{=}CH_2$ (Figure S7) distortion to a more "end-on" coordination mode has a higher, but still accessible, associated energy cost (*ca*. 1.0 – 0.8 $kcalmol^{-1}$ per 0.1 Å).

4.7 Defining a Bonding Continuum within a Series of Polar and Apolar C=C and B=C⁻ complexes

Based on these data we attempted to gain further insight into the bonding between C=C and B=C⁻ units to group 11 metals. The approach that was taken was to construct a self-consistent series of complexes which ranged from apolar C=C bonds to polar C=C bonds, to B=C⁻ bonds, to an isolated BH_2^- fragment (Figure S4). This approach was constructed to interrogate the role of polarisation of the substrate in slippage and the corresponding effect on the electronic structure. As part of this series we also considered the deletion of the cAAC moiety along with the replacement of this component with a weaker acceptor PH_3 (σ* P–H) in order to provide a benchmark for related σ-boryl complexes which are either incapable or less capable of accepting electron density from the metal and hence not likely to adopt a side-on bonding interaction.

Both NBO and ETS-NOCV analysis has been conducted on this series and the bonding picture that emerges further supports the (NBO, QTAIM) data presented above for **3a-c**. A consistent bonding picture emerges that defines a continuum between a true σ-boryl complex in which bonding is exclusively through a M–B interaction, through to slipped side-on π-complexes to symmetrically bound π-complexes. Compounds **3a-c** sit on this continuum and can be confidentially assigned as slipped side-on π-complexes with a small but appreciable M---C interaction due to the presence of the acceptor orbital on the carbene carbon.

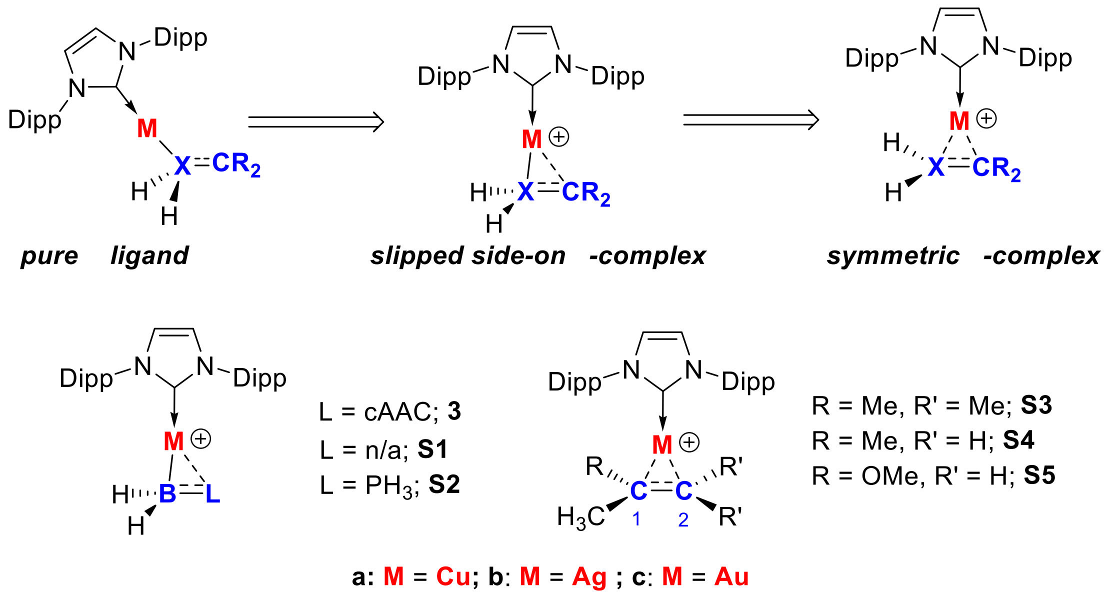

Figure S8. (a) Definition of a continuum between pure σ-boryl complex and side on X=C interactions (X = C, or B⁻). (b) A series of compounds considered to probe this continuum.

4.7.1 ETS-NOCV Analysis

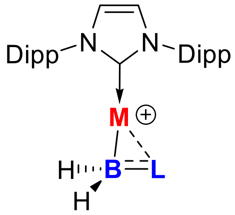

3: L = cAAC
a: **M** = **Cu**;
b: **M** = **Ag**;
c: **M** = **Au**

**Complex**<br>ΔE_{orb}	$\Delta\rho_1$	$\Delta\rho_2$
3a<br>–58.9 kcal mol^{-1}	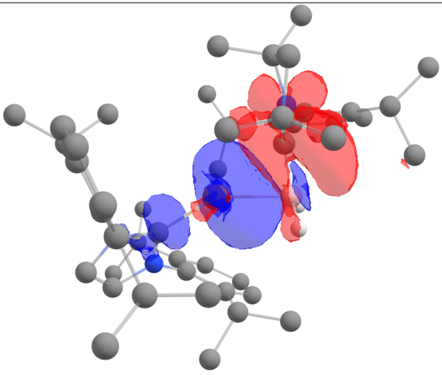<br>σ-(B=C to Cu(4s))<br>-28.4 kcal mol^{-1}	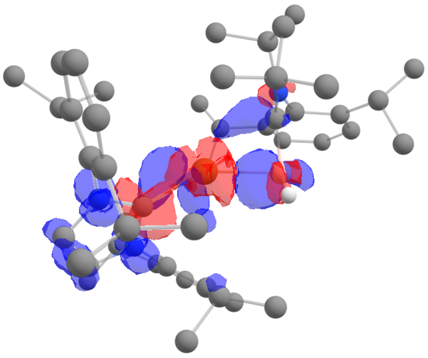<br>π-(Cu(3d) to B=C*)<br>-5.3 kcal mol^{-1}
**3b**<br>-71.1 kcal mol^{-1}	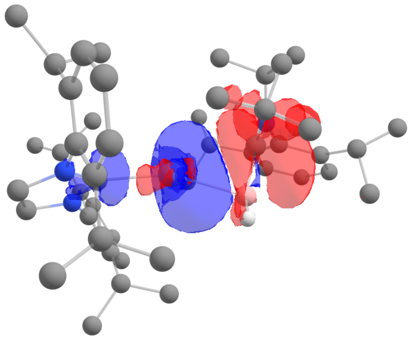<br>σ-(B=C to Ag(5s))<br>-43.6 kcal mol^{-1}	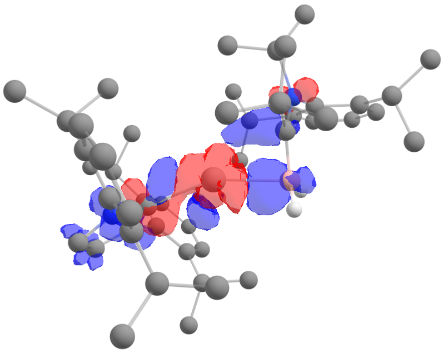<br>π-(Ag(4d) to B=C*)<br>-4.7 kcal mol^{-1}
**3c**<br>–118.9 kcal mol^{-1}	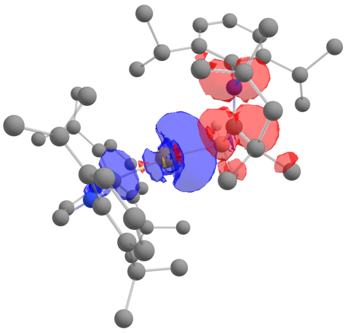<br>σ-(B=C to Au(6s))<br>-88.7 kcal mol^{-1}	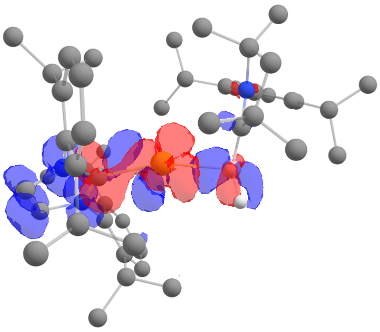<br>π-(Au(5d) to B=C*)<br>-5.3 kcal mol^{-1}

Table S6: Selected deformation density data on complexes **3a-c**. Charge flow from red to blue.

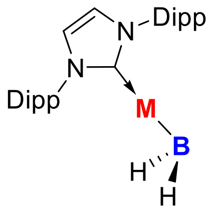

S1 a: M = Cu;
b: M = Ag;
c: M = Au

**Complex**<br>ΔE_{orb}	$\Delta\rho_1$	$\Delta\rho_2$
**S1*a***<br>–55.1 kcal mol^{-1}	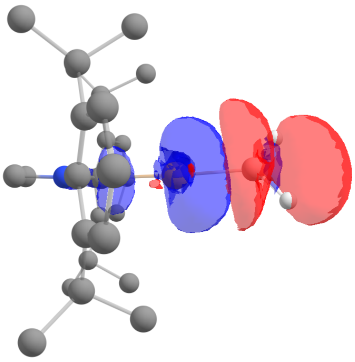<br>σ-(B(sp^2) to Cu(4s))<br>-28.9 kcal mol^{-1}	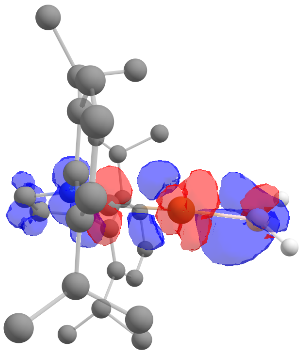<br>π-(Cu(3d) to B(2p))<br>-4.6 kcal mol^{-1}
**S1b**<br>–97.2 kcal mol^{-1}	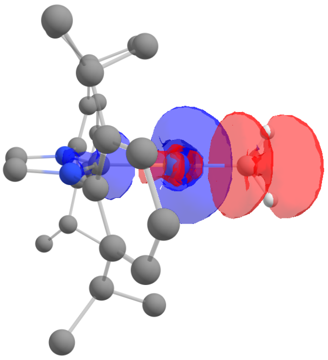<br>:<br>σ-(B(sp^2) to Ag(5s))<br>-69.2 kcal mol^{-1}	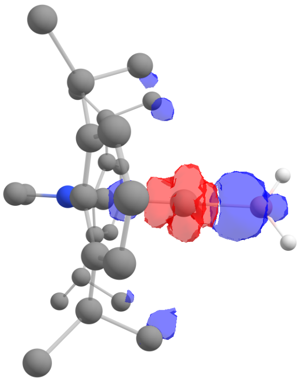<br>σ-(Ag(4dz^2) to B(sp^2))<br>-4.7 kcal mol^{-1}
**S1c**<br>– kcal mol^{-1}	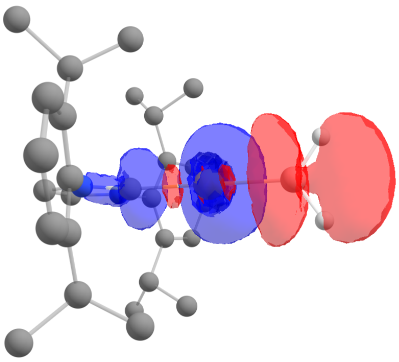<br>σ-(B(sp^2) to Au(6s))<br>-88.7 kcal mol^{-1}	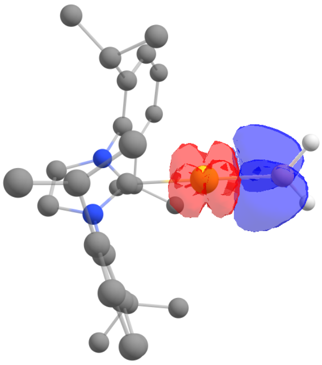<br>π-(Au(5d) to B(2p))<br>-5.3 kcal mol^{-1}

Table S7: Selected deformation density data on complexes **S1a-c**. Charge flow from red to blue.

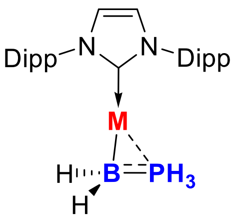

S2 a: **M** = **Cu**;
b: **M** = **Ag**;
c: **M** = **Au**

**Complex** ΔE_{orb}	$\Delta\rho_1$	$\Delta\rho_2$
**S2*a*** –62.9 kcal mol^{-1}	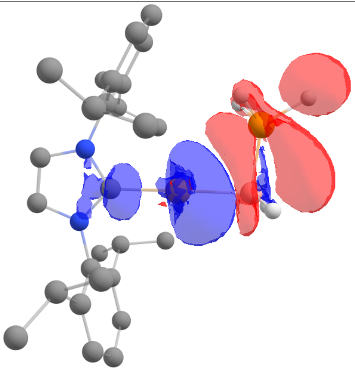 σ-(B=P to Cu(4s)) -37.3 kcal mol^{-1}	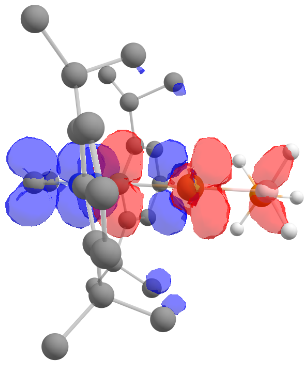 π-(B–H to (Cu-C)*) -3.4 kcal mol^{-1}
**S2b** – 92.2 kcal mol^{-1}	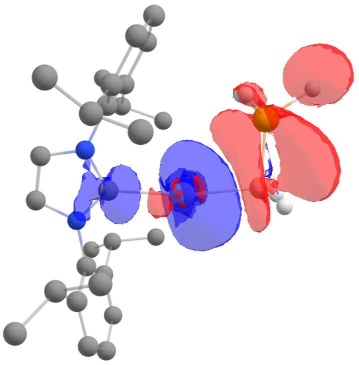 σ-(B=P to Ag(5s)) -66.6 kcal mol^{-1}	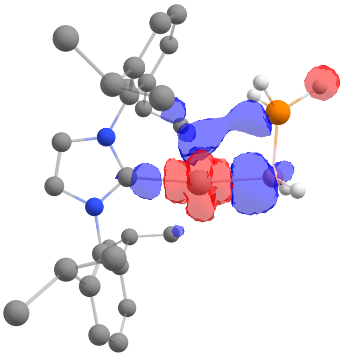 σ-(Ag(4dz^2) to B(sp^2)) -6.7 kcal mol^{-1}
**S2c** – 170.5 kcal mol^{-1}	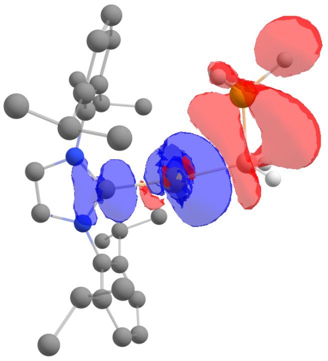 σ-(B=P to Au(6s)) -138.2 kcal mol^{-1}	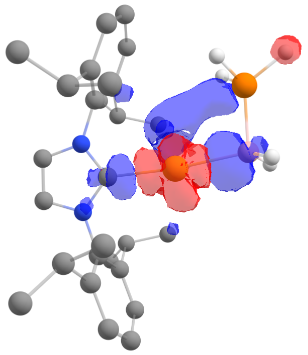 σ-(Au(5dz^2) to B(2p)) -7.7 kcal mol^{-1}

Table S8: Selected deformation density data on complexes **S2a-c**. Charge flow from red to blue.

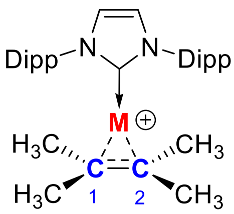

S3 a: **M** = **Cu**;
b: **M** = **Ag**;
c: **M** = **Au**

**Complex** ΔE_{orb}	$\Delta\rho_1$	$\Delta\rho_2$
**S3*a*** –40.0 kcal mol^{-1}	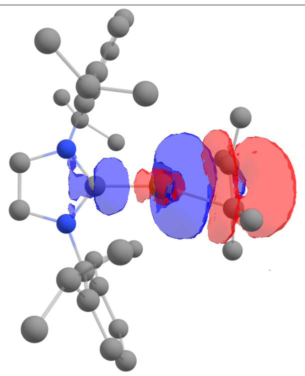 σ-(C=C to Cu(4s)) -17.8 kcal mol^{-1}	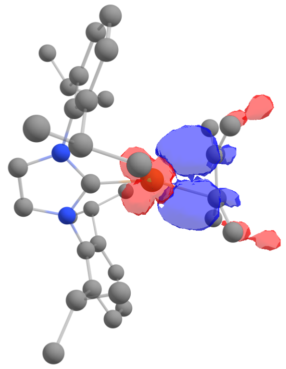 π-(Cu(3d) to (C=C)*) -10.1 kcal mol^{-1}
**S3b** –39.7 kcal mol^{-1}	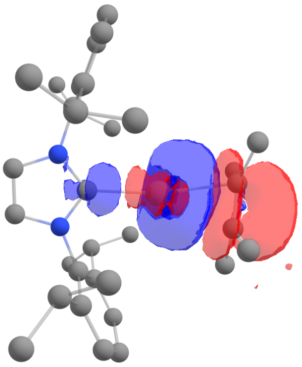 σ-(C=C to Ag(5s)) -22.9 kcal mol^{-1}	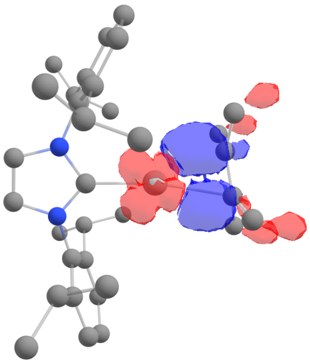 π-(Ag(4d) to (C=C)*) -6.6 kcal mol^{-1}
**S3c** –72.4 kcal mol^{-1}	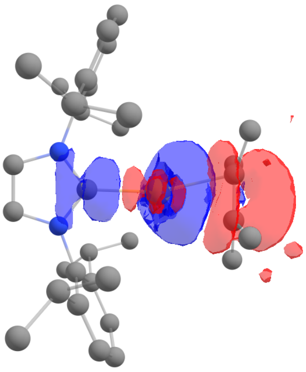 σ-(B=P to Au(6s)) -46.8 kcal mol^{-1}	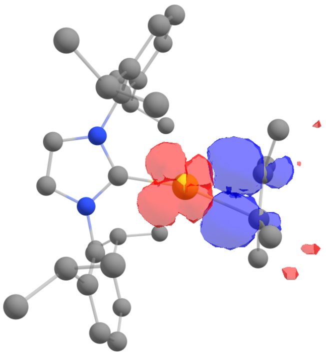 π-(Au(5d) to (C=C)*) -12.2 kcal mol^{-1}

Table S9: Selected deformation density data on complexes **S3a-c**. Charge flow from red to blue.

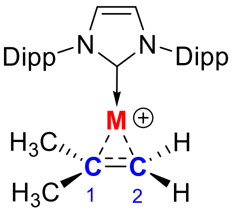

S4 a: **M** = **Cu**;
b: **M** = **Ag**;
c: **M** = **Au**

**Complex**<br>ΔE_{orb}	$\Delta\rho_1$	$\Delta\rho_2$
**S4*a***<br>–38.2 kcal mol^{-1}	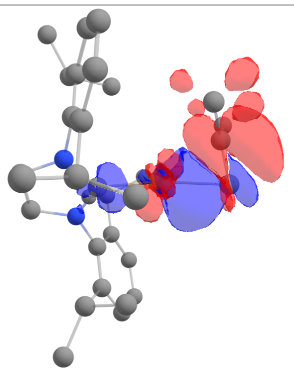<br>σ-(C=C to Cu(4s))<br>-18.6 kcal mol^{-1}	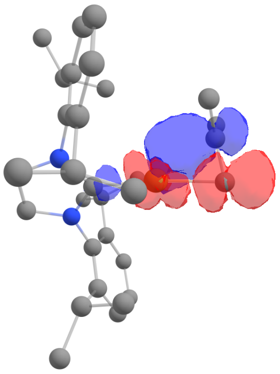<br>π-(Cu(3d) to (C=C)*)<br>-9.7 kcal mol^{-1}
**S4b**<br>–37.4 kcal mol^{-1}	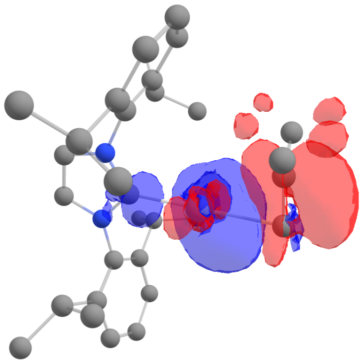<br>σ-(C=C to Ag(5s))<br>-22.9 kcal mol^{-1}	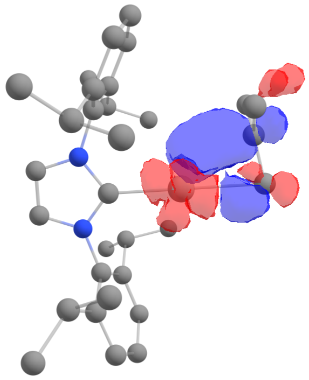<br>σ-(Ag(4d) to (C=C)*)<br>-6.1 kcal mol^{-1}
**S4c**<br>–69.5 kcal mol^{-1}	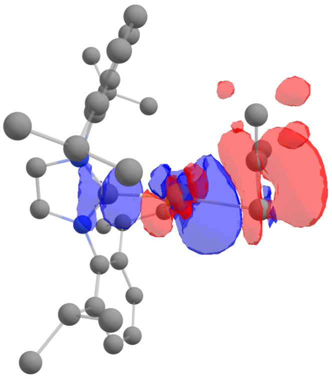<br>σ-(C=C to Au(6s))<br>-45.7 kcal mol^{-1}	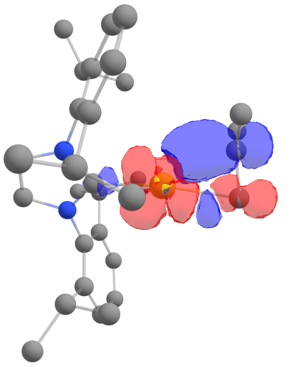<br>π-(Au(5d) to (C=C)*)<br>-12.2 kcal mol^{-1}

Table S10: Selected deformation density data on complexes **S4a-c**. Charge flow from red to blue.

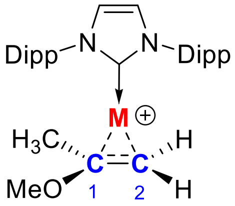

S5 a: M = Cu;
b: M = Ag;
c: M = Au

**Complex** ΔE_{orb}	$\Delta\rho_1$	$\Delta\rho_2$
**S5*a*** –38.9 kcal mol^{-1}	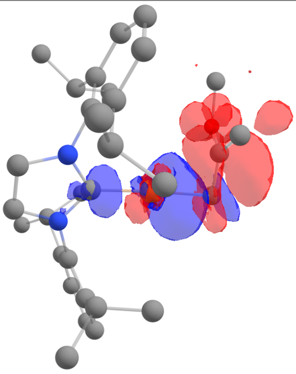 σ-(C=C to Cu(4s)) -22.6 kcal mol^{-1}	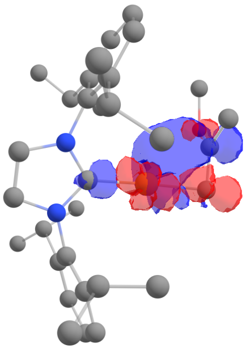 π-(Cu(3d) to (C=C)*) -6.2 kcal mol^{-1}
**S3b** –40.9 kcal mol^{-1}	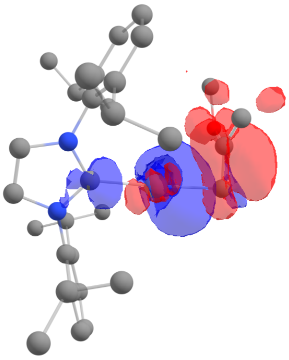 σ-(C=C to Ag(5s)) -26.5 kcal mol^{-1}	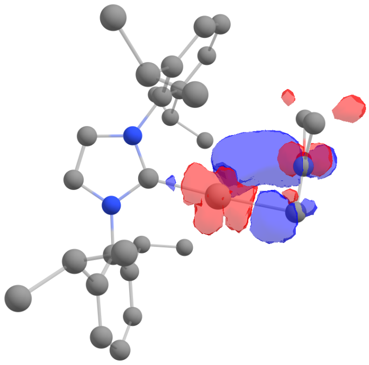 σ-(Ag(4d) to (C=C)*) -5.7 kcal mol^{-1}
**S3c** –71.7 kcal mol^{-1}	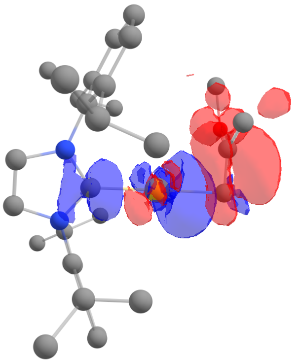 σ-(B=P to Au(6s)) -52.3 kcal mol^{-1}	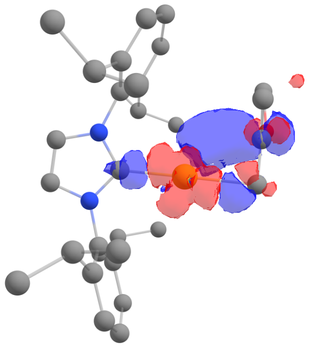 π-(Au(5d) to (C=C)*) -7.6 kcal mol^{-1}

Table S11: Selected deformation density data on complexes **S5a-c**. Charge flow from red to blue.

4.7.2 NBO Data

Bond indices

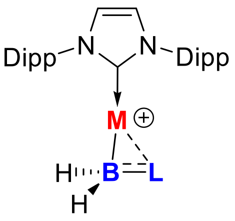

L = cAAC; **3**
L = n/a; **S1**
L = PH_3; **S2**

Wiberg Bond Indices (NBO 6.0)			
a;b;c = Cu;Ag;Au	M–B	M–L	B=L
**3a;3b;3c**	0.25;0.29;0.43	0.15;0.18;0.17	1.47;1.41;1.27
**S1a;S1b;S1c**	0.77;0.80;0.87	-	-
**S2a;S2b;S2c**	0.55;0.60;0.66	0.05;0.06;0.06	1.11;1.08;1.02
Wiberg Bond Indices (NBO 3.1)			
a;b;c = Cu;Ag;Au	M–B	M–L	B=L
**3a;3b;3c**	0.29;0.32;0.46	0.17;0.19;0.18	1.46;1.41;1.27
**S1a;S1b;S1c**	0.80;0.83;0.89	-	-
**S2a;S2b;S2c**	0.58;0.63;0.69	0.07;0.07;0.06	1.11;1.08;1.02
Mayer Bond Indices			
a;b;c = Cu;Ag;Au	M–B	M–L	B=L
**3a;3b;3c**	0.38;0.57;0.55	0.15;0.23;0.18	1.24;1.31;1,27
**S1a;S1b;S1c**	0.97;1.04;1.14	-	-
**S2a;S2b;S2c**	0.62;0.81;0.86	0.22;0.17;0.21	0.93;0.93;0.80

Table S12: Comparison of Bond Indices for the series **3, S1-S2 (a-c)**

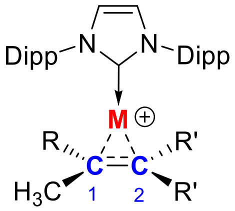

R = Me, R' = Me; **S3**
R = Me, R' = H; **S4**
R = OMe, R' = H; **S5**

Wiberg Bond Indices (NBO 6.0)			
a;b;c = Cu;Ag;Au	M–C^1	M–C^2	C^1=C^2
**S3a;S3b;S3c**	0.14;0.13;0.22	0.14;0.13;0.22	1.68;1.68;1.57
**S4a;S4b;S4c**	0.16;0.13;0.22	0.19;0.17;0.26	1.72;1.73;1.63
**S5a;S5b;S5c**	0.11;0.10;0.13	0.22;0.21;0.30	1.57;1,58;1.47
Wiberg Bond Indices (NBO 3.1)			
a;b;c = Cu;Ag;Au	M–C^1	M–C^2	C^1=C^2
**S3a;S3b;S3c**	0.15;0.15;0.23	0.15;0.14;0.23	1.67;1.68;1.57
**S4a;S4b;S4c**	0.16;0.14;0.22	0.20;0.19;0.27	1.72;1.73;1.62
**S5a;S5b;S5c**	0.11;0.11;0.14	0.23;0.22;0.31	1.57;1.58;1.47
Mayer Bond Indices			
a;b;c = Cu;Ag;Au	M–C^1	M–C^2	C^1=C^2
**S3a;S3b;S3c**	0.23;0.36;0.39	0.25;0.33;0.42	1.51;1.33;1.33
**S4a;S4b;S4c**	0.28;0.28;0.36	0.37;0.40;0.45	1.45;1.44;1.36
**S5a;S5b;S5c**	0.19;0.21;0.28	0.49;0.47;0.45	1.29;1.32;1,25

Table S13: Comparison of Bond Indices for the series **S3-S5 (a-c)**

NPA Charges

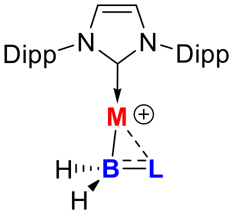

L = cAAC; **3**
L = n/a; **S1**
L = PH_3; **S2**

Natural charges (NBO 6.0)			
a;b;c = Cu;Ag;Au	M	B	C (**3**)/P (**S2**)
**3a;3b;3c**	+0.62;+0.51;+0.34	–0.47;–0.44;–0.53	–0.14;–0.10;+0.04
**S1a;S1b;S1c**	+0.32;+0.25;+0.15	–0.21;–0.16;–0.10	-
**S2a;S2b;S2c**	+0.43;+0.35;+0.24	–1.05;–1.00;–0.94	+0.51;+0.51;+0.51
Natural charges (NBO 3.1)			
a;b;c = Cu;Ag;Au	M	B	C (**3**)/P (**S2**)
**3a;3b;3c**	+0.42;+0.34;+0.17	–0.45;–0.42;–0.51	–0.13;–0.10;+0.05
**S1a;S1b;S1c**	+0.23;+0.16;+0.08	–0.19;–0.14;–0.08	-
**S2a;S2b;S2c**	+0.30;+0.25;+0.14	–1.03;–0.99;–0.92	+0.52;+0.52;+0.52

Table S14: Comparison of natural charges across complexes **3**, **S1**, and **S2** (**a-c**).

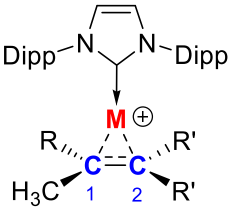

R = Me, R' = Me; **S3**
R = Me, R' = H; **S4**
R = OMe, R' = H; **S5**

Natural charges (NBO 6.0)			
a;b;c = Cu;Ag;Au	M	C^1	C^2
**S3a;S3b;S3c**	+0.72;+0.67;+0.39	–0.11;–0.10;–0.08	–0.11;–0.08;–0.07
**S4a;S4b;S4c**	+0.70;+0.66;+0.52	–0.00;+0.03;+0.02	–0.62;–0.63;–0.61
**S5a;S5b;S5c**	+0.64;+0.62;+0.46	+0.45;+0.44;+0.48	–0.81;–0.78;–0.79
Natural charges (NBO 3.1)			
a;b;c = Cu;Ag;Au	M	C^1	C^2
**S3a;S3b;S3c**	+0.57;+0.56;+0.39	–0.10;–0.10;–0.08	–0.10;–0.07;–0.07
**S4a;S4b;S4c**	+0.57;+0.56;+0.42	–0.00;+0.03;+0.02	–0.61;–0.61;–0.59
**S5a;S5b;S5c**	+0.53;+0.52;+0.36	+0.45;+0.44;+0.49	–0.79;–0.77;–0.78

Table S15: Comparison of natural charges across complexes **S3**, **S4**, and **S5** (**a-c**).

5. Coordinates

3a

SCF (ωB97xD) = -2218.59069091
E(SCF)+ZPE(0 K)= -2217.513381
H(298 K)= -2217.456407
G(298 K)= -2217.600698

H	6.008577	5.998757	6.475273
C	6.986851	7.771573	7.669962
N	7.483493	8.547178	6.611910
B	6.268648	6.481778	7.556083
H	6.130632	5.796648	8.546872
C	7.574839	8.377107	8.968859
C	6.515680	8.935181	9.926358
H	6.990356	9.383149	10.808435
H	5.859368	8.135064	10.271695
H	5.886055	9.700647	9.462506
C	7.588166	8.037895	5.287318
C	6.604242	8.339227	4.330583
C	6.749630	7.861952	3.026489
H	5.991819	8.098006	2.283643
C	7.832856	7.071865	2.674313
H	7.934020	6.702742	1.657913
C	8.779159	6.739326	3.635188
H	9.612863	6.098414	3.361895
C	8.678221	7.208946	4.944036
C	8.051119	9.839503	7.034061
C	9.207896	10.286751	6.142000
H	10.014121	9.550156	6.131874
H	8.875316	10.444197	5.111400
H	9.613313	11.231213	6.518291
C	6.991641	10.955658	7.062033
H	7.388147	11.849019	7.557194
H	6.685302	11.239405	6.052166
H	6.104328	10.627944	7.606703
C	8.384701	7.314281	9.728780
H	8.914231	7.768294	10.576105
H	7.727824	6.527540	10.108820
H	9.122469	6.841776	9.073081
C	8.514248	9.496582	8.459700
H	8.515272	10.371205	9.118888
H	9.543838	9.122515	8.414628
C	5.348239	9.098548	4.705106
H	5.435405	9.358192	5.763146
C	9.712795	6.776747	5.969612
H	9.559095	7.385924	6.862848
C	11.152847	6.999691	5.490490
H	11.856748	6.794176	6.303676
H	11.410769	6.332380	4.661107
H	11.313291	8.027275	5.149710
C	9.494279	5.312153	6.374149
H	8.480960	5.169463	6.757238
H	9.640715	4.646107	5.515820
H	10.206922	5.020126	7.153829
C	4.117945	8.199066	4.559905
H	4.269843	7.240285	5.063367
H	3.244601	8.683234	5.010458
H	3.890853	7.990735	3.507730
C	5.171699	10.393122	3.904832
H	5.001279	10.181863	2.843124
H	4.308052	10.957472	4.274701
H	6.055232	11.034933	3.973682
Cu	4.561570	7.691173	7.743424
C	2.623940	7.844603	7.972453
N	1.622284	7.139297	7.376864
C	0.647816	8.182247	9.046295
C	0.411968	7.331553	8.024649
H	-0.000231	8.591243	9.803880
H	-0.490887	6.840009	7.704743
C	1.798423	6.252059	6.257369
C	2.528528	5.064735	6.453856
C	1.233173	6.606904	5.022502
C	2.728041	4.253756	5.337238
C	1.447630	5.744520	3.946431
C	2.200749	4.590514	4.096872
H	3.306619	3.343340	5.436523
H	1.028461	5.987091	2.975405
H	2.374073	3.942136	3.243841
C	2.698019	9.211981	10.008766
C	3.163469	8.497925	11.119520
C	2.887826	10.589277	9.837379
C	3.872299	9.215871	12.083730
C	3.614066	11.257306	10.822471
C	4.104237	10.576009	11.931581
H	4.260763	8.698657	12.955922
H	3.797907	12.322223	10.726512
H	4.670413	11.113628	12.685582
N	1.997604	8.481646	8.994014
C	0.364965	7.845511	4.838683
H	0.552032	8.521940	5.679324
C	3.032069	4.642094	7.830577
H	3.473618	5.520221	8.310673
C	-1.122442	7.453317	4.855666
H	-1.354267	6.813989	3.997254
H	-1.396732	6.896979	5.755823
H	-1.757462	8.342900	4.797048
C	0.664636	8.627523	3.552768
H	0.070640	9.546597	3.536550
H	1.718127	8.899673	3.476861
H	0.395915	8.054929	2.659380
C	4.137929	3.587939	7.787369
H	3.765209	2.616060	7.443339
H	4.964514	3.909001	7.149734
H	4.538213	3.449132	8.795682
C	1.866904	4.157314	8.708664
H	1.396929	3.269441	8.271528
H	2.235851	3.889977	9.704541
H	1.095390	4.922434	8.833382
C	2.905630	7.007247	11.294774
H	2.305084	6.659201	10.449674
C	4.201280	6.186644	11.281398
H	4.884131	6.499415	12.078939
H	3.971892	5.126706	11.434182
H	4.725831	6.281253	10.325877
C	2.089155	6.743499	12.567138
H	2.649865	7.018430	13.466637

H	1.155985	7.315346	12.566062
H	1.839863	5.680541	12.642331
C	2.336982	11.310707	8.616165
H	1.494647	10.720693	8.238971
C	1.799552	12.707547	8.945301
H	2.604908	13.400439	9.209744
H	1.289905	13.125027	8.071839
H	1.089031	12.679570	9.776592
C	3.384841	11.379694	7.496656
H	3.738134	10.381828	7.218756
H	2.962513	11.853451	6.604284
H	4.250455	11.969027	7.815921

3b

SCF (ωB97xD) = -2168.24924866
E(SCF)+ZPE(0 K)= -2167.173620
H(298 K)= -2167.115881
G(298 K)= -2167.264009

H	6.502601	5.798278	6.796520
C	7.078757	7.956574	7.505742
N	7.480301	8.496281	6.285243
B	6.570321	6.573251	7.722788
H	6.566126	6.128145	8.850755
C	7.495077	8.976223	8.597440
C	6.342126	9.500705	9.461459
H	6.719979	10.207677	10.210451
H	5.850578	8.681904	9.994506
H	5.584470	10.020951	8.868747
C	7.800176	7.686385	5.155710
C	6.854210	7.455569	4.143938
C	7.218155	6.691469	3.032635
H	6.490395	6.517646	2.244343
C	8.483178	6.135738	2.929566
H	8.750954	5.539644	2.062131
C	9.401277	6.333290	3.952295
H	10.384431	5.876823	3.881209
C	9.083609	7.103314	5.069876
C	7.689603	9.954879	6.334407
C	8.740452	10.428236	5.332909
H	9.704376	9.942806	5.502746
H	8.429510	10.218853	4.304832
H	8.879393	11.509064	5.434876
C	6.378001	10.721690	6.082294
H	6.495509	11.780189	6.339118
H	6.078561	10.660589	5.033351
H	5.568444	10.309412	6.690413
C	8.522285	8.326541	9.540674
H	8.923950	9.071214	10.239534
H	8.056728	7.521604	10.115358
H	9.357541	7.894725	8.982858
C	8.149129	10.128301	7.793474
H	7.889555	11.113908	8.194095
H	9.241024	10.039931	7.833988
C	5.431757	7.969170	4.243137
H	5.339940	8.492302	5.199298
C	10.106563	7.245808	6.183397
H	9.759561	8.042828	6.843171
C	11.497249	7.641915	5.672854
H	12.159887	7.858050	6.517327
H	11.962636	6.837540	5.093331
H	11.455599	8.529728	5.033919
C	10.172939	5.958073	7.016420
H	9.187241	5.710993	7.419080
H	10.517418	5.118025	6.402440
H	10.872631	6.075773	7.851843
C	4.432466	6.806900	4.262091
H	4.684852	6.096324	5.053675
H	3.419211	7.175343	4.442121
H	4.423986	6.267002	3.308372
C	5.105957	8.954795	3.114156
H	5.092850	8.449248	2.142070
H	4.121626	9.408970	3.267158
H	5.847130	9.757886	3.054523
C	2.409462	7.765194	8.227565
N	1.333349	7.154250	7.673470
C	0.583064	7.972042	9.562971
C	0.208957	7.262591	8.474617
H	0.035013	8.293003	10.433608
H	-0.737991	6.835959	8.186753
C	1.335102	6.468230	6.412713
C	1.807706	5.152279	6.361328
C	0.835531	7.151522	5.293657
C	1.756091	4.506295	5.123988
C	0.793493	6.457577	4.085914
C	1.248465	5.146304	4.002761
H	2.115435	3.485407	5.042124
H	0.419429	6.950491	3.194932
H	1.215670	4.623916	3.051911
C	2.715135	8.969183	10.361968
C	3.340360	8.224767	11.369270
C	2.818373	10.363104	10.259296
C	4.093028	8.929063	12.310266
C	3.594492	11.017582	11.214870
C	4.222203	10.308530	12.232222
H	4.592954	8.386976	13.106960
H	3.712763	12.094844	11.164198
H	4.821041	10.837120	12.967158
N	1.923724	8.269236	9.390310
C	0.452036	8.621084	5.375778
H	0.177207	8.845530	6.411356
C	2.319805	4.419075	7.590598
H	2.413606	5.142790	8.405089
C	-0.751106	8.985582	4.501291
H	-0.515704	8.914830	3.434540
H	-1.606673	8.334678	4.703986
H	-1.052922	10.019039	4.695883
C	1.671214	9.488218	5.025394
H	1.440217	10.550815	5.153190
H	2.530234	9.242344	5.657462
H	1.963359	9.324471	3.982671
C	3.712083	3.817118	7.369317
H	3.699840	3.040033	6.597648
H	4.438235	4.582342	7.080945
H	4.068280	3.357240	8.296187
C	1.306831	3.354104	8.034067
H	1.177136	2.588782	7.261259
H	1.652159	2.856515	8.945649

H	0.326069	3.796930	8.235797
C	3.211977	6.713192	11.462881
H	2.672117	6.361012	10.578987
C	4.581903	6.024965	11.453224
H	5.175484	6.296437	12.332730
H	4.453684	4.937900	11.463628
H	5.151864	6.287588	10.557542
C	2.387411	6.315635	12.694735
H	2.884239	6.628148	13.619399
H	1.394858	6.777042	12.675626
H	2.259302	5.229469	12.733388
C	2.155311	11.127491	9.123714
H	1.334743	10.512578	8.738429
C	1.548618	12.460071	9.575423
H	2.320270	13.184410	9.855141
H	0.973036	12.902813	8.757044
H	0.881310	12.327706	10.432053
C	3.149729	11.334926	7.973421
H	3.516444	10.374520	7.600822
H	2.675855	11.865983	7.141617
H	4.013600	11.920231	8.306723
Ag	4.502502	7.548341	7.753355

3c

SCF (ωB97xD) = -2157.057314
E(SCF)+ZPE(0 K)= -2155.980055
H(298 K)= -2155.922838
G(298 K)= -2156.068719

H	6.290621	6.125113	5.921287
C	7.153657	7.682434	7.444386
N	7.601686	8.635942	6.569433
B	6.385294	6.432889	7.089899
H	6.425661	5.530615	7.904338
C	7.705904	8.050219	8.840423
C	6.629331	8.373583	9.881810
H	7.099954	8.658325	10.831230
H	6.002088	7.499699	10.058779
H	5.973981	9.190479	9.571256
C	7.730815	8.376350	5.167413
C	6.767947	8.833846	4.255139
C	6.949803	8.574443	2.894893
H	6.212578	8.933032	2.181722
C	8.044293	7.855315	2.442622
H	8.167734	7.654926	1.382542
C	8.977848	7.384456	3.355837
H	9.826405	6.806773	3.000963
C	8.845794	7.636088	4.720253
C	8.120319	9.861563	7.225981
C	9.244891	10.512246	6.425469
H	10.079731	9.824028	6.274208
H	8.893269	10.844085	5.443960
H	9.616527	11.386144	6.969192
C	7.004046	10.893225	7.465213
H	7.358496	11.674693	8.145888
H	6.696143	11.375367	6.535675
H	6.124903	10.422491	7.911481
C	8.549842	6.881776	9.376027
H	9.067041	7.184085	10.294856
H	7.917553	6.018483	9.594542
H	9.302122	6.566427	8.646754
C	8.605397	9.277123	8.560386
H	8.571867	10.012154	9.370534
H	9.648535	8.957710	8.451995
C	5.529490	9.584688	4.699924
H	5.534302	9.596948	5.791788
C	9.882726	7.068821	5.676037
H	9.719295	7.529107	6.652328
C	11.319720	7.396609	5.250384
H	12.022679	7.082003	6.028606
H	11.599239	6.873673	4.329758
H	11.457032	8.468621	5.076997
C	9.693782	5.555084	5.846779
H	8.682503	5.329793	6.193586
H	9.857969	5.035780	4.895692
H	10.411249	5.160260	6.575030
C	4.248192	8.868994	4.262275
H	4.241917	7.832090	4.611272
H	3.371228	9.376055	4.680464
H	4.143609	8.872039	3.171495
C	5.536971	11.029070	4.182012
H	5.430715	11.051903	3.091670
H	4.706427	11.599620	4.611032
H	6.470620	11.544121	4.427753
C	2.398197	7.660419	8.001890
N	1.341336	6.956392	7.521953
C	0.515622	8.146818	9.171512
C	0.185458	7.229755	8.235181
H	-0.068649	8.635311	9.933894
H	-0.748344	6.738972	8.016214
C	1.442841	5.914180	6.536562
C	1.849231	4.644379	6.968385
C	1.127843	6.206977	5.201822
C	1.895903	3.629091	6.012233
C	1.190391	5.156054	4.287525
C	1.563768	3.879197	4.688988
H	2.203036	2.631406	6.309025
H	0.956856	5.337682	3.244345
H	1.608546	3.076655	3.959496
C	2.670397	9.182084	9.904149
C	3.162302	8.563732	11.061373
C	2.954936	10.507838	9.558423
C	4.011721	9.316525	11.872051
C	3.819977	11.213873	10.394913
C	4.348433	10.621511	11.535525
H	4.428842	8.869479	12.769177
H	4.083206	12.238471	10.151665
H	5.026385	11.183676	12.169975
N	1.868602	8.395040	9.012669
C	0.732692	7.610030	4.765548
H	1.272492	8.312819	5.411055
C	2.213203	4.349151	8.415205
H	2.305532	5.300049	8.945975
C	-0.774485	7.851489	4.946123
H	-1.348605	7.128725	4.356504
H	-1.085229	7.761208	5.989120
H	-1.041886	8.856581	4.604503
C	1.128283	7.914367	3.316556

H	1.037173	8.987529	3.124821
H	2.158294	7.617782	3.109538
H	0.472026	7.400813	2.605472
C	3.572342	3.650308	8.533780
H	3.556631	2.650948	8.086527
H	4.358627	4.236728	8.049771
H	3.836494	3.532975	9.589496
C	1.099343	3.546528	9.100485
H	0.963706	2.571634	8.619821
H	1.347684	3.372321	10.152533
H	0.142836	4.077765	9.058904
C	2.788514	7.137183	11.440243
H	2.009862	6.795211	10.752269
C	3.968605	6.169518	11.293585
H	4.807352	6.465658	11.932574
H	3.661640	5.159342	11.584966
H	4.323561	6.131991	10.259112
C	2.192447	7.077661	12.853019
H	2.934585	7.336705	13.615071
H	1.348602	7.766252	12.958807
H	1.838611	6.064970	13.069420
C	2.351916	11.151036	8.320408
H	1.537353	10.505735	7.976344
C	1.745508	12.526425	8.622679
H	2.514593	13.257964	8.890798
H	1.227018	12.909049	7.738240
H	1.027197	12.474858	9.446218
C	3.376546	11.232301	7.184099
H	3.782024	10.242776	6.955684
H	2.909831	11.633364	6.278083
H	4.212097	11.886920	7.453846
Au	4.417705	7.364330	7.546069

4

SCF (ωB97xD) = -1950.017301
E(SCF)+ZPE(0 K)= -1949.020938
H(298 K)= -1948.969795
G(298 K)= -1949.098977

Zn	10.594125	3.411567	12.810772
B	8.479997	3.623209	12.930837
H	8.243642	4.556608	12.202871
H	8.444014	3.800263	14.128760
C	8.122039	2.228777	12.478884
N	7.708083	1.849713	11.243731
C	7.569257	0.377928	11.033867
C	8.137371	-0.174849	12.354369
H	9.174438	-0.493323	12.199266
H	7.577906	-1.049495	12.699414
C	8.102152	0.982317	13.370027
C	7.267016	2.777493	10.234630
C	8.145628	3.192213	9.217093
C	7.641847	3.972403	8.174383
H	8.309962	4.288061	7.378382
C	6.317127	4.377870	8.154731
H	5.941986	4.982921	7.335169
C	5.486248	4.043858	9.214113
H	4.465660	4.414363	9.229522
C	5.939395	3.254515	10.271008
C	9.642677	2.945174	9.276498
H	9.835421	2.312687	10.146253
C	10.372222	4.279743	9.498282
H	9.967125	4.810071	10.364663
H	10.261724	4.932683	8.625843
H	11.440673	4.113408	9.664489
C	10.206424	2.247784	8.032697
H	10.112985	2.884873	7.147007
H	9.699861	1.304128	7.819266
H	11.272505	2.038404	8.170793
C	5.015915	3.031788	11.457647
H	5.484962	2.299126	12.112039
C	3.635991	2.494219	11.060656
H	3.709772	1.587468	10.454003
H	3.064054	3.232903	10.489763
H	3.054416	2.258082	11.957727
C	4.882725	4.328813	12.269762
H	4.406722	5.115695	11.674193
H	5.864213	4.684332	12.591988
H	4.265832	4.157428	13.158379
C	8.391417	-0.100773	9.835679
H	9.444848	0.166841	9.942612
H	8.011095	0.327428	8.904000
H	8.322501	-1.190453	9.760381
C	6.110843	-0.036838	10.799095
H	5.701297	0.474889	9.923701
H	5.471535	0.179394	11.656258
H	6.067526	-1.113635	10.611400
C	6.795887	0.987908	14.191072
H	6.749865	1.883454	14.815979
H	6.760154	0.103835	14.837233
H	5.904802	0.973475	13.560586
C	9.262012	0.897423	14.361508
H	9.223222	1.727016	15.071781
H	10.233738	0.924917	13.856778
H	9.208753	-0.039984	14.926781
B	12.708258	3.623189	12.690787
H	12.944598	4.556649	13.418679
H	12.744285	3.800142	11.492848
C	13.066217	2.228793	13.142856
N	13.480170	1.849837	14.378041
C	13.618988	0.378069	14.588035
C	13.050878	-0.174820	13.267578
H	12.013809	-0.493276	13.422703
H	13.610342	-1.049499	12.922612
C	13.086109	0.982257	12.251819
C	13.921239	2.777703	15.387062
C	13.042624	3.192522	16.404556
C	13.546407	3.972797	17.447202
H	12.878291	4.288532	18.243171
C	14.871132	4.378251	17.466827
H	15.246274	4.983368	18.286339
C	15.702014	4.044138	16.407479
H	16.722606	4.414632	16.392044
C	15.248865	3.254708	15.350650
C	11.545574	2.945493	16.345162
H	11.352827	2.312936	15.475459
C	10.816044	4.280051	16.123259
H	11.221138	4.810290	15.256822

H	10.926559	4.933073	16.995635
H	9.747590	4.113714	15.957078
C	10.981814	2.248215	17.589021
H	11.075257	2.885379	18.474657
H	11.488366	1.304572	17.802532
H	9.915731	2.038837	17.450940
C	16.172347	3.031872	14.164033
H	15.703303	2.299149	13.509708
C	17.552271	2.494343	14.561076
H	17.478492	1.587649	15.167815
H	18.124208	3.233082	15.131898
H	18.133847	2.258121	13.664028
C	16.305534	4.328822	13.351798
H	16.781531	5.115761	13.947295
H	15.324045	4.684306	13.029536
H	16.922431	4.157357	12.463199
C	12.796815	-0.100516	15.786262
H	11.743387	0.167096	15.679297
H	13.177134	0.327767	16.717904
H	12.865723	-1.190191	15.861659
C	15.077397	-0.036687	14.822853
H	15.486943	0.475113	15.698207
H	15.716713	0.179465	13.965677
H	15.120703	-1.113468	15.010643
C	14.392381	0.987774	11.430784
H	14.438409	1.883267	10.805800
H	14.428117	0.103646	10.784700
H	15.283460	0.973394	12.061278
C	11.926257	0.897276	11.260334
H	11.965052	1.726807	10.549989
H	10.954526	0.924808	11.765052
H	11.979525	-0.040179	10.695143

A

SCF (ωB97xD) = -861.409769
E(SCF)+ZPE(0 K)= -860.917429
H(298 K)= -860.892504
G(298 K)= -860.967192

H	5.753877	6.435320	6.026528
C	7.123250	7.691615	7.450799
N	7.651054	8.681284	6.528484
B	6.198656	6.618806	7.144180
H	5.874642	5.829538	8.016188
C	7.758373	8.014166	8.813765
C	6.689311	8.297102	9.884029
H	7.143069	8.497636	10.867157
H	6.026092	7.431433	9.969076
H	6.069823	9.156708	9.612359
C	7.796746	8.413326	5.155019
C	6.832168	8.858814	4.226969
C	7.022741	8.624545	2.862257
H	6.280926	8.983095	2.151082
C	8.124061	7.918266	2.403977
H	8.257947	7.736914	1.340322
C	9.045285	7.424349	3.321350
H	9.895893	6.847179	2.965311
C	8.900270	7.658128	4.687278
C	8.130322	9.873660	7.211270
C	9.254407	10.573345	6.445087
H	10.099166	9.897921	6.279865
H	8.904168	10.920846	5.466867
H	9.611123	11.442109	7.010067
C	7.006478	10.900944	7.484688
H	7.326578	11.654282	8.215646
H	6.719675	11.424144	6.567596
H	6.123392	10.388524	7.875517
C	8.631790	6.854993	9.327795
H	9.118779	7.099143	10.285415
H	8.012911	5.963498	9.466266
H	9.411196	6.603723	8.601177
C	8.631141	9.268231	8.545149
H	8.600390	9.991577	9.370176
H	9.680528	8.971309	8.417499
C	5.559427	9.539089	4.694674
H	5.604332	9.559266	5.784884
C	9.905070	7.071435	5.660833
H	9.723217	7.555566	6.621914
C	11.359109	7.345086	5.259875
H	12.039162	7.007642	6.050531
H	11.637793	6.813153	4.342379
H	11.533662	8.413161	5.090713
C	9.648576	5.571796	5.855453
H	8.624657	5.428360	6.213636
H	9.786558	5.025594	4.913448
H	10.341575	5.154072	6.596622
C	5.450936	10.973934	4.163663
H	5.333713	10.983984	3.072752
H	6.346197	11.557320	4.402757
C	4.319928	8.717960	4.317907
H	3.412990	9.198343	4.704939
H	4.210768	8.623770	3.229936
H	4.397217	7.719196	4.754445
H	4.583160	11.485843	4.596233

S1a.log

SCF (ωB97xD) = -1383.14428128
E(SCF)+ZPE(0 K)= -1382.546502
H(298 K)= -1382.511130
G(298 K)= -1382.613598
Lowest Frequency = 21.1704cm-1

H	6.378988	8.215486	5.108527
B	6.138648	7.711951	6.187666
H	7.131846	7.171617	6.632258
Cu	4.372792	7.764098	7.097635
C	2.630675	7.813617	7.997854
N	1.501723	7.136508	7.675482
C	0.952425	8.304961	9.445633
C	0.461218	7.425071	8.542550
H	0.497391	8.789845	10.293784
H	-0.513604	6.978205	8.434917
C	1.417685	6.241614	6.557479
C	1.606546	4.871310	6.788915
C	1.185172	6.781517	5.287713
C	1.540361	4.022774	5.685006
C	1.129084	5.889450	4.215135

C	1.301902	4.526620	4.411121
H	1.686876	2.956079	5.817556
H	0.957006	6.270488	3.213018
H	1.258756	3.849620	3.563690
C	3.174569	9.400301	9.788204
C	4.056515	8.838331	10.717922
C	3.149980	10.768336	9.479570
C	4.936871	9.705554	11.367903
C	4.048079	11.591523	10.156801
C	4.931319	11.065651	11.093265
H	5.641125	9.306550	12.091698
H	4.066958	12.655239	9.944785
H	5.626128	11.722965	11.606607
N	2.271840	8.530629	9.090585
C	1.027816	8.275438	5.053970
H	1.069080	8.780495	6.023653
C	1.947155	4.344718	8.175177
H	1.561050	5.057836	8.911181
C	-0.330809	8.605744	4.423274
H	-0.428374	8.163455	3.426351
H	-1.155729	8.232847	5.038091
H	-0.444850	9.689067	4.316992
C	2.189056	8.819593	4.211177
H	2.099848	9.904956	4.099266
H	3.153646	8.600451	4.680836
H	2.191741	8.376683	3.209414
C	3.471965	4.279498	8.355476
H	3.912115	3.569612	7.647252
H	3.938651	5.254365	8.182314
H	3.723569	3.951965	9.369911
C	1.299777	2.989980	8.480071
H	1.731177	2.187078	7.873649
H	1.467537	2.725952	9.528490
H	0.220871	3.009611	8.299664
C	4.096351	7.346086	11.006316
H	3.310973	6.861235	10.418858
C	5.433581	6.740455	10.559240
H	6.268586	7.161341	11.129636
H	5.430300	5.657165	10.717181
H	5.618020	6.932454	9.497072
C	3.809489	7.052301	12.484265
H	4.581894	7.475973	13.134620
H	2.845646	7.468883	12.792561
H	3.787265	5.971857	12.657577
C	2.235457	11.315944	8.393774
H	1.376378	10.642661	8.304360
C	1.682919	12.707862	8.716870
H	2.466014	13.472467	8.691116
H	0.932930	12.991412	7.972494
H	1.213384	12.735582	9.704676
C	2.963380	11.313577	7.040182
H	3.325406	10.313730	6.780961
H	2.292691	11.654814	6.244335
H	3.829300	11.983338	7.069190

S1b.log

SCF (ωB97xD) = -1332.80013895
E(SCF)+ZPE(0 K)= -1332.202791
H(298 K)= -1332.167226
G(298 K)= -1332.270679
Lowest Frequency = 20.0406cm-1

H	6.731977	7.678231	4.917950
B	6.477973	7.273672	6.031282
H	7.437249	6.728731	6.532019
Ag	4.582981	7.493367	6.989679
C	2.660421	7.714937	7.966939
N	1.490172	7.107373	7.659458
C	1.015520	8.366024	9.388376
C	0.468337	7.493124	8.510837
H	0.591935	8.908576	10.217707
H	-0.534092	7.109806	8.412147
C	1.352594	6.178216	6.575289
C	1.471140	4.809669	6.856026
C	1.147075	6.683871	5.286698
C	1.365392	3.926879	5.782133
C	1.046262	5.758397	4.246571
C	1.153237	4.396239	4.490822
H	1.460234	2.859595	5.953179
H	0.891713	6.111576	3.231454
H	1.077760	3.692721	3.667613
C	3.307395	9.315577	9.707755
C	4.138244	8.724473	10.666398
C	3.389555	10.667704	9.345605
C	5.079669	9.546137	11.288836
C	4.347638	11.444655	9.995105
C	5.182982	10.890086	10.958321
H	5.745324	9.123688	12.035530
H	4.451003	12.494112	9.740085
H	5.925019	11.511406	11.449903
N	2.348453	8.488843	9.033287
C	1.057796	8.174484	5.001502
H	1.146688	8.710793	5.951045
C	1.773362	4.310176	8.260969
H	1.486129	5.095240	8.968055
C	-0.299550	8.545685	4.390634
H	-0.443147	8.068465	3.415594
H	-1.125042	8.237529	5.039650
H	-0.365273	9.628143	4.242804
C	2.220837	8.634000	4.112474
H	2.180363	9.718285	3.967104
H	3.185688	8.383139	4.564959
H	2.176704	8.160363	3.125904
C	3.282000	4.073223	8.428449
H	3.626571	3.287307	7.748007
H	3.855625	4.978994	8.208554
H	3.508448	3.761651	9.453739
C	0.973019	3.057079	8.632327
H	1.286430	2.185878	8.048245
H	1.132704	2.812363	9.686743
H	-0.099334	3.203779	8.472719
C	4.053064	7.248497	11.021379
H	3.260416	6.795350	10.418559
C	5.357073	6.520324	10.670434
H	6.195220	6.895928	11.267056
H	5.256406	5.448647	10.870017
H	5.608629	6.650788	9.612990
C	3.676888	7.052680	12.495748
H	4.446371	7.457923	13.161231

H	2.731696	7.550249	12.733949
H	3.569909	5.987099	12.721665
C	2.520005	11.246689	8.239393
H	1.633184	10.612248	8.139221
C	2.025260	12.663854	8.547805
H	2.843258	13.391327	8.538729
H	1.305274	12.980065	7.787081
H	1.536543	12.715310	9.525290
C	3.268164	11.203328	6.898025
H	3.582430	10.185431	6.647669
H	2.627964	11.575338	6.091015
H	4.166407	11.828520	6.938342

S1c.log

SCF (ωB97xD) = -1321.62617446
E(SCF)+ZPE(0 K)= -1321.028591
H(298 K)= -1320.992772
G(298 K)= -1321.100251
Lowest Frequency = 3.7502cm-1

H	6.700536	7.922360	5.067362
B	6.387535	7.309226	6.062783
H	7.263102	6.569017	6.450506
Au	4.551796	7.505524	7.002461
C	2.657576	7.706910	7.974371
N	1.489824	7.101209	7.656971
C	1.016054	8.339290	9.401986
C	0.469916	7.474572	8.515454
H	0.592119	8.872535	10.236966
H	-0.531621	7.090198	8.413316
C	1.356276	6.186879	6.559693
C	1.546132	4.821412	6.807564
C	1.092451	6.708456	5.287916
C	1.445976	3.955409	5.719053
C	0.998910	5.800368	4.232801
C	1.171997	4.439033	4.445504
H	1.597791	2.890730	5.866321
H	0.801237	6.166207	3.229723
H	1.102457	3.749254	3.610220
C	3.307618	9.295945	9.722905
C	4.098726	8.720540	10.724029
C	3.437972	10.627780	9.309033
C	5.045516	9.540348	11.339058
C	4.400776	11.404503	9.952750
C	5.194332	10.867742	10.959278
H	5.683043	9.129446	12.116095
H	4.541556	12.438554	9.654117
H	5.941536	11.487477	11.445027
N	2.349077	8.466950	9.050637
C	0.949740	8.200617	5.036167
H	1.019796	8.717589	5.997906
C	1.913030	4.299524	8.187692
H	1.745815	5.103645	8.911062
C	-0.417868	8.541356	4.431258
H	-0.543378	8.086290	3.443271
H	-1.234045	8.188407	5.069126
H	-0.519856	9.624457	4.311748
C	2.099543	8.717761	4.161313
H	2.021625	9.802530	4.035755
H	3.068979	8.491466	4.616166
H	2.075347	8.260580	3.166324
C	3.404821	3.939755	8.242702
H	3.633685	3.125351	7.546957
H	4.027996	4.798194	7.972660
H	3.681535	3.613927	9.250878
C	1.035595	3.117525	8.615831
H	1.204975	2.238132	7.986191
H	1.269626	2.830512	9.645546
H	-0.027845	3.369586	8.563216
C	3.976570	7.256599	11.114419
H	3.155432	6.816907	10.540386
C	5.250197	6.485522	10.742738
H	6.114096	6.854941	11.305627
H	5.129045	5.421634	10.970898
H	5.470937	6.586865	9.675544
C	3.632989	7.097077	12.600679
H	4.433828	7.482052	13.240555
H	2.713277	7.632378	12.855975
H	3.493581	6.039570	12.845715
C	2.617984	11.195813	8.161485
H	1.802213	10.498100	7.947826
C	1.978252	12.543887	8.513149
H	2.732117	13.323695	8.661661
H	1.325881	12.872978	7.698556
H	1.379347	12.476116	9.426333
C	3.476196	11.296753	6.892096
H	3.899499	10.323725	6.623239
H	2.873358	11.656810	6.051810
H	4.306417	11.995764	7.039753

S2a.log

SCF (ωB97xD) = -1726.30258625
E(SCF)+ZPE(0 K)= -1725.674777
H(298 K)= -1725.637316
G(298 K)= -1725.744402
Lowest Frequency = 22.8469cm-1

H	6.301783	8.077990	5.194530
B	6.260666	7.654490	6.333444
H	6.904007	6.644216	6.542771
Cu	4.418883	7.668763	7.188327
C	2.655856	7.682412	8.006674
N	1.493964	7.059887	7.697095
C	1.006749	8.293451	9.445500
C	0.470392	7.422988	8.558283
H	0.575499	8.818646	10.282013
H	-0.527288	7.027472	8.458750
C	1.359380	6.159119	6.588703
C	1.427297	4.780844	6.841470
C	1.200021	6.697564	5.307205
C	1.303354	3.924446	5.749046
C	1.084022	5.796891	4.246311
C	1.130151	4.427680	4.464199
H	1.353544	2.851280	5.898431
H	0.965103	6.176261	3.235887
H	1.040788	3.744074	3.625721
C	3.298746	9.287629	9.728195
C	4.156805	8.726634	10.681912

C	3.385779	10.623553	9.311873
C	5.121405	9.562997	11.246820
C	4.368346	11.417080	9.903690
C	5.225494	10.893699	10.864432
H	5.808711	9.161681	11.985579
H	4.474547	12.453710	9.600258
H	5.986843	11.526083	11.310383
N	2.336451	8.438065	9.087757
C	1.180089	8.195116	5.045409
H	1.255875	8.713033	6.006170
C	1.711144	4.251813	8.239641
H	1.325497	4.980126	8.961091
C	-0.136664	8.633275	4.391846
H	-0.262213	8.182753	3.401739
H	-0.998455	8.347053	5.002650
H	-0.152175	9.720335	4.264796
C	2.394833	8.617035	4.207663
H	2.399654	9.703190	4.069803
H	3.332258	8.328486	4.695088
H	2.371483	8.152580	3.215950
C	3.228722	4.144683	8.461008
H	3.665781	3.417146	7.769181
H	3.728407	5.103166	8.291995
H	3.443929	3.817429	9.483987
C	1.019745	2.917463	8.535610
H	1.450963	2.097863	7.951749
H	1.146924	2.660287	9.591396
H	-0.052057	2.961660	8.320175
C	4.097449	7.256433	11.063496
H	3.245331	6.803104	10.548363
C	5.358537	6.524787	10.583242
H	6.253579	6.914678	11.080623
H	5.287299	5.455799	10.807691
H	5.491086	6.639217	9.501471
C	3.875654	7.069676	12.569458
H	4.716920	7.460126	13.151559
H	2.968870	7.582394	12.904444
H	3.775860	6.006080	12.807132
C	2.506467	11.170856	8.198259
H	1.661226	10.488232	8.066415
C	1.924939	12.550205	8.527086
H	2.703269	13.318357	8.578650
H	1.221300	12.854421	7.746321
H	1.393833	12.542867	9.483555
C	3.286104	11.194272	6.874930
H	3.672888	10.200268	6.624134
H	2.641215	11.530112	6.056044
H	4.137320	11.881216	6.940504
P	7.021622	9.006841	7.437387
H	8.361552	9.524857	7.396809
H	6.990521	8.751735	8.824392
H	6.367018	10.255907	7.417864

S2b.log

SCF (ωB97xD) = -1675.95722005
E(SCF)+ZPE(0 K)= -1675.329479
H(298 K)= -1675.291784
G(298 K)= -1675.400278
Lowest Frequency = 16.6185cm-1

H	6.779203	8.359543	5.284752
B	6.660117	7.811746	6.362628
H	7.357162	6.829460	6.518167
Ag	4.598860	7.612557	7.070131
C	2.634336	7.658980	7.972362
N	1.453422	7.065187	7.684553
C	1.017808	8.337508	9.416412
C	0.448119	7.467521	8.549688
H	0.610925	8.888171	10.248747
H	-0.560771	7.097538	8.466474
C	1.287797	6.151556	6.591072
C	1.391928	4.777597	6.851412
C	1.071708	6.677376	5.312371
C	1.259460	3.911236	5.767335
C	0.948260	5.767814	4.260420
C	1.039260	4.401370	4.484909
H	1.339596	2.840522	5.922226
H	0.788150	6.136738	3.251846
H	0.945001	3.710251	3.653179
C	3.329784	9.271403	9.676545
C	4.141674	8.710941	10.670606
C	3.474429	10.589906	9.225134
C	5.110544	9.533954	11.246682
C	4.459447	11.371042	9.830282
C	5.265686	10.851439	10.835344
H	5.760324	9.132751	12.018791
H	4.606070	12.395301	9.501034
H	6.028464	11.474137	11.292367
N	2.347529	8.438600	9.043757
C	1.000788	8.173025	5.048700
H	1.053409	8.692264	6.010330
C	1.711088	4.259759	8.246003
H	1.377580	5.011460	8.969378
C	-0.327306	8.567067	4.389980
H	-0.431037	8.120077	3.395874
H	-1.181642	8.245521	4.993470
H	-0.381572	9.653593	4.270084
C	2.202489	8.638699	4.215549
H	2.168760	9.724075	4.075736
H	3.147440	8.385963	4.707449
H	2.201241	8.170661	3.225329
C	3.230105	4.099584	8.416611
H	3.617841	3.351170	7.717530
H	3.756381	5.039095	8.223544
H	3.468034	3.773001	9.434656
C	0.980638	2.956111	8.584436
H	1.354467	2.114865	7.991953
H	1.140259	2.703544	9.636953
H	-0.096332	3.041306	8.411544
C	4.027514	7.253686	11.087289
H	3.201826	6.801559	10.529874
C	5.300910	6.483741	10.711281
H	6.169221	6.862930	11.261358
H	5.188286	5.421965	10.952434
H	5.509984	6.572324	9.639689
C	3.704277	7.117099	12.580264
H	4.510213	7.518903	13.203291
H	2.783893	7.650106	12.837748
H	3.575454	6.063061	12.845044

C	2.648083	11.145389	8.076458
H	1.873437	10.413679	7.828570
C	1.937308	12.449721	8.456500
H	2.651497	13.253331	8.663644
H	1.297497	12.783580	7.633891
H	1.312518	12.318352	9.345150
C	3.522223	11.322181	6.826665
H	4.001723	10.378475	6.544871
H	2.915576	11.666159	5.982605
H	4.308978	12.064628	7.001116
P	7.279332	9.083881	7.649136
H	8.600375	9.637236	7.712961
H	7.186999	8.700050	9.002435
H	6.577109	10.304845	7.709438

S2c.log

SCF (ωB97xD) = -1664.77806163
E(SCF)+ZPE(0 K)= -1664.150054
H(298 K)= -1664.112371
G(298 K)= -1664.221640
Lowest Frequency = 14.5536cm-1

H	6.687495	8.216665	5.230968
B	6.547628	7.690506	6.318915
H	7.230825	6.696719	6.477363
Au	4.547045	7.608330	7.088541
C	2.630138	7.676721	7.977689
N	1.456200	7.077688	7.673491
C	1.002902	8.327452	9.418441
C	0.444820	7.462860	8.538918
H	0.586900	8.865525	10.254380
H	-0.560402	7.085582	8.446428
C	1.308664	6.168298	6.574292
C	1.472748	4.798864	6.820379
C	1.060248	6.697116	5.302738
C	1.360813	3.936858	5.730175
C	0.956357	5.792857	4.244951
C	1.102565	4.428463	4.456042
H	1.491424	2.869309	5.874791
H	0.772245	6.164074	3.241324
H	1.025113	3.741654	3.618996
C	3.312785	9.271958	9.699765
C	4.117292	8.704886	10.695769
C	3.471664	10.587383	9.245422
C	5.095381	9.517929	11.269945
C	4.465208	11.358910	9.848822
C	5.266333	10.832281	10.854331
H	5.739944	9.111280	12.043635
H	4.622253	12.381142	9.517751
H	6.036075	11.447496	11.309995
N	2.334143	8.443274	9.056686
C	0.948372	8.192408	5.053545
H	0.989438	8.703142	6.020359
C	1.836083	4.277024	8.201663
H	1.591789	5.055052	8.932196
C	-0.389507	8.559442	4.399707
H	-0.482922	8.119352	3.401484
H	-1.235304	8.211209	5.000503
H	-0.470887	9.645347	4.289762
C	2.138790	8.695110	4.225414
H	2.079537	9.781018	4.097756
H	3.087399	8.456445	4.717052
H	2.147137	8.237972	3.230128
C	3.348918	4.024652	8.289489
H	3.650059	3.240517	7.586653
H	3.917017	4.927868	8.045855
H	3.624210	3.702283	9.299402
C	1.039752	3.027066	8.591900
H	1.306258	2.165201	7.971774
H	1.255088	2.756607	9.630119
H	-0.037716	3.191846	8.496644
C	3.986780	7.249720	11.114244
H	3.159021	6.805845	10.553451
C	5.252664	6.464490	10.744880
H	6.124230	6.837937	11.293866
H	5.128466	5.405600	10.993031
H	5.459543	6.544449	9.672583
C	3.655233	7.120328	12.606001
H	4.462835	7.515111	13.231451
H	2.739676	7.664094	12.858237
H	3.513786	6.068474	12.873011
C	2.650889	11.150734	8.096846
H	1.884939	10.415697	7.832635
C	1.926839	12.443398	8.491723
H	2.633973	13.247261	8.721352
H	1.293528	12.787838	7.668417
H	1.293606	12.292206	9.371244
C	3.531559	11.352060	6.855607
H	4.013648	10.413061	6.563710
H	2.927666	11.706285	6.013880
H	4.313352	12.096001	7.045641
P	7.283327	8.978188	7.568851
H	8.630262	9.445397	7.550688
H	7.217730	8.632622	8.932850
H	6.644960	10.232791	7.622417

S3a.log

SCF (ωB97xD) = -1592.81390002
E(SCF)+ZPE(0 K)= -1592.065921
H(298 K)= -1592.024296
G(298 K)= -1592.138613
Lowest Frequency = 17.1705cm-1

Cu	4.543014	7.566935	7.898674
C	2.680879	7.768317	8.237163
N	1.659805	7.213369	7.553009
C	0.719464	8.396911	9.144090
C	0.442323	7.589424	8.089592
H	0.074373	8.906103	9.841470
H	-0.494198	7.249686	7.678168
C	1.884659	6.360110	6.416920
C	1.932070	4.973921	6.627411
C	2.122757	6.962232	5.175101
C	2.256520	4.176414	5.530158
C	2.440052	6.117474	4.109224
C	2.512470	4.741989	4.285644
H	2.309036	3.099229	5.646540
H	2.630616	6.542590	3.128661

H	2.761422	4.102772	3.444864
C	2.870560	9.242255	10.166713
C	3.393666	8.560286	11.272505
C	3.126035	10.595490	9.899490
C	4.224699	9.283699	12.130701
C	3.964032	11.270287	10.786920
C	4.511257	10.620611	11.888073
H	4.650924	8.793984	13.000789
H	4.192779	12.317121	10.618212
H	5.159652	11.165044	12.566812
N	2.096921	8.494997	9.211521
C	2.054583	8.467278	4.966750
H	1.757187	8.935199	5.910789
C	1.693488	4.363061	8.000518
H	1.135846	5.087626	8.602899
C	0.996820	8.838438	3.919128
H	1.254952	8.445094	2.931125
H	0.014649	8.442536	4.191436
H	0.915634	9.925724	3.832121
C	3.430805	9.032507	4.594677
H	3.380948	10.118477	4.474011
H	4.164680	8.811276	5.377107
H	3.799928	8.604135	3.657373
C	3.030120	4.110519	8.712535
H	3.636439	3.389138	8.154246
H	3.606510	5.035950	8.811655
H	2.862186	3.706847	9.715741
C	0.851145	3.083861	7.943521
H	1.391874	2.259641	7.468521
H	0.597224	2.760976	8.956901
H	-0.079365	3.241812	7.391851
C	3.097081	7.094161	11.547910
H	2.400486	6.731285	10.785292
C	4.371133	6.247627	11.437084
H	5.122702	6.557171	12.170292
H	4.147091	5.190652	11.607412
H	4.814670	6.344499	10.440221
C	2.419246	6.907344	12.911614
H	3.079937	7.202316	13.732555
H	1.506509	7.504948	12.984584
H	2.153629	5.856911	13.061291
C	2.562228	11.289855	8.668521
H	1.697956	10.715278	8.319631
C	2.068198	12.710775	8.961935
H	2.893913	13.390364	9.193690
H	1.555216	13.114346	8.084629
H	1.370143	12.725869	9.803146
C	3.597726	11.290203	7.534831
H	3.904610	10.270892	7.279132
H	3.183695	11.755077	6.634801
H	4.491351	11.852538	7.826200
C	7.053318	7.931634	9.351843
H	8.089267	8.285414	9.410380
H	6.444627	8.632490	9.934573
H	7.009729	6.956268	9.833509
C	6.802976	9.285817	7.247520
H	6.458333	10.071587	7.925404
H	7.869104	9.461845	7.061932
H	6.276065	9.404455	6.300353
C	6.242466	6.731525	5.672165
H	5.333228	6.200574	5.367803
H	6.242859	7.711801	5.198467
H	7.090790	6.166788	5.268137
C	6.462091	5.380218	7.781345
H	7.461175	4.972620	7.587045
H	6.289024	5.338930	8.857068
H	5.741262	4.714661	7.298522
C	6.372322	6.771108	7.181921
C	6.633664	7.924381	7.895302

S3b.log

SCF (ωB97xD) = -1542.46107062
E(SCF)+ZPE(0 K)= -1541.714361
H(298 K)= -1541.672031
G(298 K)= -1541.789783
Lowest Frequency = 16.5903cm-1

C	6.885405	8.062783	8.036863
C	2.571311	7.654899	8.205494
N	1.527124	7.132546	7.535408
C	0.643631	8.310845	9.164806
C	0.328732	7.526422	8.104047
H	0.023938	8.823492	9.882326
H	-0.622513	7.211366	7.707026
C	1.686994	6.297927	6.375070
C	1.653067	4.906493	6.550192
C	1.923201	6.916921	5.140903
C	1.852447	4.119562	5.416399
C	2.116616	6.081843	4.038564
C	2.078777	4.700666	4.173547
H	1.833729	3.038603	5.502813
H	2.297085	6.519596	3.061642
H	2.227362	4.069505	3.303471
C	2.836254	9.115431	10.133868
C	3.368831	8.435270	11.236912
C	3.129010	10.454278	9.835448
C	4.233330	9.150366	12.068004
C	4.003863	11.120212	10.694279
C	4.550140	10.475840	11.798769
H	4.665873	8.662380	12.935932
H	4.260960	12.156296	10.499466
H	5.224614	11.013629	12.457173
N	2.024284	8.377496	9.204052
C	1.995194	8.427645	4.977807
H	1.791944	8.892707	5.947766
C	1.483708	4.277790	7.925607
H	0.989553	5.006539	8.576595
C	0.934118	8.939906	3.995308
H	1.099621	8.550073	2.986278
H	-0.070435	8.642847	4.308744
H	0.965889	10.031849	3.938222
C	3.404290	8.863120	4.551660
H	3.465729	9.953512	4.487614
H	4.156772	8.520784	5.272723
H	3.670184	8.451589	3.572978
C	2.860923	3.975497	8.538306
H	3.406354	3.252944	7.922031
H	3.469121	4.883040	8.617149
H	2.751314	3.552627	9.541776

C	0.605168	3.022368	7.908779
H	1.092036	2.189953	7.391657
H	0.407728	2.693448	8.932747
H	-0.354148	3.211617	7.419729
C	3.056378	6.975539	11.527801
H	2.331791	6.622424	10.787204
C	4.313146	6.106547	11.383671
H	5.086535	6.402390	12.099941
H	4.074013	5.053802	11.559877
H	4.735389	6.192464	10.375671
C	2.416759	6.803268	12.911716
H	3.106178	7.089898	13.711801
H	1.516020	7.415261	13.010018
H	2.139283	5.757647	13.072750
C	2.565045	11.148892	8.604831
H	1.740356	10.542347	8.217355
C	1.992566	12.534274	8.927340
H	2.774652	13.238638	9.226602
H	1.501411	12.950588	8.043431
H	1.257390	12.483201	9.734885
C	3.627006	11.232413	7.499074
H	3.990067	10.236706	7.222107
H	3.211388	11.702340	6.602371
H	4.484948	11.829548	7.826341
C	6.822932	6.870291	7.345984
C	7.131668	8.155053	9.529398
H	8.168984	8.472064	9.691336
H	6.488203	8.915188	9.984084
H	6.982794	7.219526	10.066776
C	7.009241	9.409635	7.351273
H	6.526445	10.186821	7.949320
H	8.071414	9.670973	7.274371
H	6.586321	9.444013	6.346863
Ag	4.618069	7.478270	7.858502
C	7.007681	5.516695	8.006066
H	8.048653	5.197969	7.875257
H	6.789779	5.505363	9.073991
H	6.379515	4.762929	7.523028
C	6.887347	6.789924	5.833072
H	7.911741	6.532887	5.538170
H	6.237416	5.994439	5.457434
H	6.620696	7.716045	5.324508

S3c.log

SCF (ωB97xD) = -1531.26577410
E(SCF)+ZPE(0 K)= -1530.518718
H(298 K)= -1530.476421
G(298 K)= -1530.594995
Lowest Frequency = 15.3688cm-1

C	6.826159	7.951364	7.938932
C	2.587963	7.709440	8.230486
N	1.575019	7.172418	7.527519
C	0.636097	8.324105	9.145519
C	0.359254	7.540385	8.072913
H	-0.008412	8.822108	9.851174
H	-0.576475	7.210134	7.652548
C	1.793011	6.334504	6.378196
C	1.871262	4.949626	6.578028
C	1.997573	6.953536	5.138592
C	2.159786	4.164669	5.461617
C	2.279574	6.121045	4.054559
C	2.359852	4.743356	4.213662
H	2.232885	3.087308	5.568722
H	2.440915	6.557239	3.073666
H	2.579967	4.114044	3.357499
C	2.791309	9.152807	10.181701
C	3.291274	8.465380	11.295021
C	3.089962	10.492206	9.896855
C	4.126708	9.178093	12.156373
C	3.933350	11.155992	10.787976
C	4.446552	10.506486	11.904855
H	4.534271	8.686002	13.034003
H	4.194572	12.193457	10.605918
H	5.098561	11.042292	12.587059
N	2.012892	8.415692	9.221534
C	1.950646	8.462787	4.957651
H	1.693407	8.919434	5.918998
C	1.712299	4.319330	7.953253
H	1.293258	5.071136	8.629941
C	0.867643	8.874282	3.952063
H	1.083175	8.488382	2.950978
H	-0.114520	8.498326	4.251741
H	0.810122	9.964280	3.881746
C	3.326791	9.008337	4.551335
H	3.296627	10.098961	4.471236
H	4.087220	8.741177	5.293828
H	3.644111	8.607555	3.583462
C	3.082064	3.912294	8.516730
H	3.559126	3.161982	7.877385
H	3.752987	4.775050	8.586767
H	2.973033	3.483932	9.517764
C	0.741821	3.132327	7.942150
H	1.134025	2.292144	7.361251
H	0.577238	2.773823	8.962125
H	-0.225920	3.412993	7.517764
C	2.975982	7.002336	11.564733
H	2.293582	6.646334	10.786081
C	4.243957	6.141257	11.483533
H	4.974236	6.434336	12.244521
H	3.999671	5.086415	11.639432
H	4.719252	6.239075	10.501197
C	2.265188	6.823598	12.912591
H	2.908928	7.118680	13.746928
H	1.354138	7.426266	12.962998
H	1.990834	5.775175	13.060265
C	2.570691	11.191968	8.649985
H	1.790282	10.566576	8.204539
C	1.932383	12.548669	8.971724
H	2.670768	13.267498	9.339607
H	1.483710	12.974860	8.070074
H	1.150598	12.451116	9.729797
C	3.688160	11.336755	7.606673
H	4.094852	10.360045	7.324169
H	3.307039	11.820622	6.702218
H	4.509375	11.947901	7.996065
C	6.638670	6.753211	7.254234
C	7.221191	8.014364	9.401316
H	8.298993	8.207657	9.459672

H	6.712695	8.843440	9.902130
H	7.014376	7.102435	9.959692
C	7.033512	9.276754	7.231425
H	6.652152	10.097909	7.843381
H	8.110307	9.437014	7.100614
H	6.564868	9.339790	6.249520
Au	4.578605	7.516783	7.897888
C	6.825938	5.396588	7.906886
H	7.857834	5.066842	7.737391
H	6.644414	5.387238	8.981458
H	6.166982	4.657299	7.444672
C	6.624072	6.666551	5.740539
H	7.617154	6.348177	5.401730
H	5.907525	5.911645	5.405539
H	6.386401	7.605391	5.241863

S4a.log

SCF (ωB97xD) = -1514.19563884
E(SCF)+ZPE(0 K)= -1513.504732
H(298 K)= -1513.466028
G(298 K)= -1513.574447
Lowest Frequency = 23.6989cm-1

H	6.591823	6.393493	6.627141
C	6.612071	8.176660	7.810111
H	6.612956	6.137883	8.465079
Cu	4.549728	7.420515	7.816185
C	2.688821	7.684135	8.149540
N	1.626430	7.187697	7.484861
C	0.779025	8.359011	9.135697
C	0.441147	7.592575	8.069392
H	0.175255	8.870279	9.867781
H	-0.518376	7.296933	7.677437
C	1.750206	6.349752	6.322067
C	1.723063	4.959051	6.503090
C	1.930454	6.966166	5.077785
C	1.884177	4.169458	5.365087
C	2.088183	6.128856	3.971722
C	2.063939	4.747897	4.113430
H	1.871370	3.088703	5.454789
H	2.231661	6.563512	2.987493
H	2.185685	4.114437	3.240920
C	2.971046	9.089820	10.133113
C	3.511418	8.343113	11.187545
C	3.214708	10.460368	9.950824
C	4.345019	9.017307	12.083189
C	4.053913	11.084061	10.873862
C	4.615743	10.369353	11.927229
H	4.781229	8.476600	12.917439
H	4.269714	12.142133	10.774850
H	5.263151	10.875520	12.635798
N	2.160213	8.406374	9.161606
C	1.992801	8.477137	4.916756
H	1.745756	8.938360	5.878675
C	1.590943	4.335308	7.884912
H	1.116359	5.067914	8.546421
C	0.971989	8.988330	3.892694
H	1.188907	8.618223	2.886142
H	-0.042103	8.674635	4.154530
H	0.992582	10.081239	3.852802
C	3.417083	8.917574	4.551582
H	3.480561	10.008039	4.488376
H	4.137151	8.577494	5.305240
H	3.724224	8.503121	3.586163
C	2.981700	4.028597	8.462388
H	3.509656	3.302954	7.835188
H	3.596154	4.934119	8.523482
H	2.896458	3.610193	9.470113
C	0.707585	3.083059	7.895803
H	1.177259	2.247586	7.367945
H	0.536768	2.757795	8.925718
H	-0.263824	3.274731	7.432354
C	3.216977	6.863595	11.383585
H	2.548633	6.532154	10.582669
C	4.496720	6.024499	11.281289
H	5.217520	6.293265	12.059991
H	4.267391	4.960706	11.389961
H	4.982206	6.165226	10.308872
C	2.493672	6.616458	12.714280
H	3.122644	6.886522	13.568123
H	1.572702	7.202505	12.777729
H	2.233832	5.558678	12.813744
C	2.633719	11.222412	8.767967
H	1.708781	10.721418	8.463711
C	2.270736	12.672412	9.104026
H	3.159913	13.288518	9.269866
H	1.722106	13.118966	8.270432
H	1.642841	12.732546	9.996903
C	3.597487	11.164848	7.572970
H	3.790956	10.130870	7.269002
H	3.178223	11.695250	6.712581
H	4.553264	11.636411	7.828759
C	6.519072	6.823208	7.624328
C	6.738057	8.792125	9.180197
H	7.732736	9.241588	9.277061
H	6.007566	9.596611	9.321652
H	6.608383	8.060722	9.980179
C	6.774791	9.120779	6.647720
H	6.103690	9.979733	6.734298
H	7.798311	9.512276	6.660525
H	6.608189	8.630140	5.686771

S4b.log

SCF (ωB97xD) = -1463.84493802
E(SCF)+ZPE(0 K)= -1463.154503
H(298 K)= -1463.115481
G(298 K)= -1463.225931
Lowest Frequency = 13.5300cm-1

H	6.909675	7.725055	6.118277
C	6.969414	8.212277	8.198002
H	7.089441	6.287365	7.278312
C	2.634298	7.644241	8.087349
N	1.560663	7.114789	7.469860
C	0.746724	8.311460	9.121690
C	0.387897	7.515301	8.084752
H	0.158776	8.831056	9.860587
H	-0.578838	7.195734	7.731074

C	1.655986	6.282981	6.300208
C	1.611395	4.891099	6.470348
C	1.822826	6.906892	5.057415
C	1.740096	4.108835	5.323481
C	1.947069	6.075861	3.942038
C	1.905600	4.694647	4.073087
H	1.711688	3.027840	5.404534
H	2.073775	6.517045	2.958231
H	2.000860	4.066555	3.193433
C	2.970064	9.129782	9.989964
C	3.511274	8.473847	11.103432
C	3.268518	10.457945	9.652451
C	4.407902	9.196866	11.893316
C	4.173780	11.133531	10.472321
C	4.741412	10.508525	11.577424
H	4.848604	8.727876	12.767729
H	4.435459	12.162554	10.247404
H	5.440078	11.053078	12.204341
N	2.127721	8.378347	9.100536
C	1.875884	8.418567	4.895406
H	1.747847	8.879261	5.880252
C	1.494518	4.260202	7.850402
H	1.002179	4.980056	8.513053
C	0.734290	8.925254	4.004076
H	0.819258	8.535912	2.984923
H	-0.239321	8.621559	4.398537
H	0.754370	10.017419	3.945260
C	3.241414	8.867576	4.358378
H	3.287829	9.958732	4.294829
H	4.054383	8.529580	5.012621
H	3.431035	8.463774	3.359132
C	2.893066	3.987749	8.427111
H	3.438145	3.278452	7.795682
H	3.482722	4.908285	8.491781
H	2.818229	3.561582	9.432359
C	0.643558	2.986067	7.860181
H	1.137888	2.161276	7.337904
H	0.476585	2.659600	8.890306
H	-0.330560	3.150415	7.391758
C	3.163214	7.034268	11.449712
H	2.393932	6.688798	10.751983
C	4.378594	6.114688	11.275180
H	5.196265	6.403302	11.943942
H	4.109400	5.078753	11.500158
H	4.753959	6.149223	10.246451
C	2.579820	6.923645	12.864492
H	3.316122	7.196611	13.626675
H	1.711471	7.576620	12.987476
H	2.265399	5.895192	13.063062
C	2.663662	11.137888	8.432808
H	1.837721	10.517576	8.070986
C	2.078800	12.514689	8.771325
H	2.858115	13.230436	9.050162
H	1.557601	12.923472	7.901255
H	1.366585	12.452237	9.598402
C	3.688808	11.237763	7.294866
H	4.053048	10.247326	7.000107
H	3.237667	11.705641	6.414678
H	4.550774	11.843357	7.594371
C	6.896867	7.349461	7.139914
C	7.225407	7.734684	9.601745
H	8.226996	8.061442	9.903525
H	6.515052	8.184955	10.302498
H	7.176079	6.647538	9.687875
C	6.964855	9.706454	8.015338
H	6.261049	10.179663	8.707793
H	7.962224	10.091262	8.256465
H	6.717862	10.001673	6.993535
Ag	4.675193	7.503514	7.633847

S4c.log

SCF (ωB97xD) = -1452.64672111
E(SCF)+ZPE(0 K)= -1451.956014
H(298 K)= -1451.916995
G(298 K)= -1452.028396
Lowest Frequency = 16.4011cm-1

H	6.830902	6.537570	6.488904
C	6.862748	8.176889	7.871431
H	6.909520	6.071076	8.279577
C	2.632062	7.670448	8.156605
N	1.583637	7.171418	7.478906
C	0.724405	8.333795	9.133049
C	0.395495	7.570549	8.060964
H	0.114869	8.843104	9.861439
H	-0.560299	7.272402	7.662272
C	1.724800	6.342262	6.311430
C	1.726442	4.951861	6.487560
C	1.907994	6.971273	5.073933
C	1.916108	4.171851	5.347160
C	2.089777	6.142917	3.965502
C	2.093653	4.760574	4.100362
H	1.928945	3.090475	5.433171
H	2.231875	6.585937	2.984693
H	2.236991	4.134627	3.225714
C	2.920728	9.089131	10.124283
C	3.451947	8.367497	11.200986
C	3.204817	10.440167	9.878500
C	4.309480	9.054236	12.062276
C	4.073196	11.075891	10.766305
C	4.619761	10.390530	11.846070
H	4.737994	8.535970	12.914471
H	4.323485	12.120738	10.615851
H	5.288370	10.905950	12.527900
N	2.104460	8.385400	9.170488
C	1.934584	8.483946	4.918215
H	1.753482	8.937652	5.898262
C	1.588543	4.312678	7.861381
H	1.188187	5.063646	8.550612
C	0.823838	8.970617	3.978554
H	0.963194	8.588338	2.962712
H	-0.160381	8.645108	4.326741
H	0.823982	10.063139	3.924663
C	3.314607	8.962210	4.446575
H	3.341791	10.054633	4.392526
H	4.100424	8.633487	5.136035
H	3.553972	8.569922	3.453334
C	2.965433	3.889688	8.395951
H	3.422853	3.140375	7.741900

H	3.648188	4.744320	8.458415
H	2.871065	3.455968	9.396268
C	0.609509	3.132913	7.865515
H	0.989582	2.286785	7.285270
H	0.454444	2.780089	8.888919
H	-0.361162	3.418227	7.451057
C	3.129138	6.901774	11.446364
H	2.474736	6.551252	10.641852
C	4.394588	6.035169	11.408207
H	5.091090	6.305419	12.208153
H	4.136065	4.979813	11.533397
H	4.917377	6.143996	10.451416
C	2.368978	6.721222	12.767245
H	2.980852	7.022974	13.623017
H	1.453690	7.319566	12.782494
H	2.094398	5.671811	12.907116
C	2.637883	11.177906	8.674185
H	1.777279	10.613216	8.301284
C	2.134577	12.582022	9.028474
H	2.955785	13.254968	9.293206
H	1.621378	13.021281	8.168671
H	1.434597	12.555645	9.867804
C	3.671438	11.233064	7.539392
H	3.961411	10.227255	7.218018
H	3.261977	11.759607	6.671986
H	4.572668	11.765461	7.863664
C	6.755504	6.844339	7.529779
C	7.077553	8.621132	9.295624
H	8.100797	9.002254	9.389780
H	6.398877	9.438802	9.559999
H	6.941571	7.807172	10.010198
C	7.015105	9.246949	6.822328
H	6.404595	10.122215	7.058400
H	8.061562	9.572857	6.817488
H	6.761626	8.889481	5.822514
Au	4.620632	7.427932	7.821409

S5a.log

SCF (ωB97xD) = -1589.38236936
E(SCF)+ZPE(0 K)= -1588.685928
H(298 K)= -1588.646381
G(298 K)= -1588.757329
Lowest Frequency = 17.4939cm-1

H	6.597651	7.134144	6.085286
C	6.865651	7.911494	8.045990
H	6.527279	5.897298	7.474225
Cu	4.558924	7.376762	7.582640
C	2.729744	7.684696	8.022421
N	1.625838	7.197534	7.419823
C	0.878618	8.415424	9.085363
C	0.477233	7.628215	8.057649
H	0.320263	8.957922	9.830753
H	-0.504845	7.338673	7.720902
C	1.678059	6.331374	6.272730
C	1.879209	4.962247	6.485821
C	1.563440	6.910677	5.000350
C	1.968324	4.150103	5.353561
C	1.659249	6.054260	3.904268
C	1.859560	4.689261	4.078959
H	2.124260	3.082350	5.471794
H	1.578554	6.455830	2.900079
H	1.930000	4.040919	3.211747
C	3.125900	9.159555	9.931673
C	3.511222	8.554560	11.137617
C	3.569382	10.424195	9.524437
C	4.396226	9.264192	11.950488
C	4.443848	11.097677	10.381384
C	4.857299	10.522757	11.576980
H	4.721757	8.835212	12.892106
H	4.797276	12.088683	10.111786
H	5.533949	11.063847	12.230694
N	2.261202	8.429804	9.047613
C	1.404755	8.413422	4.818856
H	0.968865	8.822807	5.736663
C	2.020177	4.362019	7.876186
H	1.857142	5.154241	8.614139
C	0.459331	8.781721	3.670411
H	0.886821	8.532753	2.694385
H	-0.501452	8.268042	3.761733
H	0.272678	9.859168	3.674116
C	2.779259	9.074140	4.628748
H	2.672425	10.158047	4.520497
H	3.436829	8.882197	5.483652
H	3.271696	8.689447	3.729774
C	3.440667	3.823284	8.093726
H	3.668633	3.010216	7.397459
H	4.187057	4.611716	7.941387
H	3.553826	3.437943	9.111512
C	0.966915	3.276900	8.133717
H	1.097684	2.422099	7.463267
H	1.047319	2.907075	9.160059
H	-0.044760	3.665465	7.988148
C	3.022210	7.166979	11.526791
H	2.099694	6.966650	10.972594
C	4.044919	6.099193	11.110723
H	4.996188	6.251772	11.632200
H	3.678756	5.099179	11.361500
H	4.233089	6.128672	10.031752
C	2.687005	7.051392	13.017893
H	3.583142	7.103552	13.643896
H	2.002836	7.842037	13.337153
H	2.211290	6.087331	13.217063
C	3.127619	11.063635	8.217077
H	2.454886	10.370562	7.703169
C	2.344596	12.358152	8.473194
H	2.970846	13.116707	8.953260
H	1.982985	12.774786	7.528858
H	1.481482	12.177713	9.120025
C	4.320489	11.306237	7.284267
H	4.863082	10.377169	7.078216
H	3.978387	11.715049	6.329428
H	5.025069	12.024268	7.717464
C	6.482693	6.933613	7.148113
C	7.022544	7.660353	9.516825
H	8.061883	7.832570	9.814046
H	6.385606	8.326361	10.108669
H	6.759015	6.629914	9.752462
O	7.153193	9.109997	7.555511

C	7.473508	10.199376	8.425472
H	8.398515	10.001368	8.972389
H	6.652847	10.400269	9.118805
H	7.616260	11.055126	7.768647

S5b.log

SCF (ωB97xD) = -1539.03220295
E(SCF)+ZPE(0 K)= -1538.336359
H(298 K)= -1538.296480
G(298 K)= -1538.409062
Lowest Frequency = 14.5166cm-1

H	6.944783	7.274781	6.038007
C	7.144447	8.107105	7.980856
H	7.029202	6.059489	7.442396
Ag	4.690125	7.336930	7.542857
C	2.679303	7.615332	8.051160
N	1.566786	7.129347	7.466422
C	0.847202	8.374679	9.124937
C	0.428043	7.586160	8.105362
H	0.301481	8.927613	9.871934
H	-0.560279	7.306926	7.778262
C	1.599597	6.280690	6.305478
C	1.526389	4.892897	6.495653
C	1.745008	6.884673	5.050223
C	1.608789	4.092905	5.356645
C	1.823960	6.036735	3.943706
C	1.757345	4.658405	4.094914
H	1.557952	3.013920	5.453198
H	1.936707	6.461903	2.951248
H	1.818537	4.016830	3.221981
C	3.111191	9.112921	9.933497
C	3.638593	8.464619	11.058345
C	3.441681	10.427090	9.573958
C	4.535139	9.187598	11.848546
C	4.335360	11.108626	10.402218
C	4.877450	10.495937	11.526958
H	4.963955	8.723495	12.731331
H	4.608464	12.132632	10.165641
H	5.565751	11.044961	12.161702
N	2.229527	8.377868	9.070625
C	1.826385	8.392869	4.868997
H	1.690295	8.867062	5.846333
C	1.423157	4.281522	7.885644
H	0.982271	5.028807	8.554179
C	0.707798	8.909343	3.954494
H	0.806310	8.516229	2.938059
H	-0.277021	8.620197	4.331710
H	0.743628	10.000871	3.891690
C	3.208046	8.811573	4.348639
H	3.268882	9.900119	4.256929
H	4.003997	8.483405	5.027451
H	3.410486	8.379008	3.363898
C	2.823196	3.950984	8.426849
H	3.319889	3.214627	7.786592
H	3.454352	4.844987	8.468741
H	2.756264	3.534472	9.436802
C	0.515707	3.047514	7.931822
H	0.955950	2.197473	7.401722
H	0.364927	2.736169	8.969162
H	-0.463394	3.253031	7.490845
C	3.284122	7.028514	11.410739
H	2.505639	6.687447	10.721325
C	4.495586	6.106117	11.218939
H	5.322322	6.394501	11.876859
H	4.228335	5.070474	11.447502
H	4.854386	6.140740	10.183942
C	2.717882	6.917065	12.832113
H	3.464835	7.181501	13.586943
H	1.855313	7.575094	12.967969
H	2.398895	5.890033	13.030510
C	2.893835	11.085418	8.317385
H	2.132605	10.426417	7.889439
C	2.215324	12.426804	8.620414
H	2.929464	13.167038	8.994292
H	1.766416	12.834807	7.710516
H	1.426604	12.312828	9.369041
C	3.999961	11.243720	7.264846
H	4.474091	10.282969	7.034187
H	3.588196	11.649551	6.336202
H	4.776986	11.932579	7.613325
C	6.863327	7.078434	7.104252
C	7.365199	7.898458	9.450948
H	8.388797	8.185060	9.712843
H	6.678634	8.501690	10.053599
H	7.221593	6.849461	9.708098
O	7.280759	9.324128	7.468372
C	7.400413	10.462791	8.323742
H	8.309511	10.407585	8.927708
H	6.520754	10.559607	8.966488
H	7.462179	11.318190	7.653879

S5c.log

SCF (ωB97xD) = -1527.83612458
E(SCF)+ZPE(0 K)= -1527.140136
H(298 K)= -1527.100156
G(298 K)= -1527.214526
Lowest Frequency = 12.4748cm-1

H	6.872868	7.261513	6.024025
C	7.104361	7.995045	8.004902
H	6.901631	5.981877	7.360705
Au	4.638843	7.380106	7.564458
C	2.683761	7.673768	8.029784
N	1.584253	7.176350	7.434730
C	0.846307	8.406586	9.097799
C	0.440294	7.612946	8.076451
H	0.291683	8.950784	9.844469
H	-0.542777	7.320182	7.746076
C	1.636497	6.310849	6.286774
C	1.762656	4.933313	6.504195
C	1.614485	6.899791	5.015878
C	1.866099	4.121787	5.373940
C	1.723746	6.042907	3.920695
C	1.847562	4.669973	4.097554
H	1.966642	3.047663	5.494051
H	1.716536	6.454149	2.916472
H	1.930769	4.021263	3.231742

C	3.103357	9.151331	9.931829
C	3.567109	8.504828	11.085375
C	3.498846	10.441456	9.555030
C	4.470221	9.205358	11.887235
C	4.391101	11.104292	10.400195
C	4.874630	10.492587	11.551474
H	4.854420	8.741813	12.790468
H	4.709674	12.113056	10.154335
H	5.565181	11.026238	12.196849
N	2.227984	8.427722	9.051791
C	1.523495	8.405444	4.820965
H	1.267143	8.861318	5.783082
C	1.817618	4.329134	7.898501
H	1.645251	5.127819	8.627443
C	0.419264	8.793917	3.830267
H	0.642436	8.444668	2.817650
H	-0.546393	8.374539	4.125702
H	0.320781	9.882296	3.785582
C	2.882504	8.977563	4.392104
H	2.822397	10.064905	4.283432
H	3.659277	8.749780	5.130056
H	3.197780	8.558434	3.431221
C	3.207662	3.744837	8.185442
H	3.440164	2.922404	7.501630
H	3.985587	4.507557	8.070797
H	3.255241	3.356976	9.207564
C	0.717159	3.280652	8.104882
H	0.853156	2.419978	7.443003
H	0.734887	2.911102	9.134255
H	-0.273219	3.700936	7.908899
C	3.143185	7.090641	11.449608
H	2.339669	6.789224	10.770406
C	4.302307	6.104757	11.247150
H	5.153643	6.360776	11.887127
H	3.986624	5.087971	11.498206
H	4.640671	6.107083	10.205427
C	2.586819	7.012245	12.876836
H	3.357244	7.225182	13.624281
H	1.769136	7.722922	13.024703
H	2.206198	6.006698	13.076241
C	3.013633	11.102643	8.274795
H	2.317568	10.420662	7.777347
C	2.252204	12.401452	8.568626
H	2.901471	13.151333	9.031372
H	1.861630	12.828844	7.640837
H	1.410725	12.224086	9.244166
C	4.178635	11.341910	7.304943
H	4.707689	10.409069	7.081527
H	3.809066	11.754941	6.362092
H	4.899120	12.055265	7.719504
C	6.740574	7.018832	7.076025
C	7.306793	7.707675	9.462864
H	8.349010	7.911039	9.730383
H	6.663878	8.334628	10.088961
H	7.085774	6.662589	9.675929
O	7.335396	9.208080	7.543997
C	7.485546	10.313956	8.441666
H	8.365272	10.185209	9.076332
H	6.585343	10.435478	9.050216
H	7.619824	11.183363	7.801712

S6

SCF (ωB97xD) = -1579.745139
E(SCF)+ZPE(0 K)= -1579.003690
H(298 K)= -1578.961482
G(298 K)= -1579.077436

C	6.589841	7.876203	7.804455
Cu	4.586556	7.684392	7.959884
C	2.694599	7.769706	8.267375
N	1.685121	7.193938	7.573658
C	0.691334	8.333374	9.162396
C	0.449450	7.529346	8.099813
H	0.026969	8.817938	9.858943
H	-0.470865	7.164358	7.674029
C	1.913923	6.351955	6.433471
C	1.956019	4.964290	6.631934
C	2.138306	6.959578	5.193509
C	2.248488	4.171713	5.523359
C	2.422909	6.120849	4.114595
C	2.482092	4.744287	4.278080
H	2.305035	3.094056	5.633274
H	2.617289	6.554332	3.138480
H	2.718494	4.109996	3.429759
C	2.785406	9.233802	10.220674
C	3.301381	8.566112	11.337421
C	2.991312	10.599025	9.976179
C	4.051240	9.320356	12.241545
C	3.746218	11.307291	10.909999
C	4.271521	10.674352	12.030749
H	4.477802	8.836843	13.115025
H	3.938713	12.363632	10.754701
H	4.863819	11.241798	12.741595
N	2.066400	8.468897	9.242027
C	2.106483	8.468009	5.006428
H	1.812827	8.926468	5.956053
C	1.750579	4.354935	8.010972
H	1.163156	5.061507	8.606943
C	1.065026	8.881705	3.958949
H	1.319863	8.496407	2.966353
H	0.069660	8.508534	4.218584
H	1.014648	9.972621	3.886771
C	3.500428	9.002972	4.657024
H	3.478456	10.093562	4.564959
H	4.226542	8.735073	5.430005
H	3.859817	8.587355	3.710039
C	3.099061	4.167666	8.720403
H	3.723389	3.447742	8.182069
H	3.657658	5.106069	8.774184
H	2.946820	3.795620	9.739299
C	0.963245	3.040924	7.972836
H	1.539313	2.235573	7.506196
H	0.728808	2.719197	8.991967
H	0.023942	3.149781	7.422550
C	3.099708	7.075785	11.561080
H	2.436895	6.696770	10.776890
C	4.430503	6.323797	11.432284
H	5.148064	6.654431	12.190456

H	4.274756	5.247647	11.556759
H	4.882163	6.492829	10.450201
C	2.421361	6.793988	12.907579
H	3.052397	7.101443	13.747791
H	1.468176	7.324730	12.991599
H	2.227984	5.722233	13.015269
C	2.480208	11.264344	8.707348
H	1.655680	10.659131	8.316239
C	1.927315	12.672307	8.952594
H	2.717892	13.377410	9.228458
H	1.460984	13.052942	8.038997
H	1.176627	12.676122	9.748389
C	3.583324	11.281920	7.638640
H	3.946376	10.272400	7.423440
H	3.208255	11.717648	6.706650
H	4.438796	11.876769	7.975408
C	7.145156	7.877504	9.229279
H	8.223623	8.100473	9.214789
H	6.683166	8.642351	9.873293
H	7.030358	6.911581	9.729932
C	6.856434	9.228484	7.142183
H	6.463393	10.071593	7.730456
H	7.939508	9.403057	7.046010
H	6.430874	9.304744	6.136537
C	6.166245	6.570149	5.421579
H	5.205271	6.101499	5.164293
H	6.207906	7.538688	4.912353
H	6.937059	5.932842	4.964418
C	6.461193	5.142012	7.685438
H	7.455386	4.710629	7.496646
H	6.305855	5.107158	8.770409
H	5.740658	4.453343	7.225178
B	6.398841	6.606003	7.013869

S7

SCF (ωB97xD) = -1566.468327
E(SCF)+ZPE(0 K)= -1565.735676
H(298 K)= -1565.692531
G(298 K)= -1565.810114

Cu	4.558590	7.541865	7.889510
C	2.639027	7.734640	8.241790
N	1.596012	7.230518	7.532135
C	0.649028	8.326897	9.176749
C	0.372880	7.580639	8.081731
H	0.005480	8.803560	9.898440
H	-0.562514	7.266449	7.647511
C	1.776440	6.431631	6.355393
C	1.825785	5.038632	6.498504
C	1.950663	7.078463	5.126861
C	2.014864	4.280469	5.344237
C	2.138063	6.276104	4.000738
C	2.162501	4.892735	4.106079
H	2.078249	3.199482	5.417617
H	2.296675	6.743963	3.033723
H	2.325902	4.286220	3.220281
C	2.763618	9.114349	10.261949
C	3.221795	8.403470	11.376742
C	3.035661	10.473756	10.058695
C	3.941892	9.113246	12.338262
C	3.758797	11.138933	11.047456
C	4.201039	10.467286	12.180185
H	4.329765	8.590021	13.207151
H	4.006074	12.187593	10.916695
H	4.776931	10.998547	12.932041
N	2.030396	8.408662	9.252158
C	2.027669	8.591598	5.015102
H	1.748150	9.019821	5.982582
C	1.792527	4.385601	7.869951
H	1.345633	5.096447	8.572803
C	1.050887	9.146227	3.971487
H	1.302098	8.803177	2.961901
H	0.020480	8.841637	4.183984
H	1.091559	10.240450	3.964290
C	3.473809	9.018209	4.729346
H	3.558790	10.109892	4.722226
H	4.160616	8.628077	5.488803
H	3.807168	8.642969	3.755254
C	3.229285	4.111282	8.339114
H	3.716673	3.380190	7.685485
H	3.842670	5.018408	8.314584
H	3.231511	3.716830	9.361423
C	0.936765	3.115133	7.909396
H	1.376762	2.311629	7.309302
H	0.865529	2.746520	8.937860
H	-0.077126	3.299057	7.538509
C	3.034505	6.901599	11.508060
H	2.355619	6.572363	10.715418
C	4.375746	6.190100	11.283661
H	5.095579	6.457316	12.065481
H	4.242217	5.103379	11.299082
H	4.815448	6.465370	10.318436
C	2.397091	6.511157	12.846671
H	3.047919	6.762203	13.691335
H	1.438256	7.018483	12.998027
H	2.223162	5.430525	12.878032
C	2.660517	11.166922	8.759770
H	1.870636	10.580337	8.279173
C	2.106431	12.579835	8.971329
H	2.872038	13.262140	9.355602
H	1.759642	12.989870	8.017237
H	1.265915	12.583364	9.673403
C	3.872724	11.172737	7.816215
H	4.269192	10.163393	7.660616
H	3.599738	11.593323	6.841913
H	4.686677	11.773255	8.235944
C	7.056098	8.931591	8.955870
H	8.098242	9.297790	8.916950
H	6.439828	9.831410	9.144370
H	6.977277	8.303872	9.856288
C	6.741999	9.144224	6.273863
H	6.137148	10.068479	6.346790
H	7.782922	9.494869	6.157424
H	6.474355	8.659071	5.323943
C	6.172994	5.721456	6.057727
H	5.357434	4.977230	6.135821
H	5.910819	6.386468	5.221050
H	7.060287	5.145624	5.737087

C	6.800319	5.475541	8.681586
H	7.722691	4.919549	8.436379
H	6.987386	5.968679	9.646548
H	6.036664	4.694403	8.860090
B	6.477799	6.522713	7.458568
B	6.669094	8.138975	7.570454

S8

SCF (ωB97xD) = -1596.260421
E(SCF)+ZPE(0 K)= -1595.515593
H(298 K)= -1595.473326
G(298 K)= -1595.589181

Cu	4.559291	7.781928	7.969412
C	2.694284	7.827917	8.273755
N	1.713003	7.240024	7.557241
C	0.688455	8.310169	9.177109
C	0.470591	7.527005	8.091929
H	0.008655	8.756617	9.884300
H	-0.439312	7.148832	7.655107
C	1.962761	6.446089	6.383425
C	2.050945	5.053710	6.528959
C	2.137020	7.107079	5.161364
C	2.340045	4.312813	5.383179
C	2.428952	6.318273	4.046816
C	2.530488	4.938390	4.155971
H	2.418247	3.233029	5.447987
H	2.574272	6.790761	3.080380
H	2.755564	4.342779	3.277402
C	2.754673	9.243424	10.267877
C	3.228348	8.570200	11.401294
C	2.969802	10.609016	10.032946
C	3.946261	9.321549	12.333383
C	3.694174	11.312749	10.995147
C	4.175721	10.676672	12.133636
H	4.331898	8.839399	13.226178
H	3.886237	12.371152	10.853998
H	4.733923	11.243442	12.871624
N	2.056888	8.482813	9.266363
C	2.031626	8.618105	5.024229
H	1.715862	9.033398	5.986656
C	1.899534	4.379399	7.884381
H	1.317040	5.043167	8.532091
C	0.975350	9.019841	3.986876
H	1.253217	8.692407	2.980465
H	0.001602	8.583211	4.224784
H	0.865647	10.107821	3.961435
C	3.399104	9.228995	4.693998
H	3.328055	10.317754	4.615289
H	4.126494	8.993853	5.478550
H	3.788548	8.845526	3.745257
C	3.277758	4.196056	8.536090
H	3.916025	3.554810	7.919009
H	3.785985	5.157097	8.661828
H	3.181080	3.731684	9.522352
C	1.145891	3.047233	7.811298
H	1.728672	2.275875	7.298634
H	0.944321	2.679525	8.821134
H	0.190998	3.154683	7.289884
C	3.023675	7.077587	11.607887
H	2.351400	6.708572	10.826595
C	4.356379	6.332436	11.450660
H	5.079178	6.648378	12.209819
H	4.211081	5.252494	11.547587
H	4.793689	6.528637	10.464754
C	2.366742	6.767308	12.958337
H	3.011213	7.049579	13.796339
H	1.418822	7.300726	13.069082
H	2.167909	5.695159	13.043174
C	2.495955	11.289424	8.757679
H	1.729720	10.657419	8.296847
C	1.855734	12.657678	9.017681
H	2.587117	13.389613	9.373422
H	1.427856	13.051499	8.091581
H	1.057118	12.588713	9.760998
C	3.656959	11.404885	7.758177
H	4.067729	10.417491	7.516483
H	3.319598	11.866105	6.825037
H	4.464567	12.019325	8.169901
C	7.226654	8.021475	8.905866
H	8.309770	7.951112	8.758225
H	6.985943	9.033440	9.242532
H	6.932107	7.317176	9.682650
C	6.842602	8.776606	6.638912
H	6.533111	9.760445	7.002493
H	7.924151	8.796463	6.467867
H	6.346018	8.575138	5.692035
C	5.837399	5.984228	5.760058
H	5.077342	5.197637	5.833006
H	5.410128	6.795651	5.165620
H	6.661638	5.545587	5.180522
C	6.747056	5.172154	8.207944
H	7.819241	5.213603	8.442244
H	6.226791	5.230809	9.170918
H	6.537504	4.187300	7.784851
B	6.365232	6.330380	7.204585
N	6.518359	7.732398	7.638861

6. References

[S1] C. Müller, D. M. Andrada, I.-A. Bischoff, M. Zimmer, V. Huch, N. Steinbrück, A. Schäfer, *Organometallics* **2019**, *38*, 5, 1052-1061.

[S2] M. Arrowsmith, J. Mattock, S. Hagspiel, I. Krummenacher, A. Vargas, H. Braunschweig, *Angew. Chem. Int. Ed.* **2018**, *57*, 15272–15275.

[S3] (a) SHELXTL v5.1, Bruker AXS, Madison, WI, 1998. (b) SHELX-2013, G.M. Sheldrick, *Acta Cryst.* **2015**, *C71*, 3-8.

[S4] A.L. Spek (2003, 2009) PLATON, A Multipurpose Crystallographic Tool, Utrecht University, Utrecht, The Netherlands. See also A.L. Spek, *Acta. Cryst.*, 2015, **C71**, 9-18.

[S5] Frisch, M. J.; Trucks, G. W.; Schlegel, H. B.; Scuseria, G. E.; Robb, M. A.; Cheeseman, J. R.; Scalmani, G.; Barone, V.; Mennucci, B.; Petersson, G. A.; Nakatsuji, H.; Caricato, M.; Li, X.; Hratchian, H. P.; Izmaylov, A. F.; Bloino, J.; Zheng, G.; Sonnenberg, J. L.; Hada, M.; Ehara, M.; Toyota, K.; Fukuda, R.; Hasegawa, J.; Ishida, M.; Nakajima, T.; Honda, Y.; Kitao, O.; Nakai, H.; Vreven, T.; Montgomery, J. A., Jr.; Peralta, J. E.; Ogliaro, F.; Bearpark, M.; Heyd, J. J.; Brothers, E.; Kudin, K. N.; Staroverov, V. N.; Kobayashi, R.; Normand, J.; Raghavachari, K.; Rendell, A.; Burant, J. C.; Iyengar, S. S.; Tomasi, J.; Cossi, M.; Rega, N.; Millam, J. M.; Klene, M.; Knox, J. E.; Cross, J. B.; Bakken, V.; Adamo, C.; Jaramillo, J.; Gomperts, R.; Stratmann, R. E.; Yazyev, O.; Austin, A. J.; Cammi, R.; Pomelli, C.; Ochterski, J. W.; Martin, R. L.; Morokuma, K.; Zakrzewski, V. G.; Voth, G. A.; Salvador, P.; Dannenberg, J. J.; Dapprich, S.; Daniels, A. D.; Farkas, Ö.; Foresman, J. B.; Ortiz, J. V.; Cioslowski, J.; Fox, D. J. *Gaussian 09, Revision D.01*; Gaussian, Inc., Wallingford, CT, 2009.

[S8] J.-D. Chai, M. Head-Gordon, *Phys. Chem. Chem. Phys.* **2008**, *10*, 6615–6620.

[S9] *NBO 6.0*. Glendening, E. D.; Badenhoop, J. K.; Reed, A. E.; Carpenter, J. E.; Bohmann, J. A.; Morales, C. M.; Landis, C. R.; Weinhold, F. Theoretical Chemistry Institute, University of Wisconsin, Madison (2013).

[S10] J. Tomasi, B. Mennucci, R. Cammi, *Chem. Rev.* **2005**, *105*, 2999–3094.

[S11] M. P. Mitoraj, A. Michalak, T. Ziegler, *J. Chem. Theory Comput.* **2009**, *5*, 962–975.

[S12] F. Neese, F. Wennmohs, U. Becker, C. Riplinger, *J. Chem. Phys.* **2020**, *152*, 224108.

[S13] F. Neese, *WIREs Comput Mol Sci* **2018**, *8*, e1327.

[S14] J.-D. Chai, M. Head-Gordon, *J. Chem. Phys.* **2008**, *128*, 084106.

[S15] S. Grimme, J. Antony, S. Ehrlich, H. Krieg, *J. Chem. Phys.* **2010**, *132*, 154104.

[S16] F. Weigend, R. Ahlrichs, *Phys. Chem. Chem. Phys.* **2005**, *7*, 3297–3305.

[S17] AIMAll (Version 19.10.12), Todd A. Keith, TK Gristmill Software, Overland Park KS, USA, 2019 (aim.tkgristmill.com)

[S18] F. Cortés-Guzmán, R. F. W. Bader, *Coord. Chem. Rev.* **2005**, *249*, 633–662.

[S19] Gupta, R.; Rezabal, E.; Hasrack, G.; Frison, G. *Chem. Eur. J.* **2020**, *26*, 17230-17241.

[S20] Chi, C,l Pan, S.; Meng, L.; Luo, M.; Zhao, L.; Zhou, M.; Frenking, G, *Angew. Chem. Int. Ed.* **2019**, *58,* 1732

[S21] P. Pyykko, M. Atsumi, *Chem. Eur. J.* **2009**, *15*, 186–197.
